# Composition and function of the C1b/C1f region in the ciliary central apparatus

**DOI:** 10.1038/s41598-021-90996-9

**Published:** 2021-06-03

**Authors:** Ewa Joachimiak, Anna Osinka, Hanan Farahat, Bianka Świderska, Ewa Sitkiewicz, Martyna Poprzeczko, Hanna Fabczak, Dorota Wloga

**Affiliations:** 1 Laboratory of Cytoskeleton and Cilia Biology, Nencki Institute of Experimental Biology Polish Academy of Sciences, 3 Pasteur Street, 02-093 Warsaw, Poland; 2 Mass Spectrometry Laboratory, Institute of Biochemistry and Biophysics Polish Academy of Sciences, 5a Pawinski Street, 02-106 Warsaw, Poland; 3 Present Address: Department of Immunology, Medical University of Warsaw, 5 Nielubowicz Street, 02-097 Warsaw, Poland

**Keywords:** Cytoskeleton, Cilia

## Abstract

Motile cilia are ultrastructurally complex cell organelles with the ability to actively move. The highly conserved central apparatus of motile 9 × 2 + 2 cilia is composed of two microtubules and several large microtubule-bound projections, including the C1b/C1f supercomplex. The composition and function of C1b/C1f subunits has only recently started to emerge. We show that in the model ciliate *Tetrahymena thermophila*, C1b/C1f contains several evolutionarily conserved proteins: Spef2A, Cfap69, Cfap246/LRGUK, Adgb/androglobin, and a ciliate-specific protein Tt170/TTHERM_00205170. Deletion of genes encoding either Spef2A or Cfap69 led to a loss of the entire C1b projection and resulted in an abnormal vortex motion of cilia. Loss of either Cfap246 or Adgb caused only minor alterations in ciliary motility. Comparative analyses of wild-type and C1b-deficient mutant ciliomes revealed that the levels of subunits forming the adjacent C2b projection but not C1d projection are greatly reduced, indicating that C1b stabilizes C2b. Moreover, the levels of several IFT and BBS proteins, HSP70, and enzymes that catalyze the final steps of the glycolytic pathway: enolase ENO1 and pyruvate kinase PYK1, are also reduced in the C1b-less mutants.

## Introduction

Motile cilia and structurally homologous eukaryotic flagella are complex biological nanomachines that propagate fluids and particles along the surface of ciliated epithelia, and power cell motility of protists, zoospores, and male gametes. The cytoskeletal scaffold of motile cilia, the axoneme, is typically composed of nine microtubule doublets arranged around the cilium’s circumference and two single microtubules positioned in its center (9 × 2 + 2). Both the outer doublets and the central microtubules serve as scaffolds for periodically attached multi-protein complexes. Outer and inner dynein arms, radial spokes, and the nexin-dynein regulatory complex are major complexes that are repeatedly docked onto the outer doublet microtubules^1,2^.

The central microtubules, C1 and C2, are linked by so-called bridge and are a docking site for C1a-C1f and C2a-C2e complexes, called projections, and together form so-called central apparatus (CA). The CA projections differ in their size, architecture, protein composition, and likely function^3,4^. In consequence, the CA is asymmetric, which could be important for the generation of asymmetric waveforms.

In *Chlamydomonas,* loss of the entire CA causes flagella paralysis (mutants *pf15*^5^ and *pf19*^6^), while losses of single projections or their parts disturb the flagellar waveform, amplitude or beat frequency to varying extents, depending upon the affected projection or even its part. For instance, flagella of the hydin mutant lacking C2b and a part of C2c are mostly paralyzed^7^. Flagella of the *pf6* mutant that lack C1a^8^ or those of the *pf16* mutant that lack the entire C1a-c-e supercomplex^9,10^ twitch ineffectively at a low frequency and with slightly modified waveform. Smaller structural defects within the C1a-c-e supercomplex such as a loss of a part of the C1c (*fap76* mutant), small parts of C1c and C1e (*fap216*), entire C1c-e (*fap81*), or minor defects of C1a (*fap92*) only reduce the beat frequency and lead to flagella asynchrony^10^. In *fap46* and *fap74* mutants, flagella lack the entire C1d projection and the sheath between C1d and C1b (recently described as C1f^3^) which causes a range of defects including reduced beat frequency, twitching, and even paralysis. Moreover, flagella that are able to beat, frequently struggle to initiate the next effective stroke^11,12^. In contrast, a mutation in the Cpc1 subunit of C1b leads to a loss of the entire C1b projection and neighboring C1f, and reduces the beat frequency but does not affect the waveform^13,14^. Thus, each projection contributes to the overall ciliary motility in a unique way^12^. Because projections are interconnected, it is likely that a subunit loss in one projection could affect also the stability/functionality of other projections. The specific functions of individual projections remain obscure.

It has been proposed that the mechano-chemical signals originating at the CA are transmitted through the radial spokes to the inner dynein arms and regulate their activity^15^. Oda and colleagues^16^ showed that the expression of the C-terminally tagged radial spoke proteins partly rescues the motility defects of *Chlamydomonas* flagella lacking C1a (*pf6* mutant) but has no effect on the movement of flagella lacking C1b projection (*cpc1* mutant). Thus, likely the interaction between C1a and radial spokes is based on a mechanical collision. Such a transient physical contact between radial spoke head and a projection could involve electrostatic interactions between the negatively charged surface of the radial spoke head and CA projection^17^. Whether and how other projections interact with the radial spokes is less clear. In order to reveal such interactions, it is essential to identify all protein components of the CA projections and determine their individual roles in the context of ciliary motility.

Early comparative analyses of the *Chlamydomonas* flagella isolated from wild-type and CA-less mutants revealed that the CA is composed of at least 25 proteins^18^. This number was significantly extended by recent comprehensive proteomic analyses^19,20^ and detailed genetic, biochemical, and microscopic studies of selected projections^7,10,12^.

The vast majority of data concerning the CA was obtained using *Chlamydomonas* as a model. However, a significant number of the CA proteins are not present in other ciliated species^19,20^. Thus, it will be informative to learn more about the composition and functions of the CA subunits in other species.

In *Chlamydomonas*, C1b and C1f (a “sheath material” extending between C1d and C1b projections^3^), missing in the *cpc1* mutant are composed of CPC1/SPEF2, FAP42, FAP69, HSP70, and enolase^13,14^. All those proteins co-purify as a 16S complex^21^. Recent proteomic analyses suggest that FAP39, FAP174, FAP246, phosphoglycerate mutase, WD-domain containing CHLREDRAFT_170023, and an ankyrin domain-containing CHLREDRAFT_177061 could also build a part of either C1b or C1f or be loosely associated with these structures^19,20^. A *Chlamydomonas* FAP42, the adenylate/guanylate kinase-like protein with a predicted molecular mass of approximately 270 kDa, has obvious orthologs only in unicellular green algae (*Chlorophyceae*). This raises a question of the protein composition of C1b/C1f projections in cilia assembled by other species.

Here we use genetic, biochemical, and microscopic approaches to identify the components of the C1b and C1f projections in *Tetrahymena* and investigate their function in cilia beating regulation.

## Results

### Identification of the proteins positioned in close proximity to *Tetrahymena* Spef2 ortholog

**Figure 1:**
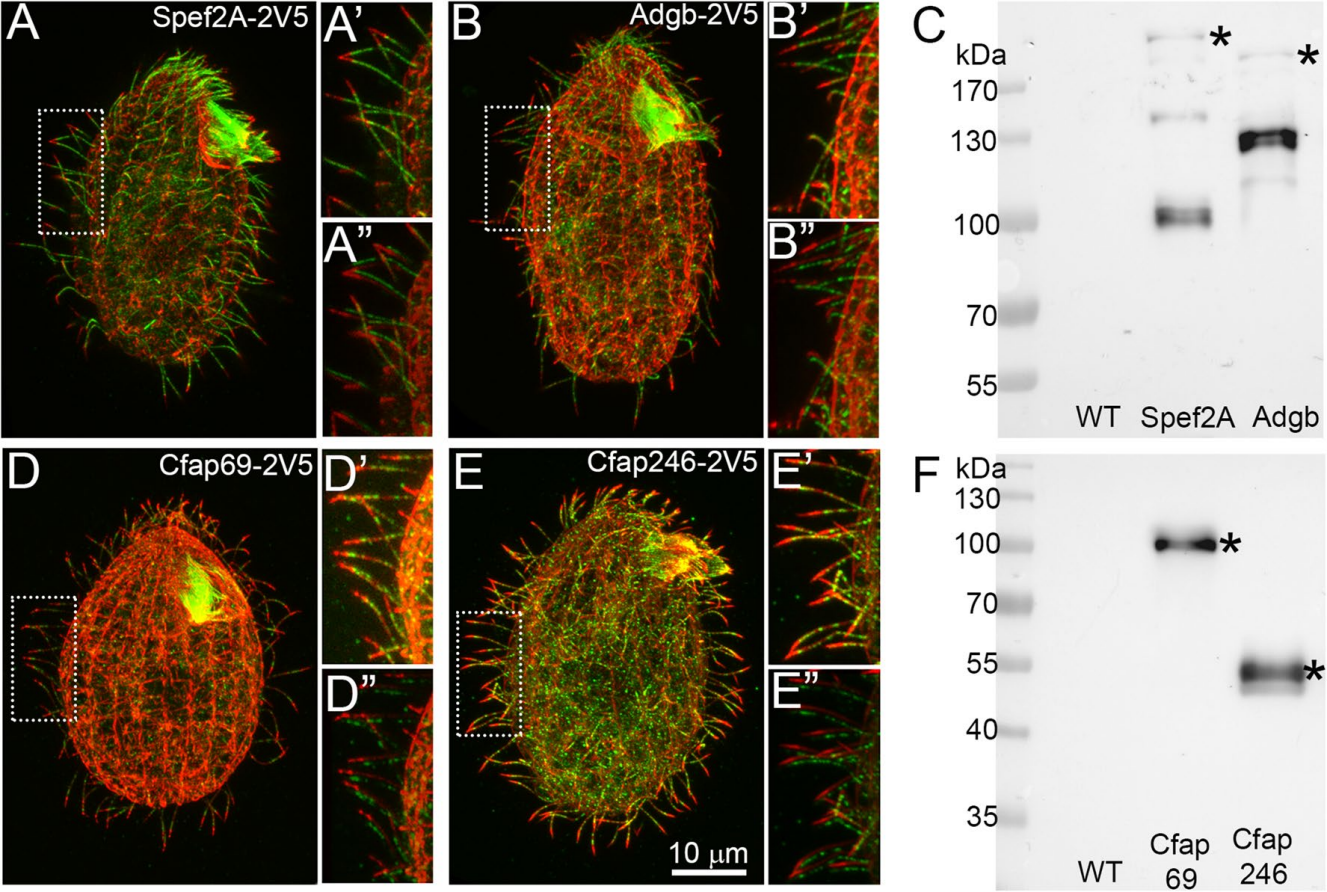
The components of the *Tetrahymena* C1b projection localize throughout the cilium except the cilium tip. (**A-B”**) and (**D–E”**) Merged immunofluorescence confocal images of *Tetrahymena* cells expressing Spef2A-2V5 (**A–A”**), Adgb-2V5 (**B–B”**), Cfap69-2V5 (**D–D”**) or Cfap246-2V5 (**E–E”**) under the control of the respective native promoters, double stained with anti-V5 (green) and anti-α-tubulin (red) antibodies. (**A’**, **B’**, **D’**, **E’**) A magnification of cilia marked with white insets in (**A**, **B**, **D**), and (**E**). (**A”**, **B”**, **D”**, **E”**) The images of the same areas as in (**A’**, **B’**, **D’**) and (**E’**) but the signal from a green channel was slightly shifted in respect to a red channel to better visualize that the ciliary tips are devoid of the fusion proteins. (**C**, **F**) Western blot analyses of 2V5-tagged fusions in cilia isolated from wild-type (WT) and mutant cells expressing: Spef2A-2V5 (**C**), Adgb-2V5 (**C**), Cfap69-2V5 (**F**) or Cfap246-2V5 (**F**) detected using anti-V5 antibodies. Band of the protein migrating according to the predicted molecular mass is marked by an asterisk.

The genome of *Tetrahymena thermophila* encodes two proteins with homology to Spef2/CPC1, here named Spef2A (TTHERM_01142770) and Spef2B (TTHERM_00633390). Both proteins were identified in the *Tetrahymena* ciliome^22^. However, the N-terminal calponin-homology (CH) domain was predicted only in Spef2A (Figs. S1, S2A). Therefore, we assumed that ~ 200 kDa protein, Spef2A, is a true ortholog of mammalian Spef2. When expressed as C-terminal 2V5 or 3HA fusions under control of the native promoter, Spef2A localized in cilia, along their entire length with exception of the distal tip (Fig. 1A-A”).

To identify potential binding partners of Spef2A, we engineered *Tetrahymena* cells expressing Spef2A-HA-BirA* under control of the native promoter and performed proximity labeling assays to identify proteins that are biotinylated within ~ 10 nm^23^. Mass spectrometry analysis of the biotinylated proteins purified from cilia isolated either from the wild-type (negative control) or Spef2A-HA-BirA* expressing cells (Fig. S3A) revealed that Spef2A, Cfap69, Cfap246, Cfap174, Tt170, and Adgb, a protein with limited similarity to androglobin and calpain-7, were repeatedly recovered from cilia of Spef2A-HA-BirA*-expressing cells (Tables 1, S2). None of *Tetrahymena* enolases, HSP70s, or putative FAP39 orthologs were detected among the biotinylated proteins.

**Table 1:** Mass spectrometry-based identification of ciliary proteins biotinylated in cells expressing an indicated BirA*-tagged protein. Table shows the numbers X/Y; (X) number of all identified peptides (in a Mascot program, all significant matches), (Y) number of all unique peptide sequences (in a Mascot program, significant sequences). Complete data are presented in S2-S4 Tables.

Protein name	Number in TGD	WT (control)			Spef2A-BirA*		Cfap246-BirA*		Cfap69-BirA*	
		Exp 1	Exp 2	Exp 3	Exp 1	Exp 2	Exp 1	Exp 2	Exp 2	Exp 3
α-tubulin	TTHERM_00558620	65/14	111/19	31/11	61/14	71/15	60/13	122/20	54/13	37/14
β-tubulin	TTHERM_00348510	93/21	161/22	29/11	67/20	115/21	65/19	199/23	92/15	46/15
Spef2A	TTHERM_01142770	1/1	2/2	0/0	15/11	28/20	236/85	275/90	179/60	28/22
Spef2B	TTHERM_00633390	0/0	0/0	0/0	0/0	0/0	0/0	1/1	0/0	0/0
Cfap69	TTHERM_00691650	0/0	0/0	0/0	7/6	5/4	69/26	73/23	80/25	13/10
Cfap246	TTHERM_00188400	0/0	0/0	0/0	7/7	5/5	68/23	59/20	54/18	5/5
Adgb	TTHERM_00290850	0/0	1/1	0/0	27/21	23/16	249/81	248/82	161/50	24/13
Cfap174	TTHERM_00077420	0/0	1/1	0/0	3/3	1/1	14/5	26/6	13/4	0/0
Tt170	TTHERM_00205170	0/0	0/0	0/0	2/2	4/4	47/14	87/17	58/14	24/10

TTHERM- numbers in Tetrahymena Genome Database (TGD).

Immunofluorescence studies of *Tetrahymena* cells expressing 2V5 fusions of Cfap69, Cfap246, and Adgb (under their native promoter) showed that like Spef2-2V5, these proteins were enriched in cilia except for the distal tip (Fig. 1).

Cfap69 (TTHERM_00691650), a 102 kDa, armadillo motifs-containing protein (Fig. S1) has orthologs in most species assembling motile cilia from protists to humans, with some exceptions (e.g. *Trypanosoma sp.*, *Trichomonas sp., Giardia sp.*, *Drosophila sp.*, and some bonefish lineages including *Danio rerio*) (Fig. S2B).

Cfap246 (TTHERM_00188400) is a 53 kDa, leucine-rich repeats-containing protein, with homology to an N-terminal fragment of *Chlamydomonas* FAP246, and human leucine-rich repeats and guanylate kinase domain-containing protein (LRGUK) (Fig. S2C). In contrast to the much larger human ortholog (94 kDa), *Tetrahymena* Cfap246 protein lacks the guanylate kinase domain within its C-terminal region (Fig. S1).

Cfap174 (TTHERM_00077420), orthologous to *Chlamydomonas* FAP174 and human c-myc binding protein (MYCBP-1), and a ciliate-specific Tt170 (TTHERM_00205170) are small proteins (11 kDa and 18 kDa, respectively) with predicted coiled-coils regions (Figs. S1, S2D).

Androglobin/Adgb (TTHERM_00290850) is a large protein (180 kDa) showing similarity to human androglobin and calpain-7 within the N-terminal region and to androglobin within the short C-terminal fragment (Figs. S1, S2E). Orthologs of Adgb are present in most of the animal lineages^24^.

To verify if identified proteins are indeed located in close proximity to each other, we performed reciprocal BioID experiments by expressing Cfap69, Cfap246, and Adgb as C-terminal HA-BirA* fusions under their native promoters and Adgb with N-terminally positioned BirA*-HA (Figs. S3A,B). As expected Cfap69, Cfap246, Adgb, Spef2A, Cfap174, and Tt170 were enriched among the proteins that were biotinylated in cilia of Cfap69-HA-BirA* and Cfap246-HA-BirA* expressing cells (Tables 1, S3-S5). Although Adgb-HA-BirA* and BirA*-HA-Adgb proteins were targeted to cilia, mass spectrometry failed to detect biotinylated proteins, including Adgb, in experimental samples (even after prolonged 16 h incubation in biotin-enriched medium (Fig. S3A)). Therefore, we attempted to identify potential Adgb interacting proteins using immunoprecipitation. Adgb and Cfap69 proteins were expressed in *Tetrahymena* as fusions with a C-terminal 3HA tag under control of native promoters and interacting ciliary proteins were immunoprecipitated with resin-bound anti-HA antibodies (Fig. S3C). Mass spectrometry of the Adgb-3HA and Cfap69-3HA immunoprecipitates detected Spef2A, Cfap69, Cfap246, and Adgb in both samples while Cfap174 was found only in the Adgb immunoprecipitates (Tables 2, S5). We conclude that Cfap69, Cfap246, Adgb, Cfap174, and Tt170 are positioned near Spef2A, and thus, most likely are subunits of the C1b/C1f projections.

**Table 2:** Mass spectrometry-based identification of the ciliary proteins co-immunoprecipitated with Cfap69-3HA or Adgb-3HA. Table shows the numbers X/Y; (X) number of all identified peptides (in a Mascot program, all significant matches), (Y) number of all unique peptide sequences (in a Mascot program, significant sequences). Data in rows represent data from independent experiments (complete data are presented in S5 Tables).

Protein name	Number in TGD	Number of peptides		
		WT	Cfap69-3HA	Adgb -3HA
Spef2A	TTHERM_01142770	0/0	27/5	83/18
		0/0	34/8	70/23
				31/20
Cfap69	TTHERM_00691650	0/0	16/4	16/5
		0/0	18/4	6/4
				17/13
Cfap246	TTHERM_00188400	0/0	2/1	27/6
		0/0	3/1	16/6
				12/9
Adgb	TTHERM_00290850	0/0	28/7	23/5
		0/0	49/9	38/16
				35/26
Cfap174	TTHERM_00077420	0/0	0/0	8/2
		0/0	0/0	8/4
				6/4
Tt170	TTHERM_00205170	0/0	0/0	0/0
		0/0	0/0	0/0
				4/4

### Cilia of *CFAP69* and *SPEF2A* deletion mutants lack a C1b/C1f projection and exhibit abnormal rotational motion

Next, we engineered *Tetrahymena* strains with deletions of *CFAP69* or *ADGB* using the germ-line-based targeting by homologous DNA recombination (*CFAP69-KO* and *ADGB-KO* cells)^25,26^ and *SPEF2A-coDel*, *ADGB-coDel*, and *CFAP246-coDel* mutants using the co-Deletion method based on the induction of scnRNAs^27^ (Fig. S4). The targeted loci were analyzed by PCR to confirm complete loss of the targeted sequence. We could not recover strains homozygous for the deletion of *CFAP246* (see Material and methods) and therefore we analyzed knockdown of *CFAP246-coDel* strains.

**Figure 2:**
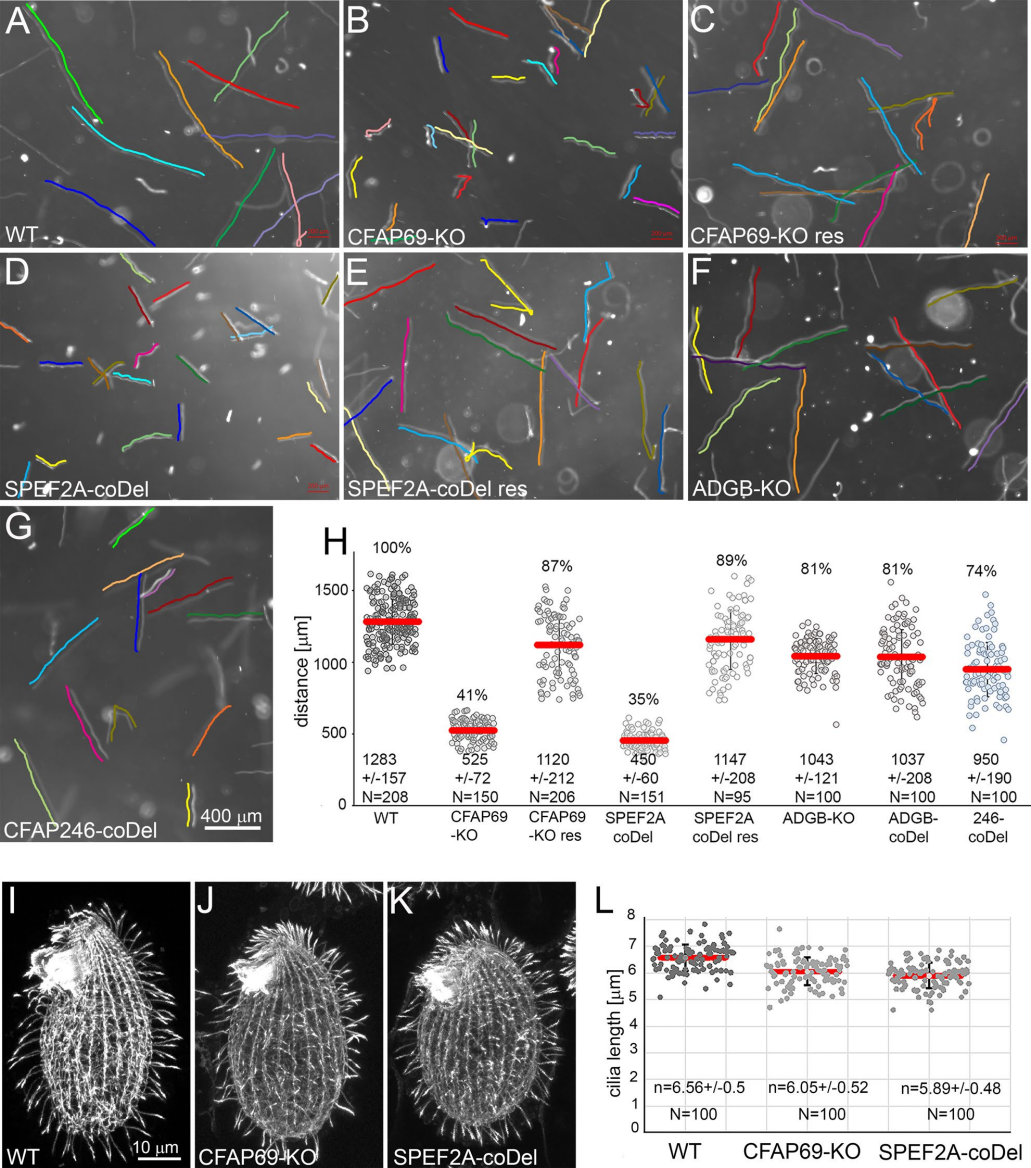
*Tetrahymena* mutant cells with deleted *CFAP69* or *SPEF2A* swim with reduced velocity. (**A–G)** Swimming paths of wild type (**A**) and mutant cells: *CFAP69-KO* (**B**), *CFAP69-KO* rescued (**C**), *SPEF2A-coDel* (**D**), *SPEF2A-coDel* rescued (**E**), *ADGB-KO* (**F**), and *CFAP246-coDel* (**G**). All swimming paths were recorded for 3.2 s at RT using video camera. The cells trajectories are indicated by parallel color lines. Bar = 400 µm. (**H)** Graph showing the comparison of the distances swum by wild-type (WT) and analyzed mutants during 3.2 s. Error bars represent standard deviation. The traveled distance with standard deviation and number of the measured trajectories (N) are indicated at the bottom of the graph. Numbers above the graph indicate % of the distance swum by wild-type cells. Note that ADGB-coDel cells (knockdown) and ADGB-KO cells swam at similar rate. (**I**–**L**) Analysis of the cilia length. (**I**–**K)** Confocal immunofluorescence images of wild-type (**I**) and mutant cells (**J**–**K**) stained with anti-α-tubulin antibodies to visualize cilia. (**L**) Graph showing a length of cilia assembled by analyzed cells. n – mean cilium length with standard deviation, N – number of measured cilia. Observed shortening of cilia in both mutants is statistically significant (*p* < 0.0001, t-test).

A force generated by cilia beating propels *Tetrahymena* cells. The *CFAP69-KO* and *SPEF2A-KO* cells on average traveled only approximately 41% and 35%, respectively, of the distance of the wild-type cells (Fig. 2A,B,D,H). *ADGB-KO* or *ADGB-coDel* mutants and those with Cfap246 knockdown (*CFAP246-coDel*) were less affected and swam at the rate of 81% and 74% of the wild type, respectively (Fig. 2F–H). Reduced swimming rate of *CFAP69-KO* and *SPEF2A-coDel cells*, could be caused either by a reduced number of cilia or changes in their length or altered cilia beating. The immunofluorescence analyses of the wild-type and mutant cells using an anti-α-tubulin antibody showed that the density of cilia in the *CFAP69-KO* and *SPEF2A-coDel* mutants appeared normal but cilia were approximately 8% (6.05 ± 0.52 µm) and 11% (5.89 ± 0.48 µm), respectively, shorter than those assembled by wild-type cells (6.56 ± 0.5 µm) (Fig. 2I–L).

**Figure 3:**
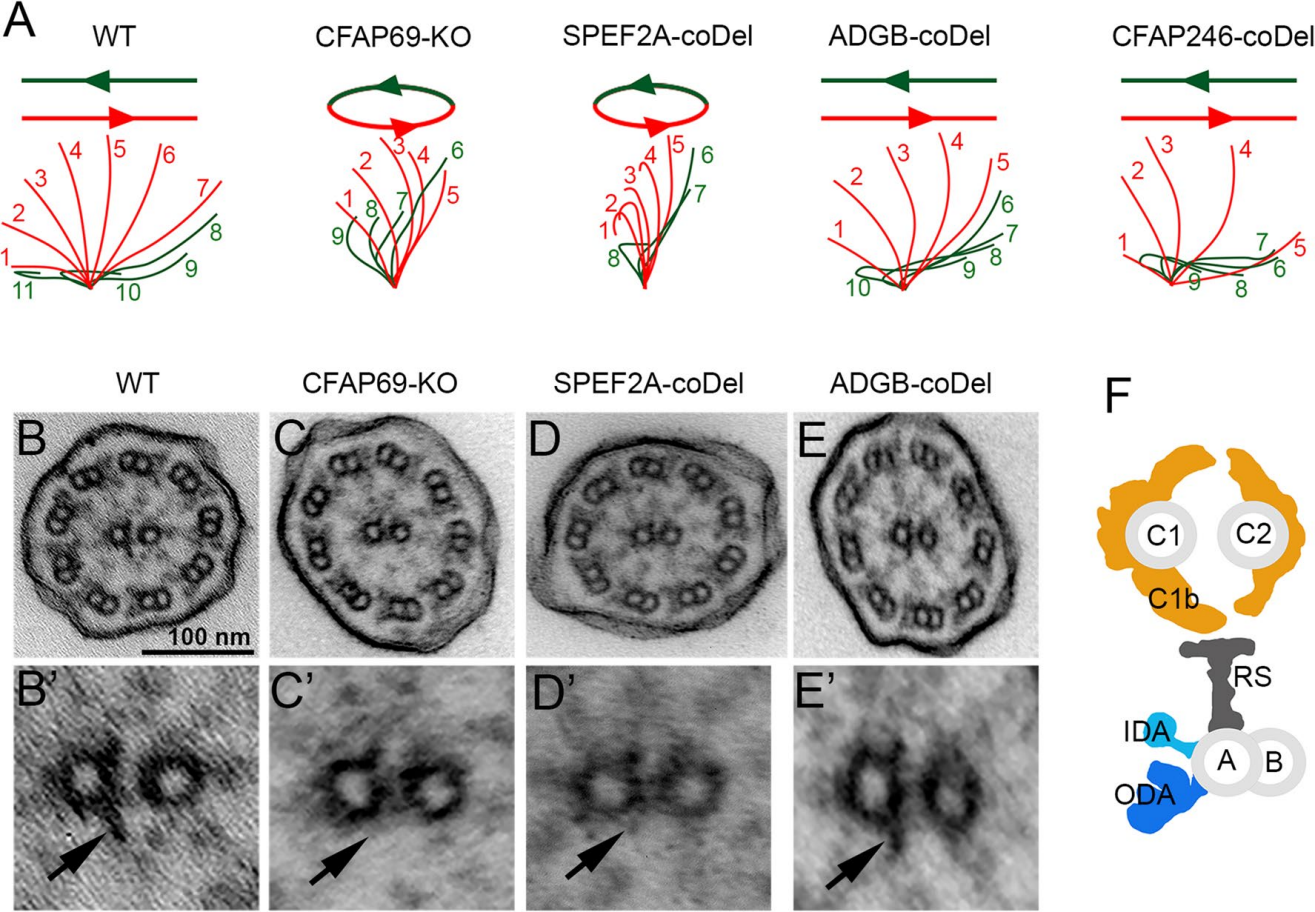
Cilia assembled by *Tetrahymena CFAP69-KO* or *SPEF2A-coDel* mutants lack C1b projection and exhibit a rotatory movement. (**A**) Drawings representing subsequent positions of the cilium during the power (red) and recovery stroke (green) of wild-type and mutant cells prepared based on analyzed movies. (**B–E**) TEM analyses of the ultrastructure of cilia assembled by wild type, *CFAP69-KO*, *SPEF2A-coDel* and *ADGB-coDel* mutants. (**B’–E’)** The magnified images of the CA of cilia shown in (**B–E**). Note a lack of a large C1b projection in *CFAP69-KO* and *SPEF2A-coDel* mutants. (**F**) A drawing showing CA and a selected outer doublet with associated complexes. C1, C2 – microtubules of the CA, C1b – projection missing in analyzed mutants, RS – radial spoke, IDA – inner dynein arm, ODA – outer dynein arm, A, B – tubules of the outer doublet. Drawing was prepared based on cryo-ET images of ciliary structures presented in^2,3^.

Next, we analyzed the motion of cilia using a high-speed video camera (Fig. 3A, Supplementary Movies 1–5). In the wild-type cells cilia beat with two apparent phases, the power and recovery strokes, taking place in different planes. During the power stroke, a tip of the straight cilium follows a semicircle perpendicular to the cell surface. During the recovery stroke, the cilium bends near the base and moves closer to the cell surface to reach the initial pre-power stroke position (Supplementary Movie 1). In contrast to the wild-type cells, cilia of *CFAP69-KO* and *SPEF2A-coDel* mutants exhibited a rotatory motion slightly inclined to the cell surface (Fig. 3A). Moreover, the neighboring mutant cilia frequently collided (Supplementary Movies 2, 3). In the mutants deficient in either Cfap246 or Adgb, the power and recovery strokes were well-defined but the amplitude was slightly reduced and the waveform of the cilium during the recovery phase was slightly altered (Supplementary Movies 4, 5, Fig. 3A). Ectopic expression of HA-Cfap69 in the *CFAP69-KO* background restored cells swimming rate close to the wild-type level (Fig. 2C). Because the coding region of *SPEF2A* is large, we could not perform a similar rescue experiment for the *SPEF2A-coDel* mutants. However, we were able to recover cells with a wild-type motility by replacing the deleted region in the *SPEF2A-coDel* cells with a 3 kb fragment of the wild-type genomic DNA (Fig. 2E). Thus, we conclude that the abnormal ciliary functions in both mutants were due to the deletions at the targeted loci.

In *Tetrahymena* cilia, similar as in *Chlamydomonas* flagella, the CA projections differ in size and shape. Ultrastructural analyses of mutant cilia cross-sections using TEM revealed that the C1b projection was shorter or missing in *CFAP69-KO* and *SPEF2A-coDel* cilia (Figs. 3B-D’,F, S5). Thus, likely both proteins are crucial for either the assembly or stability of the C1b complex. In some *ADGB-coDel* cilia, C1b seemed smaller or twisted (Figs. 3E-E’, S5). In contrast, *CFAP246-coDel* cilia did not have obvious defects. In *Tetrahymena* the C1f projection is not that apparent in TEM cross-sections as in *Chlamydomonas* flagella cross-sections. However, because in *Chlamydomonas cpc1* mutants both C1b and C1f projections are lost, it is possible that also in *Tetrahymena* the entire C1b/C1f region is lost and therefore we will further refer to *CFAP69-KO* and *SPEF2A-coDel* knockouts as C1b/C1f-less mutants.

### The levels of putative C1b/C1f subunits, and IFT and BBS proteins are reduced in cilia assembled by *CFAP69* and *SPEF2A* mutants

The C1b is one of the largest projections of the CA microtubules. Because C1b (and possibly C1f) are either missing or greatly reduced in the *Tetrahymena CFAP69-KO* and *SPEF2A-coDel* mutants, most likely not only the targeted protein but also other components of the C1b/C1f are missing. Moreover, C1b projection may stabilize neighboring projection(s) and thus, loss of C1b could destabilize the C2b (as in *Chlamydomonas*^13,14^) and perhaps C1d projection.

To identify proteins that are either missing or reduced in C1b/C1f-less cilia, we compared the protein composition of cilia of the wild type, *CFAP69-KO,* and *SPEF2A-coDel* mutants by mass spectrometry (six independent samples for each cell type) (Tables 3, S6). MS/MS failed to detect Cfap69 peptides in the *CFAP69-KO* cilia and detected only a single Spef2A peptide in six samples of the *SPEF2A-coDel* cilia, arguing that the obtained mutants lack the targeted proteins. The levels of other putative C1b/C1f supercomplex subunits, Adgb and Cfap246 were strongly reduced (Tables 3, S6). Interestingly, the level of Cfap174 was unaltered.

**Table 3:** Mass spectrometry analysis of the selected proteins in cilia assembled by wild-type, *CFAP69-KO* and *SPEF2A-coDel* mutants.

Protein	TGD acc. no	EMBL acc. no	MW (kDa)	MS/MS qualitative analysis									MS quantitative analysis			
				WT			CFAP69-KO			SPEF2A-coDel			CFAP69-KO/WT		SPEF2A-coDel/WT	
				sum	mean	SD	sum	mean	SD	sum	mean	SD	q-value	fold change	q-value	fold change
**C1b, putative and confirmed**																
Spef2A	TTHERM_01142770	EAR82443.2	203	174	29.0	3.5	108	18.0	2.3	1	0.2	0.4	1.50E-04	- 1.42 ↓	3.00E-05	-3.24 ↓
Cfap69	TTHERM_00691650	EAR84467.2	102	72	12.0	2.1	0	0	0	3	0.5	0.8	3.99E-03	- 3.14 ↓	5.00E-05	-2.29 ↓
Adgb	TTHERM_00290850	EAR98457.3	180	174	29.0	1.5	108	18.0	2.2	2	0.3	0.5	6.30E-04	- 1.36 ↓	8.20E-04	-1.71 ↓
Cfap246	TTHERM_00188400	EAR96271.2	53	57	9.5	1.4	48	8.0	1.2	0	0	0	ns	-	4.60E-04	-2.40 ↓
Tt170	TTHERM_00205170	EAR86859.2	18	61	10.2	1.1	67	11.2	0.9	58	9.7	0.5	1.10E-04	- 1.80 ↓	3.00E-05	-2.11 ↓
Cfap174	TTHERM_00077420	EAR95438.2	11	27	4.5	1.1	25	4.2	1.2	24	4.0	0.6	ns	-	ns	-
ENO1	TTHERM_00486480	EAR85197.1	48	50	8.3	1.1	18	3.0	0.6	24	4.0	1.2	1.60E-04	- 3.92↓	3.00E-05	-3.56 ↓
ENO2	TTHERM_00046480	EAR94453.1	50	13	2.6	1.0	26	4.3	0.5	25	4.2	2.8	ns	-	4.44E-02	2.16 ↑
PYK1	TTHERM_00118600	EAR90515.3	55	65	10.8	2.0	26	4.3	1.1	28	4.7	1.4	8.00E-05	-3.09 ↓	3.00E-05	-3.03 ↓
Hsp70	TTHERM_00105110	EAR92018.1	71	129	21.5	0.5	123	20.5	2.6	111	18.5	1.9	6.80E-04	-1.56 ↓	3.00E-05	-1.87 ↓
**C2b/c/d**																
Hydin	TTHERM_00551040	EAR88946.1	560	506	84.3	4.3	396	66.0	4.2	462	77.0	5.9	8.00E-05	-1.19 ↓	ns	-
Cfap47	TTHERM_000495990	EWS70940.1	361	116	19.3	3.5	44	7.3	2.4	57	9.5	2.5	2.46E-02	-1.40 ↓	1.90E-02	-1.26 ↓
Cfap266	TTHERM_00080020	EAR95597.3	121	70	11.7	1.4	23	3.8	1.8	40	2.7	1.6	Ns	-	3,36E-03	-1,41 ↓
Klp1/Kif9	TTHERM_00502590	EAS02068.1	83	88	14.7	1.7	44	7.3	1.7	81	13.5	1.0	3.00E-05	-2.10 ↓	7.00E-04	-1.46 ↓
**C1d**																
Cfap46	TTHERM_00705200	EAR90704.2	308	118	19.7	2.4	92	15.3	1.4	117	19.5	2.7	ns	-	ns	-
Cfap54	TTHERM_00049190	EAR94374.2	426	183	30.5	2.7	120	20.0	4.7	137	22.8	3.7	ns	-	ns	-
Cfap74	TTHERM_00530270	EAR85075.2	133	87	14.5	1.7	76	12.7	1.4	84	14.0	1.5	ns	-	ns	-
Cfap221	TTHERM_00189530	EAR96384.3	104	60	10.0	1.4	51	8.5	1.8	65	10.8	1.1	ns	-	ns	-
**Ciliary transport – IFTA**																
IFT43	TTHERM_00202900	EAR86830.2	17	12	2.0	1.0	3	0.5	0.5	8	1.3	1.1	8.61E-02	-1.99↓	5.68E-02	-2.90↓
IFT121/FAP118	TTHERM_00261950	EAR88798.2	140	191	31.8	2.2	79	13.2	2.0	130	21.7	2.0	3.00E-05	-2.40 ↓	3.00E-05	-1.92↓
IFT122	TTHERM_00694540	EAS03374.2	144	120	20.0	3.3	43	7.2	1.6	75	12.5	1.0	3.00E-05	-2.58 ↓	3.00E-05	-1.98 ↓
IFT139/FAP60	TTHERM_00219470	EAS00374.2	154	231	38.5	2.6	91	15.2	4.4	142	23.7	3.0	3.00E-05	-2.88 ↓	3.00E-05	-2.03 ↓
IFT140	TTHERM_00220810	EAS00408.1	161	156	26.0	2.3	83	13.8	0.9	105	17.5	3.4	3.00E-05	-2.60 ↓	3.00E-05	-1.83 ↓
IFT144/FAP66	TTHERM_01093530	EAR82687.2	158	183	30.5	4.1	75	12.5	1.3	120	20.0	2.2	3.00E-05	-2.31 ↓	3.00E-05	-1.91 ↓
**Ciliary transport—IFTB**																
IFT20	TTHERM_00334500	EAR97273.1	15	24	4.0	1.0	19	3.2	0.7	16	2.7	0.5	ns	-	ns	-
IFT38	TTHERM_01474510	EAR81839.1	46	61	10.2	0.9	50	8.3	2.3	50	8.3	0.9	ns	-	ns	-
IFT46	TTHERM_00193580	EAR96866.2	40	48	8.0	0.6	32	5.3	0.7	39	6.5	1.5	2.47E-02	-1.91 ↓	1.81E-02	-1.55 ↓
IFT52	TTHERM_00648910	EAR84622.3	49	85	14.2	1.3	65	10.8	2.3	76	12.7	1.1	1.80E-04	-1.85 ↓	4.90E-04	-1.45 ↓
IFT54	TTHERM_01070330	EAS06586.1	50	76	12.7	1.2	48	8.0	1.4	62	10.3	0.7	3.00E-05	-2.25 ↓	3.00E-05	-1.66 ↓
IFT57	TTHERM_01298520	EAR82129.3	47	6	1.0	0.8	23	3.8	1.5	2	1.0	0.0	9.93E-03	-1.75 ↓	3.81E-03	-1.48 ↓
IFT74	TTHERM_00149230	EAS01313.2	68	102	17.0	1.5	88	14.7	1.8	105	17.5	2.2	3.00E-05	-1.67 ↓	3.00E-05	-1,69 ↓
IFT80	TTHERM_01084200	EAR82753.3	86	135	22.5	2.9	88	14.7	2.6	106	17.7	1.7	3.00E-05	-1.79 ↓	3.00E-05	-1.49 ↓
IFT81	TTHERM_01013160	EAR85980.1	84	124	20.7	2.0	84	14.0	2.8	101	16.8	1.2	3.00E-05	-1.76 ↓	3.00E-05	-1.55 ↓
IFT88	TTHERM_01142720	EAR82438.2	85	89	14.8	0.7	65	10.8	1.8	77	12.8	1.3	1.10E-04	-1.80 ↓	1.00E-04	-1.58 ↓
IFT172	TTHERM_00089240	EAR92529.2	205	285	47.5	3.5	197	32.8	5.3	246	41.0	3.0	3.00E-05	-1.70 ↓	3.00E-05	-1.51 ↓
**BBSome**																
BBS1	TTHERM_01084190	EAR82752.1	64	68	11.3	2.0	6	1.0	1.0	25	4.2	1.1	3.60E-04	-2.69 ↓	4.50E-04	-2.66 ↓
BBS2	TTHERM_00463860	EAR98638.2	81	64	10.7	1.1	15	2.5	1.1	33	5.5	1.7	2.36E-03	-1.88 ↓	4.60E-04	-2.02 ↓
BBS4	TTHERM_01054280	EAR82943.2	49	37	6.2	1.9		0.0	0.0	5	0.8	0.7	ns	-	1.48E-02	-2.01 ↓
BBS5	TTHERM_00782070	EAR91218.2	41	19	3.2	0.7	5	0.8	0.7	8	1.3	0.5	ns	-	ns	-
BBS7	TTHERM_00655860	EAR84554.2	84	80	13.3	1.4	18	3.0	1.0	36	6.0	1.8	3.58E-03	-3.00 ↓	1.13E-03	-2.44 ↓
BBS8	TTHERM_000578529	EWS72509.1	58	23	3.8	1.1	1	0.2	0.4	7	1.2	0.7	1.46E-02	-3.25 ↓	3.37E-02	-1.90 ↓
BBS9	TTHERM_00518740	EAR95042.1	95	53	8.8	2.1	10	1.7	0.7	22	3.7	1.2	1.36E-03	-2.55 ↓	4.40E-04	-2.74 ↓
**Other**																
Spef2B	TTHERM_00633390	EAR89832.2	194	68	11.3	2.2	42	7.0	1.3	78	13.0	1.2	ns	-	ns	-
ATU1	TTHERM_00558620	EAS02179.1	50	206	34.3	1.6	183	30.5	1.7	202	33.7	1.1	ns	-	ns	-
BTU1	TTHERM_00836580	EAS04986.1	50	266	44.3	0.9	281	46.8	1.7	282	47.0	1.0	ns	-	ns	-

Qualitative MS/MS analysis: sum represents all identified peptides in six analyzed samples, mean – mean number of peptides per sample, SD – standard deviation. Quantitative MS analysis: q-value—FDR corrected p-value for Diffprot. Abbreviations: ENO – enolase, PYK – pyruvate kinase, IFT – intraflagellar transport, BBS – Barded-Biedl syndrome, ATU1 – α-tubulin, BTU1 – β-tubulin.

In *Chlamydomonas*, enolase and HSP70 were identified as components of the CPC1 complex^13,14,21^. The genome of *Tetrahymena* encodes three enolases, Eno1 (TTHERM_00486480), Eno2 (TTHERM_00046480), and Eno3 (TTHERM_ 00,474,960). Eno1 and Eno2 were detected in wild-type cilia. In the *CFAP69-KO* and *SPEF2A-coDel* cilia, the levels of Eno1 were reduced, but surprisingly, the level of Eno2 was higher in *SPEF2A-coDel* than in the wild-type cilia (Tables 3, S6). Interestingly, besides enolase, also the level of a pyruvate kinase (PYK1), another enzyme of the glycolytic pathway, was reproducibly lower in mutant cilia.

Out of 14 members of the HSP70/DnaK family, only three were found repeatedly in the wild-type ciliome: Hsp70 (TTHERM_00105110), Ssa6 (TTHERM_00171850), and Hsp110/DnaK (TTHERM_00688300). The level of Hsp70 was slightly decreased in the mutant cilia (Tables 3, S6).

Based on the ultrastructural alterations in the *Chlamydomonas cpc1* flagella, C1b may stabilize C2b^13,14^. The levels of hydin^7^ and putative C2b subunits, Cfap47 and Cfap266^19,20^ were reduced in the C1b/C1f-deficient *Tetrahymena* cilia. Additionally, we detected a reduction of Klp/Kif9, a potential subunit of *Chlamydomonas* C2c/C2d complexes^19,20,28^ (Table 3). The levels of the C1d subunits remained unchanged in the mutant cilia (Table 3). Similar, the level of the CH domain-less Cpc1/Spef2 ortholog, Spef2B was not significantly changed.

Interestingly, the levels of the proteins that mediate intraciliary transport were either substantially (IFT-A and BBS proteins) or moderately (IFT-B proteins) reduced in the C1b/C1f-deficient cilia (Table 3). These data correlate with the slightly reduced length of cilia in *CFAP69-KO* and *SPEF2A-coDel* mutants.

### Level of C1b/C1f proteins is mainly regulated within cytoplasm

**Figure 4:**
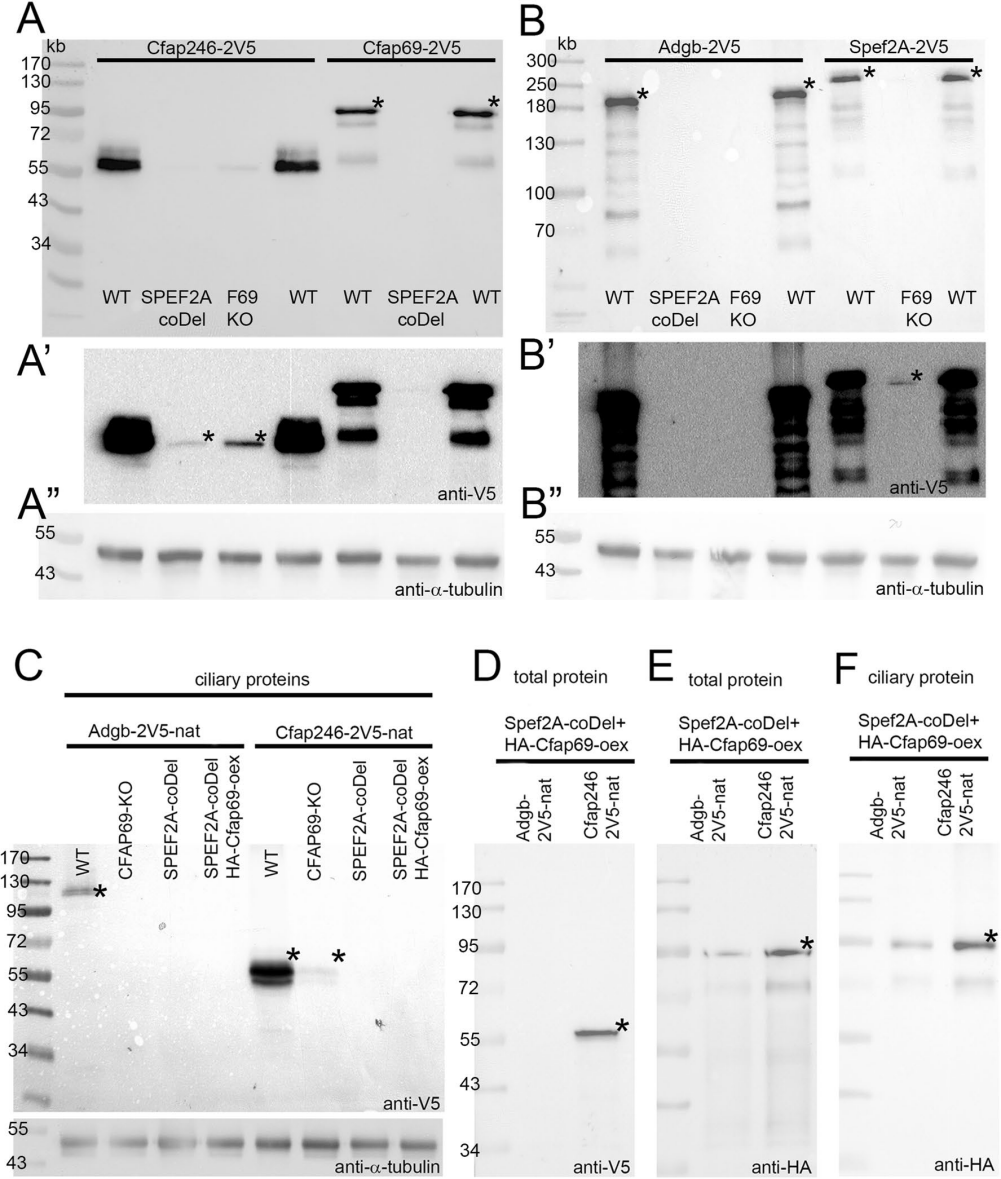
Lack of Spef2A or Cfap69 affects levels of other C1b/C1f subunits in cilia and total cell extract. The western blot analyses of the level of V5-tagged C1b/C1f proteins in total cell extract (**A**, **B**) and ciliary fraction (**C**). (**A’**, **B’**) the same blots as in (**A, B**) but after longer exposure. (**A”**, **B”**) corresponding blot stained with anti-α-tubulin antibodies (loading control). (**D**-**F)** Overexpressed HA-Cfap69 is targeted to cilia (**F**) in *SPEF2A-coDel* mutant and partly stabilizes Cfap246-2V5 but not Adgb-2V5 (**D**). * indicates protein migrating according to the predicted molecular mass in the case of more than one band due to some protein degradation or weakly detected protein (A’, B’, C’).

Lack or reduced levels of C1b/C1f subunits in *CFAP69-KO* or *SPEF2A-coDel* cilia (Fig. 4C) can be explained either by their inability to stably dock to the central microtubules in the absence of Spef2A or Cfap69, or by the reduction of the total amounts of C1b/C1f subunits in mutant cells. Study in *Chlamydomonas* showed that the level of mRNAs for proteins that form the same ciliary complex can be co-regulated^29^.

We quantified the levels of *SPEF2A*, *CFAP69*, *CFAP246,* and *ADGB* mRNAs in the wild-type and *CFAP69* and *SPEF2A* knockouts. qRT-PCR revealed that the levels of *CFAP246* and *ADGB* mRNAs were basically unaffected in mutants (Fig. S6A). Similarly, the levels of *CFAP69* mRNA in *SPEF2A-coDel* and *SPEF2A* mRNA in *CFAP69-KO* cells were similar to that of the wild type. As expected, the *CFAP69* mRNA was undetectable in *CFAP69-KO* cells. Surprisingly, a prominent amount of the *SPEF2A* transcript was present in the *SPEF2A-coDel* cells, suggesting that a transcript was produced at the locus carrying the deletion. Thus, the expression levels of mRNAs for the individual C1b/C1f components are not affected by the losses of other subunits. Therefore, the reduced levels of the C1b/C1f subunits in C1b/C1f-deficient cilia are either due to reduced protein synthesis, decreased transport into the cilia, or increased protein degradation. Therefore, next we assessed the total levels of C1b/C1f proteins in *CFAP69-KO* and *SPEF2A-coDel* cells. Because the antibodies against *Tetrahymena* CA proteins are not available, we engineered strains that express 2V5-tagged fusions of Cfap69, Spef2A, Cfap246, or Adgb each under the control of the respective native promoters. The transgenes were incorporated into the macronuclear genome in wild-type, *CFAP69-KO*, or *SPEF2A-coDel* genetic backgrounds.

The macronuclear genome of *Tetrahymena* contains ~ 45 copies for each protein-coding gene. Initially, the biolistically introduced transgenes incorporate into one to few loci and the ratio of the transgene to endogenous alleles increases with increasing the selection pressure during cell multiplication (so-called phenotypic assortment, see Materials and Methods). Using qPCR and genomic DNA as a template we confirmed that similar number of transgene copies were assorted in all cell strains (Fig. S6B). A western blots analysis of the total cell extracts revealed that the non-targeted C1b/C1f subunits were undetectable or greatly reduced in the C1b/C1f knockouts (Fig. 4A,B). Thus, in the absence of either Cfap69 or Spef2A, other subunits of the C1b/C1f complex may be more prone to proteolytic degradation.

The longevity of a ciliary protein within the cell body may depend upon the presence of partner proteins that stabilize the complex^22^. Therefore, we overexpressed HA-Cfap69 (using cadmium-inducible promoter)^30^ in the *SPEF2A-coDel* cells expressing either Cfap246-2V5 or Adgb-2V5 (under native promoters). The overproduced HA-Cfap69 was targeted to cilia (Fig. 4E,F). Despite this, the Adgb-2V5 was still undetectable while Cfap246-2V5 was stabilized within the cell body (Fig. 4D) but not targeted to cilia (Fig. 4C). Thus, likely (1) Cfap69 stabilizes Cfap246 but not Adgb and (2) within C1b/C1f complex Cfap69 likely binds to Cfap246.

### The expression of Rsp4/6A-GFP or Rsp4/6C-GFP does not rescue *CFAP69-KO* and *SPEF2A-coDel* mutant cell motility

The *pf6*, an immotile *Chlamydomonas* mutant lacking the C1a projection^9,13^ can partly regain motility when one of the radial spoke proteins (RSP3, RSP4 or RSP6) is extended by a C-terminal epitope tag^16^. RSP3 forms a part of the radial spoke stem while the paralogous RSP4 and RSP6^31^ are components of the radial spoke head^32^. Their C-termini are located on or above the radial spoke head upper surface that temporarily comes in contact with the CA projection(s)^16^. In contrast, the artificial expression of RSP4 did not rescue *Chlamydomonas cpc1* mutant lacking C1b/C1f complex (Table S1 in^16^).

**Figure 5:**
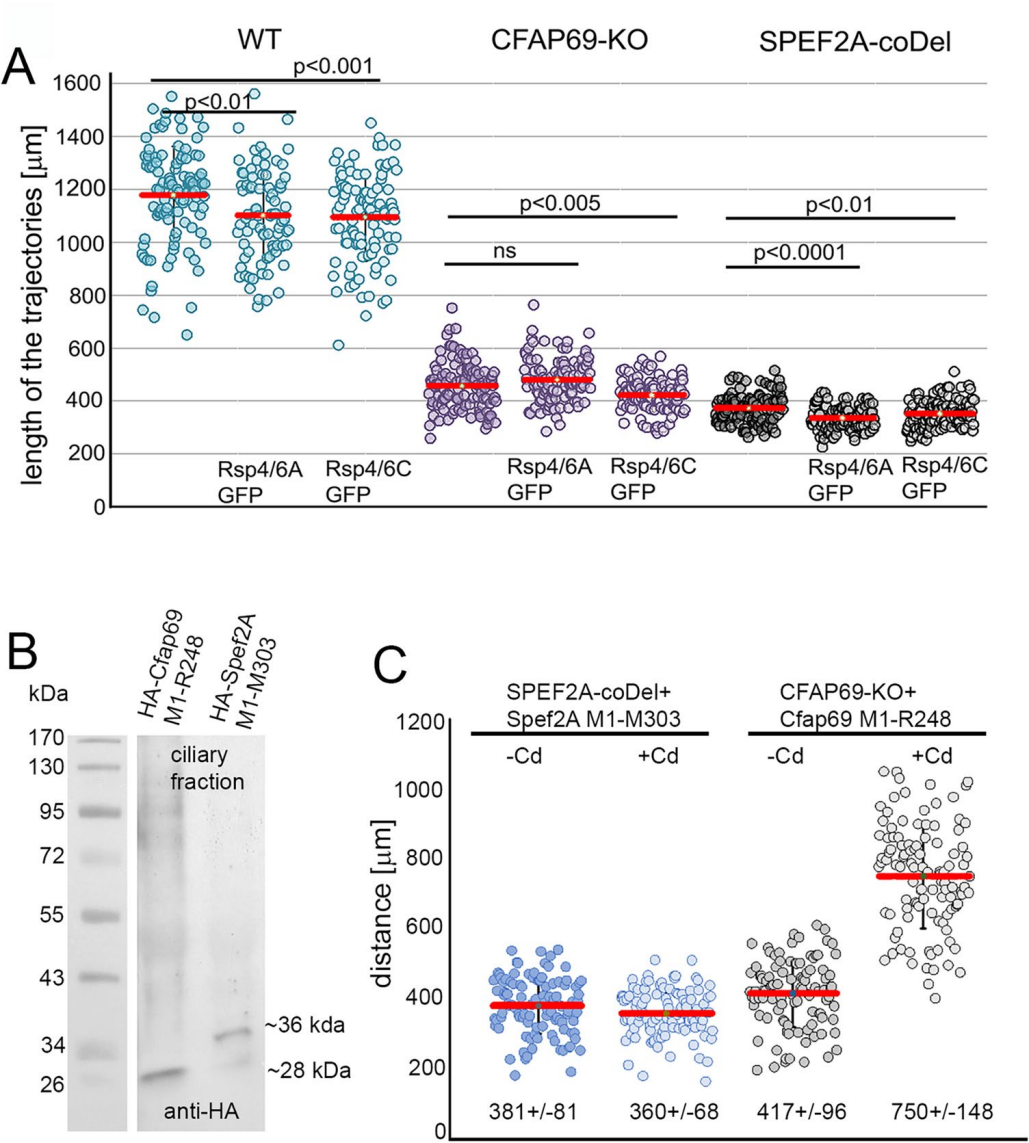
Overexpression of the truncated variant of Cfap69 but not expression of the GFP-tagged radial spoke head proteins partly restores *CFAP69-KO* mutant cells motility. (**A**) Graph showing the comparison of the distances swum by wild-type (WT) and analyzed mutants during 3.2 s, expressing either Rsp4/6A-GFP or Rsp4/6C-GFP under the control of the respective native promoters. Error bars represent standard deviation (SD). Travelled distances (in µm) with SD and number of analyzed trajectories (N): WT: 1178 ± 185, N = 100; WT + Rsp4/6A-GFP: 1102 ± 170, N = 81, WT_Rsp4/6C-GFP: 1094 ± 166, N = 100, CFAP69-KO: 458 ± 91, N = 106, CFAP69-KO + Rsp4/6A: 479 ± 85, N = 104, CFAP69-KO + Rsp4/6C: 422 ± 64, N = 102, SPEF2A-coDel: 372 ± 56, N = 106, SPEF2A-coDel + Rsp4/6A:335 ± 45, N = 100, SPEF2A-coDel + Rsp4/6C: 352 ± 53, N = 106. *p* values calculated using t-test. (**B**) The western blot analysis of the level of expression of HA-Cfap69 M1-R248 and HA-Spef2A M1-M303 truncations in cilia of *CFAP69-KO* and *SPEF2A-coDel* cells respectively. (**C**) Graph showing the comparison of the distances swum during 3.2 s by *SPEF2A-coDel* cells with introduced transgene enabling expression of the HA-Spef2A M1-M303 truncation and *CFAP69-KO* cells with introduced transgene enabling expression of the HA-Cfap69 M1-R248 truncation, either non-induced (−Cd) or induced (+ Cd) to overexpressed truncated protein. Error bars represent standard deviation. Numbers below the graph indicate the distance swum by the analyzed cells. Number of measurements: N = 103, N = 107, N = 102, N = 107, respectively. Difference observed in FAP69-KO + Cfap69 M1-R248 −/ + Cd is statistically significant (*p* < 0.01, t-test).

To further explore the role of C1b in the signal transduction in *Tetrahymena*, we performed similar experiments. The *Tetrahymena* genome encodes three proteins orthologous to RSP4/RSP6: Rsp4/6A (TTHERM_00427590), Rsp4/6B (TTHERM_00502580) and Rsp4/6C (TTHERM_00444180). According to the *Tetrahymena* Functional Genomics Database (http://tfgd.ihb.ac.cn/), *Rsp4/6A* and *Rsp4/6C* are expressed at higher levels than *Rsp4/6B*. Thus, we expressed either Rsp4/6A-GFP or Rsp4/6C-GFP (under the control of their native promoters) in wild-type cells, *SPEF2A-coDel,* or *CFAP69-KO* mutants (Fig. S7). The expression of the GFP-tagged Rsp4/6 proteins did not change considerably cell swimming rate (Fig. 5A).

Next, we investigated if the elevated level of Rsp4/6A-GFP or Rsp4/6C-GFP affects cells motility. The swimming velocity of the wild-type cells grown for 16–18 h in the medium with cadmium (2.5 µg/ml) was reduces by approximately 15% (WT-Cd, 1103 ± 176, n = 51, WT + Cd, 935 ± 176, n = 53). When Rsp4/6A-GFP or Rsp4/6C-GFP were overexpressed (cells were grown in a culture medium with cadmium, Fig. S8A) the swimming velocity of the otherwise wild-type cells was reduced by 27% (Rsp4/6A-GFP) or 31% (Rsp4/6C-GFP) while overexpression of the radial spoke proteins in *CFAP69-KO* and *SPEF2A-coDel* mutants did not change or slightly reduced cells motility (Figs. S8B-J, S9). Thus, similar as in *cpc1* mutant, the expression of the Rsp4/6-GFP did not improve mutant cells’ motility, suggesting a different mechanism of the signal transduction between radial spokes and C1a or C1b projections.

### Cfap69 truncation corresponding to human MMAF-causing CFAP69 pGln255X variant partly rescues *Tetrahymena* cell motility

In humans, mutations in several genes encoding components of the CA cause primary ciliary dyskinesia (PCD) or more frequently, multiple morphological abnormalities of the sperm flagella (MMAF) syndrome^33,34^. Recent genetic analysis using whole-exome sequencing identified homozygous stop-gain mutation in *CFAP69* (p.Gln255X^35^) and *SPEF2* (p.Arg304X^36^) as likely MMAF causal mutations. To better understand the outcome of these mutations, we investigated the effect of corresponding mutations in *CFAP69* (Ser249X) and *SPEF2A* (Lys304X) on *Tetrahymena* cells motility. We expressed HA-Cfap69-M1-R248 protein fragment in the *Tetrahymena CFAP69-KO* and HA-Spef2A-M1-M303 fragment (containing the CH domain) in the *SPEF2A-coDel* background, both under *MTT1* promoter. Western blots showed that when overexpressed both truncated proteins were present in cilia (Fig. 5B). Interestingly, expression of HA-Cfap69-M1-R248 partially rescued the slow motility phenotype of the *CFAP69-KO* cells, on average to the level of 80% of the wild-type (Fig. 5C). Spef2A truncation did not have such a rescuing effect (Fig. 5C). We conclude that disease-related truncated variants of Cfap69 and Spef2A can be targeted to cilia and thus the targeting determinants are present within the remaining N-terminal domains of these proteins. Furthermore, among the variants that cause MMAF, Gln255X Cfap69 could be partially functional while the Arg304X Spef2 is likely severely functionally compromised.

## Discussion

In contrast to outer doublet components, the composition of the CA is poorly characterized. Surprisingly, although the overall CA architecture is similar in cilia/flagella of diverse species^3^, approximately 60% of the identified *Chlamydomonas* CA proteins lack obvious orthologs in other ciliated eukaryotes including humans^19,20^. These observations raise two questions. First, what is the protein composition of the CA in cilia and flagella assembled by other species and specifically, are there additional conserved components in other lineages? Second, since patterns of cilia/flagella beating vary in different species, do lineage-specific CA components contribute to the regulation of the beating patterns?

We have shown that evolutionarily conserved Spef2A, Cfap69, Cfap246, Adgb, and *Tetrahymena*-specific Tt170 are likely structural components of the C1b/C1f supercomplex in *Tetrahymena* and that lack of the entire C1b/C1f complex changes cilium motion from two-phase to rotatory. Besides Tt170 and Adgb, orthologs of other putative C1b/C1f proteins were also suggested to build C1b/C1f structure in *Chlamydomonas*^13,14,19,20^. Information concerning localization and function of the novel putative C1b/C1f components are limited. Some data link these proteins with cilia/flagella in mammals. In human and mice sperm flagella, CFAP69 is present in the midpiece and its truncation results in either mislocalization or loss of SPEF2^35^. This agrees with our data showing that Spef2A is reduced in the *CFAP69-KO* cilia. Mutations in *CFAP69* cause male infertility^35,37^ but the connection between CFAP69 and ultrastructural changes in sperm cells is unclear.

Interestingly, in mice, CFAP69 is also present in the immotile olfactory cilia of the olfactory sensory neurons (OSN)^38^. The OSN cilia can be divided into two segments, proximal containing a CA (9 × 2 + 2) and distal with a decreasing number of microtubules (from 9 × 1 to 4 × 1)^39^. It will be of interest to determine whether in the OSN cilia CFAP69 is present only in the CA-containing proximal fragment or along the entire cilia length, suggesting another intraciliary localization and likely function in these sensory cilia.

Cfap246 orthologs contain LRR domains in their N-termini. The LRR domains form a horseshoe shape which provides a scaffold for protein–protein interactions^40^. In contrast to *Tetrahymena* Cfap246, *Chlamydomonas* FAP246/CHLRE_14g618750v5 and the mammalian LRGUK are much larger proteins. LRGUK-1, besides LRR domains, also has a guanylate kinase domain while predicted FAP246/ CHLRE_14g618750v5 contains C-terminal EF-hand domains. Thus, Cfap246 orthologs could mediate protein–protein interactions (LRR) and have lineage-specific functions that involve Ca^2+^ signaling and local regulation of GMP, and indirectly, cGMP.

In humans, LRGUK is highly enriched in the trachea, testis (GDS3113/190,191; https://www.ncbi.nlm.nih.gov/geoprofiles) and spermatozoa and in mouse Lrguk-1 localizes to the acrosome and the sperm tail^41,42^. *LRGUK-1* mutant mice either lack the sperm tail or assemble a short one^41^. It remains to be determined if LRGUK is indeed a C1b/C1f component in mammals.

In *Chlamydomonas,* FAP174 binds to an unidentified protein tentatively named AKAP240 (A-kinase anchoring protein)^43^ suggested to be a component of the C2 region of the CA^44^. On the other hand, FAP174 co-immunoprecipitates with FAP246^19^. This latter result agrees with our data showing that Cfap174 is positioned near Cfap246. Surprisingly, the level of Cfap174 was unaltered in cilia of *Tetrahymena SPEF2A-coDel* or *CFAP69-KO* mutants but slightly reduced in *Chlamydomonas cpc1* mutant^20^. Considering that C2b projection is partly or entirely missing in flagella of the *Chlamydomonas cpc1* mutant, we speculate that FAP174/Cfap174 is positioned at the very distal end of C1b or C2b projection. This model is supported by following data: (1) Cfap174 co-immunoprecipitates with Adgb but not with Cfap69 which together with Spef2A likely docks the projection to the C1 microtubule, (2) the distal part of C1b but not the entire projection is altered in the Adgb knockdown cells, suggesting that Adgb forms a distal part of C1b, (3) Cfap174 is highly biotinylated in cells expressing Cfap246-HA-BirA* but not in cells expressing Spef2A-HA-BirA*, and thus likely positioned in close proximity to Cfap246. Alternatively, Cfap174 may have multiple axonemal docking sites as it was suggested for *Chlamydomonas*^20^.

Orthologs of *Chlamydomonas* FAP42 were found only in unicellular green algae. A detailed inspection of the FAP42 and Adgb sequences shows that both proteins contain a calpain-like domain near their N-termini. Thus, we speculate that the Adgb and FAP42 have similar position within C1b/C1f supercomplex. Importantly, in mammals androglobin is expressed at higher level in cells assembling motile cilia and its expression is co-regulated by FOXJ1^24,45^.

In contrast to what has been observed in *Chlamydomonas*^21^, in *Tetrahymena* neither enolases nor Hsp70 were detected among proteins associated with C1b/C1f subunits (did not specifically co-immunoprecipitated with C1b/C1f subunits or were biotinylated in cells expressing BirA*-tagged C1b/C1f proteins, Tables S2-5). The genome of *Tetrahymena* encodes several proteins with similarity to Hsp70 and three enolases. Interestingly, the level of Hsp70 encoded by TTHERM_00105110 and Eno1 was reduced in *FAP69-KO* and *SPEF2A-coDel* mutants lacking C1b/C1f, suggesting that structural C1b/C1f proteins could transiently serve as a scaffold for Hsp70 and Eno1 docking or that Hsp70 and Eno1 loosely attach to the C1b/C1f projections. Enolases catalyze the penultimate step of glycolysis i.e., the conversion of 2-phosphoglycerate to phosphoenolpyruvate. Interestingly, in the C1b/C1f-deficient cells also the level of the pyruvate kinase, an enzyme that catalyzes a transfer of the phosphate group from phosphoenolpyruvate to ADP yielding synthesis of ATP, is significantly reduced. Thus, likely as was earlier shown in *Chlamydomonas*^21^, C1b/C1f function as a scaffold for enzymes that locally regulate ATP level.

The predicted molecular mass of FAP39/CHLRE_02g145100v5, a P-type ATPase, is 127 kDa that is close to the weight of the unidentified protein that co-sediments with CPC1/SPEF2^13^. The *Tetrahymena* genome encodes several P-type ATPases and three of them were found in the *Tetrahymena* ciliary proteome (Table S6). One of these three proteins (TTHERM_00522430) was reduced in the C1b/C1f-deficient cilia (Tables S6). However, we did not identify FAP39 orthologs in BioID and co-IP assays. Moreover, both *Chlamydomonas* FAP39/CHLRE_02g145100v5 and three ciliary *Tetrahymena* Cfap39 orthologs likely contain transmembrane domains (as predicted http://www.cbs.dtu.dk/services/TMHMM/) that makes their presence in the CA structure unlikely.

The bioinformatics search using *Tetrahymena* and human genome databases did not reveal any orthologs of *Chlamydomonas* CHLREDRAFT_177061 or CHLREDRAFT_170023. The presence of the WD40 repeats in the latter rendered limited similarity to WD40-containing protein, FAP57. However, the *Tetrahymena* protein identified with the highest score in the blastp search was not detected as biotinylated in Spef2A-, Cfap69- or Cfap246-HA-BirA* expressing cells (Tables S2-5).

To sum up, we propose that Spef2A, Cfap69, Cfap246, Adgb, and Tt170 form a scaffold of the *Tetrahymena* C1b/C1f projections, Cfap174 is positioned at the distal end of C1b or C2b, while the enolase Eno1 and Hsp70, are either loosely or transiently attached to C1b/C1f projections.

*Tetrahymena SPEF2A-coDel* and *CFAP69-KO* cells assemble cilia that are slightly shorter than that in wild-type cells. Interestingly, the levels of IFT and BBS proteins are reduced in those mutants. The BBSome interacts with IFT and mediate transport of the ciliary membrane proteins^46^. The lower level of intraflagellar transport proteins could account for the reduced length of mutant cilia.

The levels of IFT and BBS proteins were not investigated in *Chlamydomonas cpc1* mutant but the length of mutant flagella was similar as in wild types^13,14^. Some *Chlamydomonas* CA mutants assemble short flagella. However, in the CP-less short flagella assembled by *Chlamydomonas pf15* (katanin p80), *pf18* or *pf19* (katanin p60) mutants, the levels of the IFT and BBS proteins was significantly^19,47^ or slightly^20^ elevated compared to wild-type flagella, and IFT proteins and BBS4 were trapped in the lumen of the CA-less, detergent extracted axonemes^47^. The amount of the trapped IFT proteins was reduced when the CA re-assembled^47^. Thus, the reduced level of IFT and BBs proteins in C1b/C1f-less *Tetrahymena* cilia is an unexpected result. It would be interesting to investigate if the lack of other CA projections also affects the level of IFT and BBS proteins and cilia length in *Tetrahymena*.

Interestingly, in mice, the IFT20 was identified as a Spef2 partner protein in maturing sperm cells^48^. In mammals, the differentiation of spermatids requires an intense transport of cargoes along the manchette microtubules and this process involves intraflagellar transport proteins^49^. However, how Spef2 is related to intraflagellar and IFT-related inter-manchette transport is to be determined.

## Materials and methods

### *Tetrahymena* strains and culture

The wild-type CU428.2 and B2086.2 *Tetrahymena* strains were obtained from the *Tetrahymena* Stock Center (Cornell University, Ithaca, NY, USA). Wild-type and other motile strains were grown in the SPP (Super Proteose Peptone) medium^50^ with the antibiotic–antimycotic mix at 1:100 (Sigma-Aldrich, St-Louis, MO, USA). Mutants with greatly reduced motility were grown in MEPP (Modified Enriched Proteose Peptone) medium, on which cells take up nutrients without using oral cilia^51^, supplemented with the antibiotic–antimycotic mix at 1:30 (Sigma-Aldrich, St-Louis, MO, USA). Cells were grown with moderate shaking (80 rpm) at 30 °C. Cell swimming and cilia beating patterns were analyzed as previously described^52,53^. To induce protein overexpression, cells were cultured in media with 2.5 µg/ml CdCl_2_ for 16–18 h.

### Expression of tagged proteins, gene knockout, and rescue

All DNA fragments were amplified using Phusion HSII High Fidelity Polymerase (Thermo Fisher Scientific Baltics, Lithuania) and genomic DNA purified from CU428.2. The primers used are listed in Table S1. To express proteins with C-terminal -3HA, -2V5, or -HA-BirA* tags, native loci were modified by DNA homologous recombination using plasmids with fragments of the coding region and the 3’UTR obtained by modifications of pFAP44-3HA-neo4, pFAP44-2V5-pPur, or pFAP44-HA-BirA*-neo4 plasmids^53^. MluI and BamHI restriction endonucleases were used to insert a fragment of the coding region and PstI and XhoI were used to clone a fragment of 3’UTR (Table S1). For genomic biolistic transformation, the targeting plasmids were digested with MluI and XhoI to separate the transgenes from the plasmid backbones.

To express Adgb with an N-terminal BirA*-HA in the native locus, the 5’UTR and the coding region of *FAP44* were removed from the Neo2-3HA-FAP44 plasmid^53^ and replaced by approximately 1 kb fragments of the 5’UTR and a coding region starting with the ATG codon of the *ADGB* gene, both amplified by PCR with primers listed in Table S1. The 3HA tag was replaced by BirA*-HA sequence. The resulting transgene carries the *MTT1* promoter and the neo2 selectable cassette^54^. The plasmid was digested with SacII and BamHI to separate the transgene from the plasmid backbones prior to biolistic bombardment.

To overexpress proteins as fusions with C-terminal -HA or -GFP tag, the entire coding region was amplified by PCR with the addition of MluI and BamHI sites at 5’ and 3’ ends, respectively (Table S1) and cloned into pMTT1-GFP^55^ or pMTT1-HA^56^ plasmids enabling the integration of the transgene into *BTU1* locus and overexpression controlled by the Cd-inducible *MTT1* promoter^30^. To select transformed *Tetrahymena* cells, based on paromomycin or blasticidin resistance, a neo2^54^ or bsr cassette^57^ was inserted between 5’*BTU1* and *MTT1* promoter. Plasmids were digested with SacII and ApaI restriction endonucleases to separate a transgene from the plasmid backbone.

Approximately 10 µg of the digested plasmid DNA was precipitated onto DNAdel Gold Carrier Particles (Seashell Technology, La Jolla, CA, USA) according to the manufacturer’s instructions and used to biolistically transform CU428.2 cells. Positive clones were selected for 3–4 days at 30 °C on SPP supplied with (depending upon the introduced transgene) 100 µg/ml paromomycin (Sigma-Aldrich, St-Louis, MO, USA) (transgenes with neo2 cassette^54^), 100 µg/ml paromomycin and 1.5 µg/ml CdCl_2_ (transgenes with neo4 cassette^58^), 200 μg/ml puromycin (BioShop Canada Inc., Canada) and 1.5 µg/ml CdCl_2_ (transgenes with pPur cassette^59^), or 60 μg/ml blasticidin (BioShop Canada Inc., Canada) (transgenes with bsr cassette^57^). The positive clones were grown in medium with decreasing concentrations of CdCl_2_ (to 0.05–0.1 μg/ml) and either an increasing concentration of paromomycin (up to 1 mg/ml) or blasticidin (up to 100 μg/ml), or a constant concentration of puromycin to promote phenotypic assortment.

The *Tetrahymena* knockout cells were obtained either by the germline gene disruption approach^25,26^ or by the coDeletion approach^27^. Primers used to amplify fragments of the targeted genes are listed in Table S1. In the case of *CFAP246-coDel* and *ADGB-coDel* we were unable to obtain mutant cells with all copies of the targeted gene disrupted in the macronuclear genome (all analyzed clones had gene knockdown). When we attempted to engineer germ-line *CFAP246* knockout cells, only few paromomycin-resistant clones were found among transformed exconjugants (all 6MP-sensitive), suggesting a deletion of the introduced transgene during the rearrangement of the genome of new macronuclei. Therefore, in the case of the *CFAP246* gene we could investigate only the effect of *CFAP246* knockdown. At least two independent clones of the coDel and germ-line knockout strains were obtained.

To rescue *Tetrahymena* knockout cells, *CFAP69-KO* cells were transformed with a transgene enabling expression of the HA-Cfap69 from the *BTU1* locus, under the control of the *MTT1* promoter (cells were grown without CdCl_2_). Mutants obtained by the coDeletion approach were rescued with the approximately 3 kb fragment of the genomic DNA, encompassing the deleted region and about 1 kb upstream and downstream of the deletion. The rescued cells were selected based on the restored wild-type cell motility.

### Quantitative real-time PCR and RT-PCR

A real-time PCR was carried out using genomic DNA or cDNA as a template and a PowerUp SYBR Green Master Mix (Thermo Fisher Scientific Baltics, Vilnius, Lithuania) with Standard cycling protocol according to the manufacturer instruction in StepOnePlus Real-Time PCR System (AB Applied Biosystems, Foster City, CA, USA). A genomic DNA was purified with Tissue DNA Purification Kit (EURX, Gdansk, Poland) using cell culture protocol provided by the manufacturer. Total RNA was isolated with Universal RNA Purification Kit (EURX, Gdansk, Poland) with the on-column DNAse digestion protocol provided by the manufacturer. Approximately 500 ng of purified RNA was subjected to reverse transcription with SuperScript III First-Strand Synthesis SuperMix for qRT-PCR (Thermo Fisher Scientific Baltics, Vilnius, Lithuania) and oligo dT, according to manufacturer instruction. To compare the levels of PCR products between housekeeping and experimental genes, for each gene a standard curve, with the use of know amounts of DNA (either plasmid or PCR product) was generated. For each sample, the initial amount of the genomic DNA or cDNA was estimated using Step One Plus Software.

### Morphological and physiological tests

To evaluate cilia length^60^ cells were stained with anti-α-tubulin 12G10 antibodies, and confocal images were recorded with a 0.32-µm distance between z-sections. A length of cilia was measured on merges of two to four z-sections using ImageJ bundled with 64-bit Java 1.8.0_172. The cell swimming paths and cilia beating were recorded as described^52,53^.

### Immunofluorescence and transmission electron microscopy

For in situ protein localization, *Tetrahymena* cells were fixed and stained on coverslips as previously described^52,61^. The primary antibodies were used at the following final concentrations: monoclonal mouse anti-HA antibodies (Covance, Berkeley, CA, USA) 1:300, monoclonal rabbit anti-HA antibodies (Cell Signaling Technology, Danvers, MA, USA) 1:300, monoclonal rabbit anti-V5 antibodies (Cell Signaling Technology, Danvers, MA, USA) 1:1600, polyclonal rabbit anti-GFP antibodies (Abcam, Cambridge, UK) 1:6000, anti-α-tubulin 12G10 antibodies (Developmental Studies Hybridoma Bank, Iowa University, Iowa City, IA, USA) 1:300, and the secondary antibodies, anti-mouse or anti-rabbit IgG conjugated either with Alexa-488 or Alexa-555 antibodies (Invitrogen, Eugene, OR, USA), all in concentration of 1:300. After washing, the coverslips were mounted in Fluoromount-G (Southern Biotech, Birmingham, AL, USA) and viewed using a Zeiss LSM780 (Carl Zeiss Jena, Germany) or a Leica TCS SP8 (Leica Microsystems, Wetzlar, Germany) confocal microscope.

To analyze ciliary ultrastructure cells were fixed as described^52^ and samples were viewed using a JEM 1400 transmission electron microscope (JEOL Co, Tokyo, Japan).

### Western blot analyses

For analyses of the total cell proteins, 2–5 × 10^5^ cells from the logarithmic culture were pelleted and briefly washed with 10 mM Tris–HCl buffer pH 7.4. Pelleted cells were suspended in a buffer containing 7 M urea, 2% CHAPS, 40 mM Tris–HCl, pH 7.4, and 10 × concentrated protease inhibitors (cOmplete, EDTA-free protease inhibitor cocktail, Roche Diagnostics GmbH, Mannheim, Germany), and mixed with 5 × concentrated sample buffer (0.3125 M Tris–HCl, pH 6.8, 10% SDS, 50% glycerol, 25% β-mercaptoethanol, 0.02% bromophenol blue). For analyses of the ciliary proteins, ~ 10^7^ cells from the logarithmic culture were pelleted, washed with 10 mM Tris–HCl, pH 7.4, and deciliated^62^.

Total proteins (40 µg) or ciliary proteins, either 40 µg (detection of the tagged protein) or 2 µg (tubulin detection) were run on the SDS-PAGE gel (either 10% or 7.5%, depending on the protein molecular weight), transferred onto nitrocellulose membrane, stained with Ponceau S, and blocked for an hour with 5% non-fat milk in the TBST (20 mM Tris, pH 7.5, 150 mM NaCl, 0.1% Tween 20) at RT. The nitrocellulose membranes were incubated overnight with the primary antibodies diluted in the blocking solution as follows: mouse monoclonal anti-HA (1:2000), rabbit monoclonal anti-V5 (1:1600), and mouse monoclonal anti-α-tubulin (12G10) (1:40,000) at 4 °C. After washing (4 × 10 min in TBST) membranes were incubated with the appropriate HRP-conjugated secondary antibodies (Jackson ImmunoResearch, West Grove, PA, USA) for 1 h at RT, washed and proteins were detected using Westar Supernova kit (Cyanagen, Italy).

For analyses of the biotinylated ciliary protein, samples were run on the 10% SDS-PAGE gel, transferred onto nitrocellulose membrane, and blocked overnight with 3% BSA in the TBST at 4 °C. Next, the nitrocellulose membranes were incubated for 4 h at RT with the streptavidin-HRP (Thermo Fisher Scientific, Rockford, IL, USA) diluted 1:40 000 in the blocking solution, washed and biotinylated proteins were detected using Westar Supernova kit (Cyanagen, Italy).

### Proximity labeling assay and immunoprecipitation

To identify proteins positioned in close proximity to BirA* tagged proteins^23^ engineered *Tetrahymena* cells expressing either Spef2A-HA-BirA*, Cfap69-HA-BirA*, Cfap246-HA-BirA*, Adgb-HA-BirA*, or BirA*-HA-Adgb were grown in SPP medium to a density of 2 × 10^5^ cells/ml, starved overnight in 10 mM Tris–HCl buffer, pH 7.5, and incubated in the same buffer supplied with 50 µM biotin for 4 h at 30 °C (or 16 h in the case of Adgb). Next, cilia were removed and collected^62^, resuspended in the axoneme stabilization buffer (20 mM potassium acetate, 5 mM MgSO4, 0.5 mM EDTA, 20 mM HEPES, pH 7.5 1 × protease inhibitors (cOmplete, EDTA-free protease inhibitor cocktail, Roche Diagnostics GmbH, Mannheim, Germany) and treated for 5 min on ice with 0.2% NP-40 (final concentration) to remove the ciliary membrane. Collected axonemes (10 min at 21,100 × *g* at 4 °C) were lysed in a buffer containing 50 mM Tris–HCl, pH 7.4, 0.4% SDS, 0.5 M NaCl, 1 mM DTT, 1 × protease inhibitors for an hour at RT. After centrifugation (8000 × *g* at 4 °C) the supernatant was diluted with 3 volumes of 50 mM Tris–HCl, pH 7.4, and incubated overnight with 100 µl of streptavidin-coupled Dynabeads (Dynabeads M-280 Streptavidin, Thermo Fisher Scientific, Waltham, MA, USA) at 4 °C. After washing (6 × 5 min with washing buffer: 15 mM Tris–HCl, pH 7.4, 150 mM NaCl, 0.1% SDS, 0.3 mM DTT) at 4 °C, resin-bound proteins were analyzed by western blot and by mass spectrometry. For mass spectrometry analysis, resin-bound proteins were reduced by 60 min incubation with 5 mM tris(2-carboxyethyl)phosphine (TCEP) and cysteines were blocked with 8 mM methyl methanethiosulfonate (MMTS). After overnight digestion with 0.5 µg of trypsin (Promega, Madison, Wisconsin, USA) tryptic peptides were acidified with 0.1% trifluoroacetic acid (TFA) and subjected to LC–MS/MS analysis.

To identify proteins that co-immunoprecipitate with HA-tagged Cfap69 or Adgb, engineered *Tetrahymena* cells expressing Cfap69-3HA or Adgb-3HA under the control of the respective native promoters, were grown in SPP medium to a density of 2 × 10^5^ cells/ml and washed with 10 mM Tris–HCl buffer, pH 7.5. Next, cilia were removed and collected^62^, and resuspended in 10 mM Tris–HCl buffer, pH 7.5, supplemented with 2 × protease inhibitors, and combined with an equal volume of 2% NP-40 and 1.2 M NaCl in 80 mM Tris–HCl buffer, pH 7.5. After 20 min of incubation on ice, the axonemes were pelleted at 21,000 × *g* for 20 min. The collected supernatant was diluted 4 × with 40 mM Tris–HCl, pH 7.5 (supplemented with protease inhibitors) to reduce NP-40 and NaCl concentration and incubated with anti-HA-conjugated resin (Pierce HA Epitope Tag Antibody Agarose conjugated, Thermo Scientific, Rockford, IL, USA) with rotation, overnight at 4 °C. Beads were washed six times (5 min each wash) with 40 mM Tris–HCl, pH 7.4, 150 mM NaCl, 0.25% NP-40, and 0.5 mM EDTA at 4 °C. Resin-bound proteins were separated on 10% SDS–PAGE gel and silver-stained^63^ or eluted from resin with 50 mM NaOH and analyzed by mass spectrometry as described above.

### Total cilia proteome analyses

Approximately 5 × 10^7^ wild-type (control) or mutant cells from logarithmic cell culture were deciliated^62^. Cilia were lysed with 7 M urea, 2% CHAPS, 40 mM Tris–HCl, pH 7.4 and 100 µg of protein per sample were prepared according to FASP protocol with minor modifications^64^. In brief, the protein sample was diluted up to 200 µl with urea buffer (8 M urea in 50 mM ammonium bicarbonate) and incubated for an hour with 50 mM TCEP at 60 °C to reduce cysteine residues. After ultrafiltration onto 10 kDa molecular weight cut-off ultrafiltration units (Vivacon, Sartorius Stedim, Goettingen, Germany), samples were washed twice with urea buffer and incubated with 50 mM IAA for 30 min in dark at RT. Modified proteins were washed three times with 8 M urea buffer followed by three rinses with 50 mM ammonium bicarbonate to remove denaturant. Each time the filters were centrifuged at 14,000x*g* at for 15 to 30 min until the membrane was dry. Digestion was carried out overnight using trypsin/LysC mix (Promega, Madison, WI, USA) in a 1:25 enzyme-to-protein ratio at 37 °C on vortex. Peptides were collected from filters by centrifugation and two additional washes with 0.5 M NaCl and 50 mM ammonium bicarbonate, respectively. Combined eluates were acidified with trifluoroacetic acid (TFA) and vacuum-dried. Peptides were reconstituted in 0.1% TFA with 2% ACN and subjected to LC–MS/MS analysis.

### Mass spectrometry

Three µg of peptides from each total cilia proteome sample and one third of proximity labeling assay and immunoprecipitation samples were analysed on an LC–MS system composed of an UPLC chromatograph (nanoAcquity, Waters, Milford, MA, USA) directly coupled to a Q Exactive or Elite mass spectrometer (Thermo Scientific, Rockford, IL, USA). Data acquisition for analysis of total cilia proteome was solely performed on Q Exactive system. Peptides were trapped on C18 pre-column (180 µm × 20 mm ,Waters, Milford, MA, USA) using water containing 0.1% FA as a mobile phase and then transferred to a nanoAcquity BEH C18 column (75 µm × 250 mm, 1.7 µm, Waters, Milford, MA, USA) using acetonitrile gradient (0–35% ACN in 160 min) in the presence of 0.1% formic acid at a flow rate of 250 nl/min. Data acquisition was carried out using a data-dependent method with top 12 precursors selected for MS2 analysis after collisional induced fragmentation (CID) with an NCE of 27. Full MS scans covering the mass range of 300–2000 were acquired at a resolution of 70,000 with a maximum injection time of 60 ms and an automatic gain control (AGC) target value of 1e6. MS2 scans were acquired with a maximum injection time of 60 ms and an AGC target value of 5e5 with an isolation window of 3.0 m/z. Dynamic exclusion was set to 30 s.

### Qualitative MS/MS data processing

MS/MS data were pre-processed with Mascot Distiller software (v. 2.6, MatrixScience, London, UK) and a search was performed with the Mascot Search Engine (MatrixScience, London, UK, Mascot Server 2.5) against the NCBInr RefSeq *Tetrahymena thermophila* SB210 database (31/12/2018, 54,102 sequences; 33,890,079 residues) for total cilia proteome samples or older version 20111116 (27,769 sequences; 16,635,335 residues) for BioID/IP/gel samples. Search parameters were set as: enzyme—trypsin, missed cleavages—2 or 1 for BioID/IP/gel samples, fixed modifications—carbamidomethyl (C) or methylthio (C) for BioID/IP samples, variable modifications—0xidation (M), instrument—HCD. Moreover, in the total cilia proteome samples, to reduce mass errors, after a measured mass recalibration, mass tolerance settings of the peptide and fragment were established separately for individual LC–MS/MS runs^65^, resulting in 5 ppm (for parent) and 0.01 Da (for fragment ions) values. Data were searched with automatic decoy option.

The statistical significance of peptide identifications was estimated using a joined target/decoy database search approach. This procedure provided q-value estimates for each peptide spectrum match (PSM) in the dataset. All PSMs with *q*-values > 0.01 were removed from further analysis. A protein was regarded as confidently identified if at least two of its peptides were found. Proteins identified by a subset of peptides from another protein were excluded from analysis. The mass calibration and data filtering were carried out with MScan software, developed in-house (http://proteom.ibb.waw.pl/mscan/).

### Quantitative MS data processing

The lists of peptides that matched the acceptance criteria from the LC–MS/MS runs were merged into one common list and overlaid onto 2-D heat maps generated from the LC–MS raw files A more detailed description of the quantitative extraction procedure implemented by our in-house software is available in^66^. The abundance of each peptide was determined as the height of a 2-D fit to the monoisotopic peak of the tagged isotopic envelope. Quantitative values were next exported into text files, along with peptide/protein identifications, for Diffprot softwere for non-parametric statistical analysis of differential proteomics data^65^ Diffprot was run with the following parameters: number of random peptide sets = 10^6^; clustering of peptide sets—only when 90% identical; normalization by LOWESS. Only proteins with q-value below 0.05 or those present in only one of two compared analytical groups were taken into consideration during further analysis.

### Phylogenetic analysis

The orthologs of Spef2, Cfap69, Cfap246, Cfap174, and Adgb were identified in the NCBI database using Blastp search and *Chlamydomonas* or human proteins as baits. The sequences of *Tetrahymena* orthologs were obtained from Tetrahymena Genome Database (TGD, http://ciliate.org). The protein amino acid sequences were aligned using the ClustalX2 program^67^, edited using the SeaView program^68^, and the similar/identical amino acid residues in the multiple sequence alignments were highlighted using the GeneDoc program^69^. The domain analyses were performed using InterPro (https://www.ebi.ac.uk/interpro/)^70,71^.

## Supplementary Information

Supplementary Information 1.

Supplementary Information 2.

Supplementary Information 3.

Supplementary Information 4.

Supplementary Information 5.

Supplementary Information 6.

Supplementary Information 7.

Supplementary Information 8.

Supplementary Information 9.

Supplementary Information 10.

Supplementary Information 11.

Supplementary Information 12.

## Data Availability

Data generated or analyzed during this study are included in this published article (and its Supplementary Information files).
