# Supplementary material for: Composition and function of the C1b/C1f region in the ciliary central apparatus
Source: Sci Rep. 2021 Jun 3;11:11760. doi: 10.1038/s41598-021-90996-9 (PMC8175508; doi:10.1038/s41598-021-90996-9)
Supplement: Supplementary file 1 — Supplementary Information 1. [file 41598_2021_90996_MOESM1_ESM.pdf]

Supplementary Information

Composition and function of the C1b/C1f region in the ciliary central apparatus

Ewa Joachimiak*[1], Anna Osinka[1], Hanan Farahat[1], Bianka Świderska[2], Ewa Sitkiewicz[2], Martyna Poprzeczko[1, 3], Hanna Fabczak[1], Dorota Wloga*[1]

[1]Laboratory of Cytoskeleton and Cilia Biology, Nencki Institute of Experimental Biology Polish Academy of Sciences, 3 Pasteur Street, 02-093 Warsaw, Poland. Phone: +48 22 5892338, fax: +48 22 822 53 42

[2]Mass Spectrometry Laboratory, Institute of Biochemistry and Biophysics Polish Academy of Sciences, 5a Pawinski Street, 02-106 Warsaw, Poland. Phone: +48 22 5923473, fax: +48 22 6584766

[3]current address: Department of Immunology, Medical University of Warsaw, 5 Nielubowicz Street, 02-097, Warsaw, Poland.

e.joachimiak@nencki.edu.pl

d.wloga@nencki.edu.pl

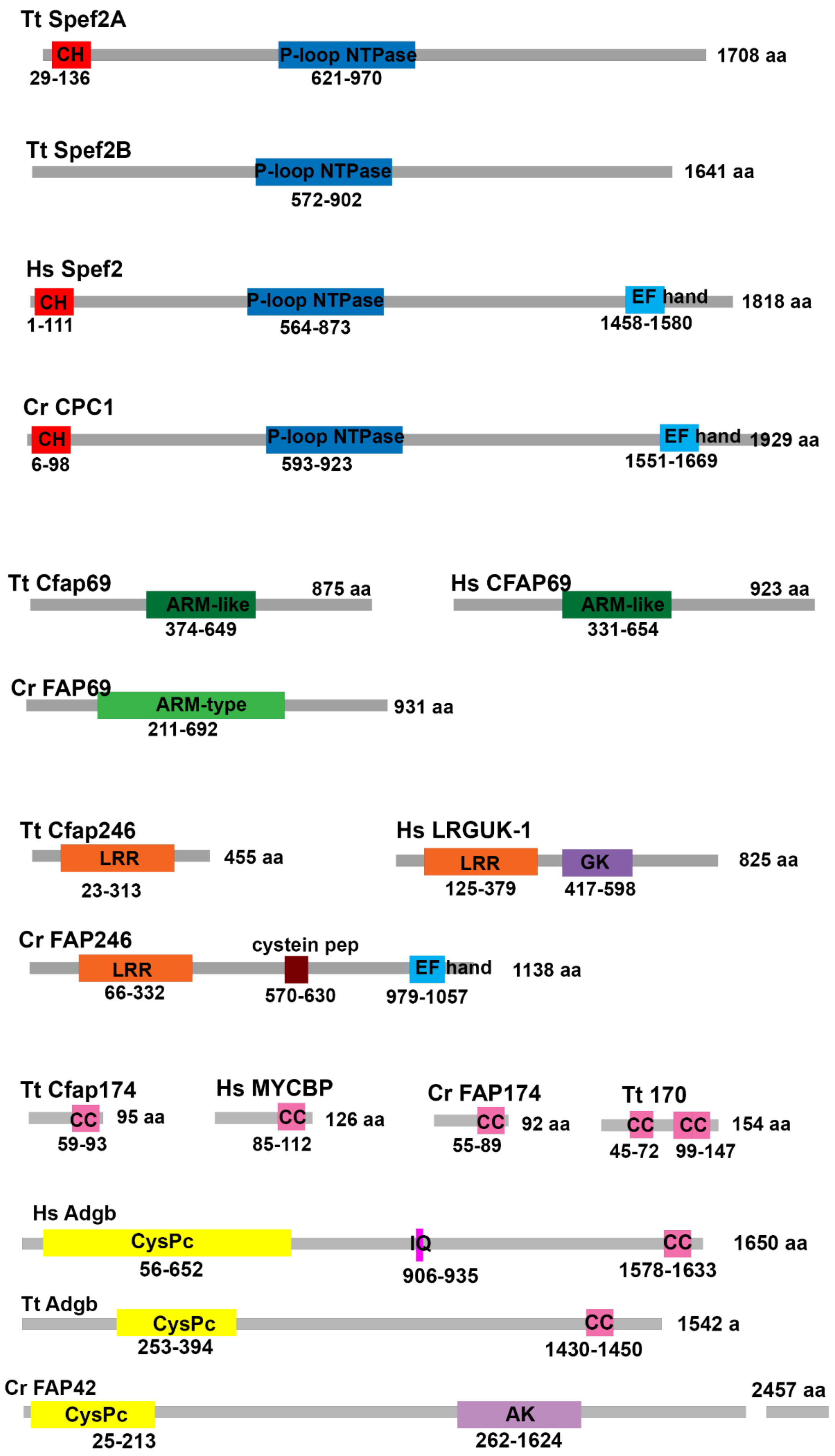
Tt Spef2A
CH
29-136
P-loop NTPase
621-970
1708 aa
Tt Spef2B
P-loop NTPase
572-902
1641 aa
Hs Spef2
CH
1-111
P-loop NTPase
564-873
EF hand
1458-1580
1818 aa
Cr CPC1
CH
6-98
P-loop NTPase
593-923
EF hand
1551-1669
1929 aa
Tt Cfap69
ARM-like
374-649
875 aa
Hs CFAP69
ARM-like
331-654
923 aa
Cr FAP69
ARM-type
211-692
931 aa
Tt Cfap246
LRR
23-313
455 aa
Hs LRGUK-1
LRR
125-379
GK
417-598
825 aa
Cr FAP246
LRR
66-332
cystein pep
570-630
EF hand
979-1057
1138 aa
Tt Cfap174
CC
59-93
95 aa
Hs MYCBP
CC
85-112
126 aa
Cr FAP174
CC
55-89
92 aa
Tt 170
CC
45-72
CC
99-147
154 aa
Hs Adgb
CysPc
56-652
IQ
906-935
CC
1578-1633
1650 aa
Tt Adgb
CysPc
253-394
CC
1430-1450
1542 a
Cr FAP42
CysPc
25-213
AK
262-1624
2457 aa

Supplementary Figure S1. Domain analyses of *Tetrahymena* (Tt), human (Hs) and *Chlamydomonas* (Cr) orthologs of C1b/C1f-related proteins. The number of amino acids building a protein is indicated near the protein C-termini. The position of the amino acids forming marked domain is indicated below the domain. Domains were predicted using InterPro (https://www.ebi.ac.uk/interpro/). AK, adenylate kinase; ARM-like (type), armadillo repeat (ARM)-like (type) fold; CC, coiled-coil; CH, calponin homology; Cysteine pep, papain-like cysteine peptidase; CysPc, calpain-like protease; EF, EF-hand motif; GK, guanylate kinase; IQ, Ile-Gln residues containing calmodulin binding motif; LRR, leucine-rich repeat; P-loop NTPase, P-loop containing nucleoside triphosphate hydrolase.

(a) Spef2

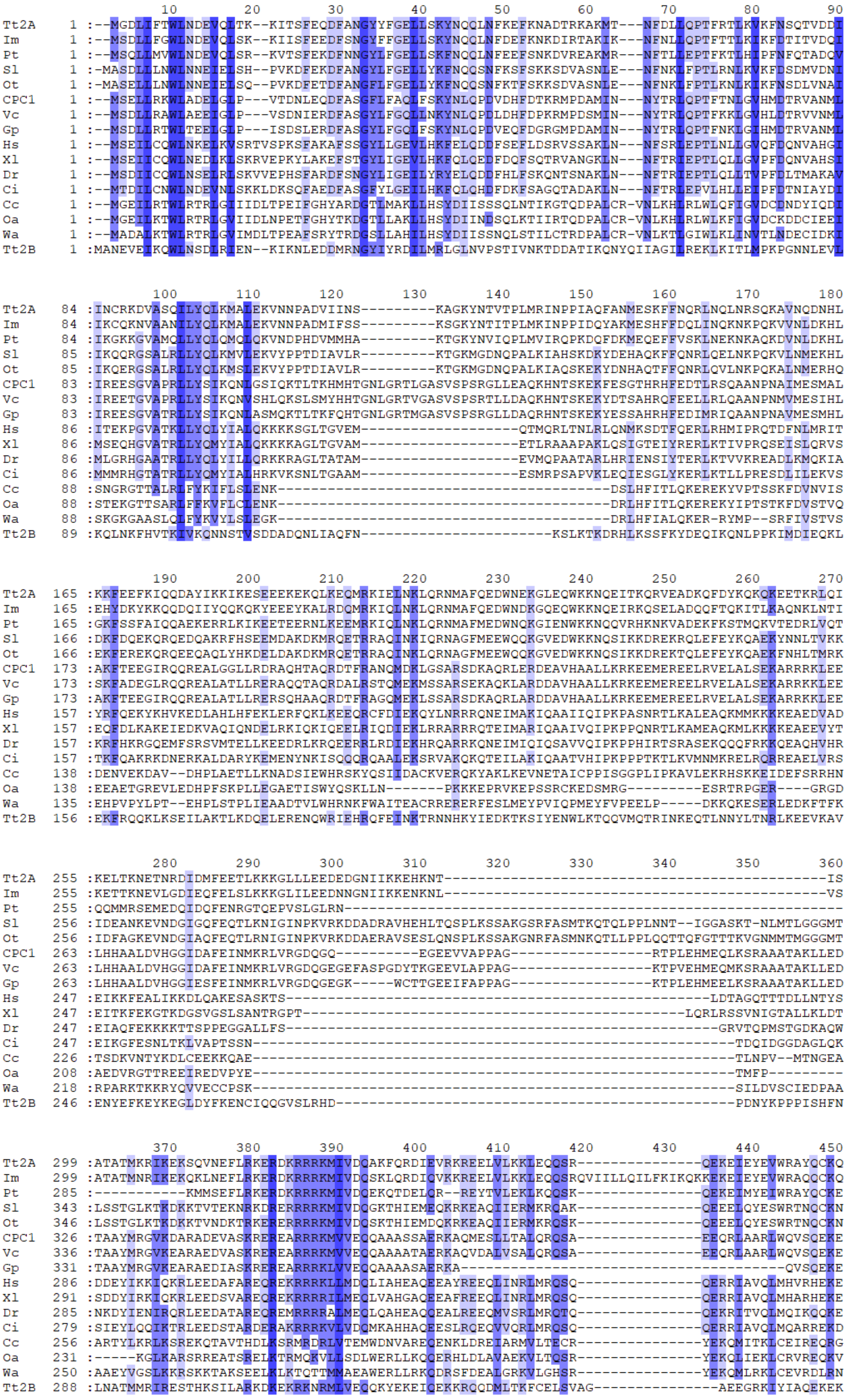

```
                  460       470       480       490       500       510       520       530       540
Tt2A  374 :IIIQDRFLRDEKYKQKNEVNVVNAKYKEEELLKILRQENENDILSKLNRQREIEISEKQLNRKDVYSKCRNLIEQLVDLANICYEQQQQQ
Im    389 :IIIENRRIRQDKYKQKNEVMVINAKYKEEEMLKQLKEENNKDIKSKLVRQNEIEIQQKQMQRKQNYFKCQDIVHQFIDLANICYDQQQLN
Pt    347 :VICENRQLRDENYRNKQELNVINQKFKEEEMLRALQSEFNEEIELQQKRQLEINVEYKLQIRQSNYEKCKKLVEILFEIEEQLYEHLQNN
Sl    418 :VIIEDRKLREARYEKRRELDQQTAIWREEEMMKSLRDQMQREMEIFQERDQEMRIVHKQSKREKRNEFGYQLFDAIFDIANEAYIHQQKQ
Ot    421 :VIIENRKLREARYDKRRELDQQTAIWREEEMMRSLREQMHRELETFKERDSEMRIVHKQSKREKRNGFGQVLFDAIFDIANETYVHQQKQ
CPC1  401 :VMRENRLLRERQYAERRERDWEETLRREVELHRSMRETYEAEAALEMEAWRAAQQARAEAKSAKHAAFARDVAYQLVSLAERSAEYRAAT
Vc    411 :VMRENRLLRERQYAERRERDWEETLRREAEMHRSMREAYEAEAALEMEAWRAAQQARAEAKAAKHAAFAREVVYQLVGLAERAAEYRLAT
Gp    382 :VMRENRLLRERQYAERRERDWEETLRREVELHRSMREEYEAEAALEMEAWRAAQRARAEAKAAKHAAFARDMAYQLVALAERAAEYRAAT
Hs    361 :VLWQNRIFREKQHEERRLKDFQDALDREAALAKQAKIDFEEQFLKEKRFHDQIAVERAQARYEKHYSVCAEILDQIVDLSTKVADYRMLT
Xl    366 :VMQQNRMFREKQYQERREREFQESLDREAALQKQAKLDREEEIRKVQQLHDQIAAERAEARYKKHYNLCQEIMNQILDFVTKTGEYRELT
Dr    360 :VLRENRIFQERQYQERRQRDFQEALDREAVLAQQERLERSEQLRIEHELHKQLAEERAQARYQKHFGICRDILGQIVDLATKAGEYRLLT
Ci    354 :VIRKNRIFQEKQVEARRLQDFQDALDKEAALNKLAKEEYEEEIQRELELHQRIRSQHAHEKYRQHYNFCQEVLNAITDFACKIAEYRELT
Cc    331 :RIARNRQIVEDLVQKTKESRDRLEQNSVREVIVREAEEFDAECNRMCELHQRLRDEKIRAIREKHFRLCNKAVDDIVDMSVKMAEYRLCT
Oa    301 :RISRNRLIVEELITRTKEGEAVIEQDRSRETSLRDAEDVDAECRRLCELRKMLKTEKMRKVRERHEGLCAEVLKDLVDLSLEVSRYRSQN
Wa    325 :RIVENRRIVDAILLKVRESERRLKEDRREEAIKEEREKVEMEVCRMRELRRRIREEKVRKMKEKHQSICSEIINDLADISVKIAVYRQEN
Tt2B  363 :IVVRNRIEKENQIQKERMDYFDQKHEEFREKAIAEEIERKKQWKLQKVEYMELNLQNKIQKRKKHSKTCQDLIDIILDVTELCHNAQRQN

                  550       560       570       580       590       600       610       620       630
Tt2A  464 :DNENIDSHFLNDQIKLFIDNKPLLKEKQFRNYETIPNLRN-------------------------------------------------Q
Im    479 :DTNLIDTNLFQNQLHLFINEMNLIKTKQFANADIIQNLKQ-------------------------------------------------Q
Pt    437 :DSNEFNKQFTRENHHLFKQGMELIPYKRFRTYDPIKEMKK-------------------------------------------------Q
Sl    508 :DSEDIDSRCWHEWLQLFVADLPISKD-----DTMIERDFM-------------------------------------------------Q
Ot    511 :DSTEIDPRNWHEWLQLFINELPVGKVS---HEEVVTVDEQ-------------------------------------------------E
CPC1  491 :G-NLVPRREWREWLTMFVAGDPQLGPP---APPVGAEAEA-------------------------------------------------A
Vc    501 :G-NLVPRREWRQWLAMFAAGDPQLGMP---PPPPGSEAQL-------------------------------------------------A
Gp    472 :G-NMVPRREWRQWLALFTAGDPALGPP---APPQGEVAEA-------------------------------------------------E
Hs    451 :N-NLIPYKLMHDWKELFFNAKPIYEQASVKTLPANP---------------------------------------------------SRE
Xl    456 :N-RLIPIKLMREWKEVFVAGYPLYDKAPIDDLPPEP---------------------------------------------------TPE
Dr    450 :A-NLIPVKLMQEWMELFYSGKPLYEVACVDPKPTEP---------------------------------------------------TPE
Ci    444 :D-KKVPEKLVREWRDFVIMGKPLYISSDIALR----------------------------------------------------------
Cc    421 :K-RSVPTTLWNEWQTLFVEGQPILDLLDRPE-LIVNTDSG--------------------------------------------QGANEV
Oa    391 :E-NEIPESLWREWRSLFLKSQPMVQHLENLEDLVEQRVSN--------------------------------------------SSLEEI
Wa    415 :G-NHIPNCIWNEWKMLFLKNQPIFEPMDHFDDVEEIEDVEKIKKDMKVSENDEVMVKKKYEARKNSGEEIKNEEGIKIGKELKAEEKQRL
Tt2B  453 :NSIRVPEQYWRDLMERFAEGRPLCEEKVVTIFEQQQQTQR--------------------------------------------------

                  640       650       660       670       680       690       700       710       720
Tt2A  505 :EQSQTESKDFLADQELQDYLE---GRGEWIPQHRFEYQGEDPLNND----------------------------------EKYIPNQTLN
Im    520 :YEDQQESKKFVISKQFQDYLT---GQGIWVPQHNFPQNEED---------------------------------------EKNIPNHAIN
Pt    478 :ESLNYKQKMFLAKMEMQDYLE---GTGAWD------------------------------------------------------IYQAQN
Sl    544 :DSMKEVISANLDQVELEDYLK---NQGQWPEALIAENQPNLEQFLTGQTES-APAAGAKGGKAPAPSKAAAADQIVLEEGDTELPLQAPN
Ot    549 :IPLTDQIETQLDQIELEDYLK---NKGQWPTDLIEQNRPNLEHFLTGQVDQGTAAAGAKGGKA-APAKAAASEQIQLEEGDTFLSSTLDN
CPC1  528 :EAEVREACLRVGRALVDDYLA---CAGDWLRV----------------------------------------------------DGPIGH
Vc    538 :EADAREATSRLGRALVGDYLA---CTGDWVRE----------------------------------------------------QGPIGH
Gp    509 :ANEAREASLRLGRALIGDYLA---CAGDWARE----------------------------------------------------EGPIGH
Hs    489 :QLTELEKRDLLDTNDYEEYKN---MVGEWALP----EEMVD-------------------------------------------NLPPSN
Xl    494 :QLQDMEKQSLLDENDYEEYKS---MTGEWSPH----EASSV-------------------------------------------KAPAPN
Dr    488 :QIIELEKLQILNNQDYDEYVA---MIGEWALD----EETER-------------------------------------------QTNAGN
Ci    475 :EKIELEKENLLDDQDFSEYKN---MVGEWKPL----EDTEI-------------------------------------------SKPANN
Cc    465 :FQAEIVRQEVLNTADILNYVKG--SAPPWDQVVSVDDVYNE-------------------------------------------KSLKLG
Oa    436 :QRIELGRQNVLDEADFENYHR---IKPPWEAFSPGPESEER-------------------------------------------QTLLPG
Wa    504 :FQLGLERQQSLADADFESYRD---LSSPWDQFVPKREAGVE-------------------------------------------EVYRLG
Tt2B  493 :QAHEAEISEELDQQLSMMFTDYNLGFQMWQPFHQFEQPENQ------------------------------------------ILNHAYN

                  730       740       750       760       770       780       790       800       810
Tt2A  558 :NYHLGNLVVGIMKDFYSSEIEQSQREVINHFDWLPLKLAILGYQFSGKKTIANIISKKYG-VKLLSIDILMNELIEKYHAYQRQVQQQQR
Im    568 :NYFLGDLIIQILEKYYEQKLEEQKKIKNNHFDWLPIKIAILGYQFSGKKTIANLLQQKYN-VKILSVEKLLNDLIDTWRQYQQKQQQIQQ
Pt    511 :NYNLGNLVRYLIESQFKNSIQEMEQ---TDIPYVPFRGSLIGFSFSGKKTISELLAKKYG-IQLLSIDVIINELLEYIKKYEQGEEHSQV
Sl    630 :NYLLGDALELIINMNFDQRENHKKP---KMPSYLNLKLCFVGYAFAGKKTQANKLKEVFGNLDIYYLNDLVSQAVSFFEQNPESIQRALQ
Ot    635 :NFDLGEAIENIINMNFDARENHKRP---KVPAYLNLKLCFIGYAFSGKKSQAQKLKEKFQDLNLYHLAELVAEAVSFYESHPNPLDIITY
CPC1  563 :DELLGQIVGELAVLARQPPPQPELP----AIETLPLRLAVLGAPFAGKTCVAQELSRRYK-LRVLEPEALVAEAMAAAQEFEAATG----
Vc    573 :DELLGQVVGELAIQSREPAPQPELP----AIENLPLRLAVVGAPFSGKTTVAQDLARKYR-LLLLDPEALVADAMAAAQAHEAAVAAAAT
Gp    544 :DEVIGELVGELAILSREPPPQPELP----AIESLPLRLAVVGAPFAGKTSVAQELARRFK-LQLLDPEVLVAEALKAAQEFEAASG-ISA
Hs    529 :NCILGHILHRLAEKSLPPRAESTTP----ELPSFAVKGCLLGKTLSGKTTILRSLQKDFP-IQILSIDTLVQEAIQAFHDNE---KVSEV
Xl    534 :NNILGYVVHRLLEIISPPMAPTPPP----VFPPFPIKGCVLGKICSGKSTCLQHLAQAYS-IQILSVDALLDEAIQAFHVEELEKTENVV
Dr    528 :SDILDHVLNRLRNMIVPPTVSTPPP----LFPRFTIRACMLGKTFSGKTTCLSRISSTLN-IHVLSANTLIQDALSAYQQEKQAASESQN
Ci    515 :N-ILGHILHRMFSIVAPTPPPSPPP----VFPEFPIKVAVLGKMFAGKSSVISSLVKQHR-IVQLSMNEILSSAVDALSERN--------
Cc    510 :ETVLGYIVHKLLRYLYPKPSDPQPL----SMPNISVAAIVLGVLDSTLYPALQELLKNHK-IRLVRVEDAINYCLRCYKESLS------I
Oa    480 :EQVLGYVVHTLLRVLYPYPKDPESP----PLPRFSTSAVVLG-LSPELRSPLQGLLEKSG-ILLVRLEDAINFCLSRYKEEMRDSRFIDL
Wa    548 :RTVLGYIVHRLLEILYPYPAEVMGC----PVPRVKVAAIVLGVTNATSYEQLRGLLKNTE-IRLLTMEDAINHCLESYKREMRDVEYIDL
Tt2B  541 :DDQLGDFLHQVIDKIYPQKAIQSKP--HDIPDHMPLRISIIGNRFSGKKSLSQLIKQKYG-VEIIDVYDVIDQAIKYAFPPPEDENEKKK

                  820       830       840       850       860       870       880       890       900
Tt2A  647 :EEKIRSSKSSSRPNNNSVGQIERNDVSGGSNRNNKSQANIAAARISQQQIEEENQQVELDEESIQLAQKIEQCLYTGQNIPDEYYIKLIQ
Im    657 :K--------------------------------------------------------------ILKTQEIENILLDGKEIPDEYYIKLIM
Pt    597 :WD----------------------------------------------------------QQQIELGQQISGYLCQGEEIPEELYIKAIV
Sl    717 :QN--SEEEAIQDDLEISEDSEIDEELN-------------------------------AEEDFRQCGQDISELLKEGIEITDDIYVRLFI
Ot    722 :QDNANQYASPDQDLDLSVDSEIQEEYN-------------------------------ADEDFRLCGELICNLLKDGTEISDEIYVQLFV
CPC1  644 :-VPADQVPEAAPQED-----GDAAAAAELQAGSASRPGTAATAAAG-------------PSAKARLGAQIAAALKAGGDVPDEALVGLVV
Vc    658 :AAAAPPVPPASSQGSRPLTPASPAAAAVAAAAAAAAEAAAAAAAAG-------------PSVKAQLGAQVAAALQAGGDVPDEALVRLVI
Gp    628 :AAPPESWPRLVTLPAGESHPAPGAAVSDTAGATGEGAVEGEEADSGPK----------PPSVKARLGAQIAAALAAGGDLPDEVLVALVV
Hs    611 :LPIQKNDEEDALPVLQ-------------------------------------------EEIKESQDPQHVF-SAGPVSDEVLPETEGAN
Xl    619 :EPIQEGADKENKPMDN-------------------------------------------SAPVTKGEEIPVA-ESDLKPAPAEPGESDKD
Dr    613 :ESLEKNVSQTEHTTEQ-------------------------------------------LQKDEQE-------TSSPLESGPQPHEASSG
Ci    591 :---EENEQSED-------------------------------------------------------------------------------
Cc    589 :NSVSQQN--LDNIGSH-------------------------------------------VTARSSRTDVEVKTAENYVTSNMAD------
Oa    564 :EEVAEMNKGPGRVSEV-------------------------------------------VGSKRSGLERGSKRLKESARSRMVLPEEGGP
Wa    633 :NIISATARDIKRLDAK-------------------------------------------NKMDDLG-ERHLKKIERSAKLVTSLQR-AAE
Tt2B  628 :NAAKKQTKKGQVEEEKPD-----------------------------------------NPQLRELGQQANEMKTQNIPYTDEILVRAAL
```

```
                        910       920       930       940       950       960       970       980       990
Tt2A   737 :NVLKQIHPEVTEQQFLEQLAQKSIPSE--NEQLLKLP-----------------------------NLGQDLQMPPTKEDTFRNALINRT
Im     685 :KELNKIHPEVSEEQFLENYEKILYLNDNHNDQQLCID-----------------------------NENRDIEFPLTQEDIFKNQLSNKN
Pt     629 :QKIKRLFDKQEFEQIYEEYKAKYSSFN--PRQSRTID-----------------------------EFNDD---GINWEDFHRNNMIN-N
Sl     774 :AKLRLTYPHKSKKQLRRELKSKVEKEREITQKIQTVENEIQELNGGG---------NGENPGGSRRRRKKDPVQLQDELDKLNKELQTAQ
Ot     781 :AKLRKTYPYKSKDTLRRELKQKVLKEREITSKIQAVENELAELSN-----------QDESANNARRRRRRDPKQLQEELEKLNKELADAQ
CPC1   715 :LGMKESKDWAPPVEVDPKAKGKAAPKPAAPP-------------------------------------------AGSKSAGAPAAPHPDT
Vc     735 :LGMKEAAEWVPPVQADPKAKTKGSATKASAATS---------------------------------------GKAAAAAAGATAAQQQPP
Gp     708 :QGMKDSKDWSPPSAVDAKAKAKAAPAAPKPG--------------------------------------------TAPAGGKAAAPPPPP
Hs     657 :ADKTPKAEEVKSSDSFLKLTTRAQLGAKSEQLLKKGK------------------------------------SIPDVLLVDIIVNAINE
Xl     665 :LSQHQILKSAK-EDTVPKLSARAQLGGLVETHLKSGK------------------------------------SVPDELLTNILVEAINR
Dr     653 :LHEKEDKEESK---AIPKWSSRAQHGAAVEKVLRTGQ------------------------------------AVPDQLLVDIIVDAIRN
Ci     599 :------------------LANQNALAEKAVEHLKKGE------------------------------------IVPNEVLVDLLVDRIHK
Cc     628 :-KRTQTPRIIPYDDFEPDVRKAAYYGKWANDYLTLGE------------------------------------PITNELSTKMLVEYLKS
Oa     611 :SKQTQTPRQIPFDDLDPTLTDAAYMGRWAHEFLTLGQ------------------------------------PVSSELGARVLMEYLKS
Wa     678 :EKQTQTPKHIPYDDMDPILSDAACIGKWTYEFLTLGQ------------------------------------PISNELNTKILIEYLKG
Tt2B   677 :IQIKQIFPVVTPDQLIQQLADAKIKEQERQAQMKKEWEDYLKKKEKSNPKKAAGKEGKKNVGSKVEIEVIEDPPQETYEEAVYRLMNQKE

                       1000      1010      1020      1030      1040      1050      1060      1070      1080
Tt2A   796 :HKYTSGIVLLDFPHTYEQAKLLEKEISGFLPKDEIIQSEFEDSIQKAQKIVKPTKVETKPRQLIKPGLDLVIYIDIDKKE-SLRRALG--
Im     746 :HKYTNGVILLDFPHSYEQAKLLEKELSGFMPKDEVIQSEFEKSVQKASLIVKGKEVEIVKRSLIKAGLDNVFYINIDKTE-SLRRALG--
Pt     684 :KPYCKGWLLVGFPHNYEQAKLLEKHLTGIEPQDELTP--LQARLQEAARVVKPVDYSVIPRKKQESGVGVCVYLEMPSNELCLRRAIG--
Sl     855 :AQDSKGWILVDFPATFAQAKLLEQALSGYVPPQEQDKIDREAQIEEAFLLVQPNAKEVPPKKLLKSGLDAVIWIDCSRDE-CMRRALG--
Ot     860 :APENKGWILVDFPSTFAQAKLLEAALSGYVPPQEKNQIDREAQIADAQLLVKGYSKPEPPKKLLRSGLDGVIWVDCSRDE-CMRRALG--
CPC1   762 :G---RGFVVDGFPRTAAQAVLLERMLTGLDLDSEQALIDAASVIAPPPASALP-----QVARPLVSGLDAVVVCGLADPELALKRALG--
Vc     786 :GNEQRGFVVDGFPRTAAQAVLLERMLTGLDLESEQALIDSASTVAPPPSSALP-----QVGRPLVSGLDAVIVCDLADPELALKRALG--
Gp     754 :PPEGRGFVVDGFPRTAAQATLLERMLTGLDLDSEQALIDAASTIAPPPQSALP-----QVGRPLMSGLDAVVVCGLADPELALKRALG--
Hs     711 :IPVNQDCILDGFPMTLNQAQLLEEALTGCNRNLTEVERKKAQKSTLAIDPATSKE-----IPLPSPAFDFVILLDVSDTSSMSRMN----
Xl     718 :IPANTGWIMEGFPMTETQAKLFEKALSGSHTDLHAIKGNKDKFSSLVTDPAAPKD-----VPVSPSALDFAVIIEISDSEVLQRVS----
Dr     704 :IPADSGWILDGFPVDISQAQMLEKALN-----MTELDGTKTQN-NLSTNKNGPKD-----AAPPSPALDLVVLLDVSDEQVLERATTQPP
Ci     635 :IPAGTGWILDGFPTNIEQALLLEKALTGSDVSYSKDKKDKRKS-SVVSDPKPVKA-----KPATPPGLHMVVHLDVSDATILSRACG---
Cc     681 :LTEAKGWALINFPMTYDQMALLEFLLTGRKVPQIIEEANDVNGNIEEVDPVPSR-----------------ITFEDESEDEFALKR----
Oa     665 :LEGVEGFALLDYPMTYEQMSCLEFSLSGLRVP-PEEDVHSMDENIEDFEPPSSR-----------------ISFEVEDPDRFENQR----
Wa     732 :IGDVEGWILINYPNTYEQMAMLEKALTGREIP--AEPVNLTDIEVEDIDPLSPR-----------------IVFESDEVDIFAISR----
Tt2B   767 :FEYTPGFVLVNFPQNVKQAKLLEEGLTGFVMEEERLNPEGEQIKEQIAQLVRAGEKT-PDTSLKQGGIDLVINLNVDETIAEKRAANR--

                       1090      1100      1110      1120      1130      1140      1150      1160      1170
Tt2A   883 :-------RRYDPLTKQNFHLDDNPPPVDNAPLIERLEQIREYGTSELAIVDKNCELDNNFKQLKEWYSMFGNKFEIR-------------
Im     833 :-------RRYDPFTKQYYHLDDNIPPVDNAPLVERIEGIMQQ--SEIGIVDKNCLLDNNYQELKEWYSMFG-------------------
Pt     770 :-------RRYDNHNHSLYHLESNTPPVDNAPLVERIKPLYEVDNLQQLISDKNAYFMTQMQGIQNWY-----------------------
Sl     942 :-------RRFDNVNEKVYHIEDQTPLTTNAPLCERLQPMDEEDNSEATLIDRWISYDQNAQGLENWLKQFG-------------------
Ot     947 :-------RRFDNVNEKVYHIEDQAPLTTNAPLCERLLPMEEEDNSESTLIDRWISFDQSSKSLENWLSQFG-------------------
CPC1   842 :-------RRLDPQTGRVYHLEFDPPPSNDPGLSARLQEVADASNDAQQIQHRLMSQAELAGPLDDWLRRFSRLRRPVDGSGPLGEVLASA
Vc     869 :-------RRLDPDTGRVFHLEFDPPPANDPGLSARLKEVADVSNDAQQIQHRLASQSELAATLDDWLRRFSRLRRPVDGTGPLAEVLASA
Gp     837 :-------RRLDPTTGRVYHLEFDPPPANDPGLSARLKEVRDESNDAQQIQHRLAAQAELAGPLDDWLRRFSRLRRPVDGSAPLGEVLASA
Hs     792 :---------DIIAEELSYKTAHEDISQ---RVAAENQDKDGDQNLRDQIQHRIIGFLDNWPLLEQWFSEPENILIKIN------------
Xl     799 :---------SKICEID--KSGEKCDSP---RIQTTRIKED---ETMDQIQHRITGFLDNWKNLEMWFSEQLNILIKVN------------
Dr     783 :EGTQIVNGESIKQEKGSSDAAEENTSMPDLNTQDPESLSDDKSLGRTQIQFRISGFHQTWPKLEKWFGDQQNILVKVT------------
Ci     716 :----RTYGTESGETFHTEYEPPPFGSHSGLGKVDQIKNVQDPAFEHEQVQHRITSFHSNWEKMEKLYS-EITTVQHVD------------
Cc     750 :---------NSKLLPNPLPRQVDIHL--KTFMTTYIKAESNLKHGNVEDNEIFEILPEDSTPVDTFYTNEGIAYILYY------------
Oa     733 :---------QSRLLPNPVPRLAGLAP--KSLVSSFVRVLPKPEDVSFDERDLFEVLPEDATAMDAFYAGQGTAYVLYY------------
Wa     799 :---------HSKLLPNPISKAKDYSTPSTTFMTMYIKAVPKPKAIDSQE-QPCIPLPDDATSMDGYYANQNIAYGFFY------------
Tt2B   854 :--------RLDPQTGNIYNLEDNLPPLEEKGLADRIQPIDDPQSSLEKIQEANKQFIENLDYLQEWYGMFGFETEPASN-----------

                       1180      1190      1200      1210      1220      1230      1240      1250      1260
Tt2A   953 :---TLNEEEHPLSDQQQSDSRQGTLENNEQVNNQHQAGSMNQNLEENAQNVQDSQPEEQVEN------------------GETQNQLVPQ
Im     895 :-------QYYNSYENNQSDNQEGYEINEQEEQNQYEQ----ENLDHDINHINSQNKQSELKQ------------------EQQGQNNSPN
Pt     830 :----------NNFESADNSNAQSAFIGVDASNN-------YERVFYDVDRIFQNFLQIQINK------------------IQQKYDNQQN
Sl    1006 :-------LNSKKGEARDFQILNKVSGDLDQDSLHKEIIQVIQKIQHKKSKQEVKIKKRILEK------------------IIQTEIEEAE
Ot    1011 :-------ASSKKGEINDLHILSKVNGDLTQDELHQEILQIIGRIQHKKGKQETKLKKRVLEK------------------IMKRESEESE
CPC1   925 :GDIAEGLLRAKAAAASCRSAAEAAHKARASAEQAHEFAELAQAAAESAARELLTAKKAEIQAAALLAGGKNPDPAATEVLKAQAAAKCAE
Vc     952 :SDIAEGLLRAKAAAASCRAAAEAAHKARASAEQAHEFAELAQSAAESAARELLAAKRAEIQATALLNSGKNPDPAATEVLKAQAAAKCAE
Gp     920 :NDVAEGLLRAKAAAASCRAAAEAAHKARASAEQAQEYAELAQGAAESAARELLTAKKAEIQAAALLAGGKNPDPAATEVLKAQAAAKCAE
Hs     858 :--------AEIDKESLCEKVKEILTTEIAKKKNKVEKKLEEKEAEKKAAASLAELPLPTP-PPAPPPEPEKEKEIHQSHVASKTPTAKGK
Xl     860 :--------GEVEVGVLCKKIEEVFLTALVNKQN------QGKETEKKEDLPPPEVATPPPAPPSPPPAPETTNNIRPPSASSKASAKKGK
Dr     861 :--------AGVDKDTLFSNVETILRDTIE-------------SVEKGAALADSRRASVTSTPEQTRPE---------SACSSKSSRATPK
Ci     789 :--------AEAAKDTVYAQLEKTVFSTLNKIQE--------KSEEEFAAELSDKLSEEIPKEEVKPQRSDLLRPDSASSRKSKDSIRSAR
Cc     817 :--------SALNLSTLKKLARLILGNKSVSEKSSVELFGDALDILVDR-KEDQGGRSYFAKQILPRFS-----DSVDGGESGDIYDEALA
Oa     800 :--------SVLDLPTLKRLARMVLGDPEFPPKPSEEIFRVPEYPLGGE-TEVLRSKRPISKRVLPRPESKESLDEVPGEMSPEESQRTLS
Wa     867 :--------NVFDLPTLKQLVKLIMDHFHE-RKSFLELSEKILQTYHDEQKCLIDSKAAVIKRLIPKSKWKHEEHEEKKDIKVESHDLQPE
Tt2B   925 :------LNTEQQQPQSQAQLQQNEDLAQEQQEQEKNIFGAIQSLESTTKRIEVFEQIDKKLSDLLKIKKKKIEEQIQNYQQEQERLQQQR

                       1270      1280      1290      1300      1310      1320      1330      1340      1350
Tt2A  1022 :KSKYNVQQDYQIVWQEVNGKDKIDEVFYQVDQIIDRVLIAKEQKYEAIQNKLDSIEERKKQDEEIARIKKENEAKWLFTTREEILQDLQN
Im     956 :KDKQ-----------------------------------YEKNKQEDIN--IENQEDKKQQ-------QQQQQQQQQYIVKKELICCHQ-
Pt     885 :---------------------------------------ELLHKEELVK---QQQEQAELD--------KICSYKQVQLDSLPLKQPSQQ
Sl    1071 :KQRIALEEE-----------------------------EERKRKEAEGGDQQ-PGEEIKKE-------EGKVDKPEPATDRIGLKQAAPD
Ot    1076 :NLRKQQEEE-----------------------------EERLRREAEGGDQLNQAEEQKKED-----ITVKVEKPEPATDRIGVLPPTFE
CPC1  1015 :QLKVARGAVADANGHAERAANSAAAASEAVDRAHKSLGDAEISAHAETEAAAAATEAEKAARGAQEAASKALAAKEAAAVAAAEAERFAA
Vc    1042 :QLKVARGAVTDANSHAERAATSAAAAAEAVERAHKSLGDAEVSAHAESEAAAAATEAEKAARGAQEAASKALAAKEAAAVAAAEAERLAA
Gp    1010 :QLKVARTAVTDANNHAERAANSASAAAEAVDRAHKSLGDAEVSAHAETEAAAAATEAEKAARGAQEAATKALAAKEAATIAAAEAERFAA
Hs     939 :PQSEAPHGKQESLQEGKGKKGETALKRKG----------SPKGKSSGGKVPVKKSPADSTDTSPVAIVPQ---PPKPGSEEWVYVNEPVP
Xl     936 :RGSKSPRESQKRSDTSKDKKDKKPETPKGK--------DSRKSGSPRGRSPGKKTKSIPASPEPVAVTPVGLPPIQPGSPQWVYVNEPLP
Dr     921 :SARRS------------------------------------------------------------------------------FAEEPLP
Ci     863 :SKTKSGKGSARDRKKSGEKSDKKSRS---------------RSASSKSSKDKRKSATPPPAPVAEAVEPEEVNKPKPGDQGWEYVDEELP
Cc     893 :SIQDFDKS-----------------------------------------------------------------PAEPGEDHWEWIDFPQP
Oa     881 :--REVEPE-----------------------------------------------------------------RIDPGDPGWKWAAPPSS
Wa     948 :AQTQLTND-----------------------------------------------------------------YAKPGESSWQWLDFFQP
Tt2B  1009 :EEERLLQEKLEQERLEKEKQQQQQLQEASN-------SANTQSNNPNASASQEQLSSQNKIPTDNLISNDMADTSKINQTDQSMQNSGQA
```

```
                 1360      1370      1380      1390      1400      1410      1420      1430      1440
Tt2A 1112 :FSASIK----------------------------------------------------INPTALKNLVNLWKNTQDIYINSSKFVFYNLR
Im   1001 :--------------------------------------------------------------AITNLANLWKNTQDYYINITQDVFYDLQ
Pt    925 :--------------------------------------------------------------FLQYLHSFWNQIETKYVS-LQNIFKGFR
Sl   1124 :N---------------------------------------------------------IDNDFKPVIMDAWQQLCQNYKQQMKKVFRQVR
Ot   1132 :N---------------------------------------------------------IDIDFKPVILEAWQKLCTNYKNQMKKVFRQVR
CPC1 1105 :ASDLPADATVNLGHRPDAHATGAHAVSTSGGAPAS-------GNGTASGEAAAPAPEPLVRELAASLHEEWKTLETGYLEGLALGFASLA
Vc   1132 :ATELPSESSAAAVSRPTTVPSGATTVAATEGAAAATAVGADGAEATAAAEVAAPQPPVLPRPLAGSLLSEWQTVEQCYLDGLSLGFTSLA
Gp   1100 :AADVPADAAVNFNKSTSRAGTTAGDNAASGAAGAVPATP---GSVVEAAEPPAPTAPLLPRELAAGLYAEWQTLEKGYLEGLAFGFASLA
Hs   1016 :E------------------------------------------------------------EMPLFLVPYWELIENSYINTIKTVLRHLR
Xl   1018 :K------------------------------------------------------------EIPAFLVPYWTTIENVYGTTIKTALRSLR
Dr    933 :K------------------------------------------------------------ELAEYLVPYWENFCSSYVTNVKTVMQNLR
Ci    938 :L------------------------------------------------------------TLVHSSVPYWKNAEMTYIKSCKFVFREIR
Cc    918 :P------------------------------------------------------------ELLEAMATLWENMENVYIQDLKDVFFIKR
Oa    904 :P------------------------------------------------------------RLLESLAKIWEAAEEVYVEDLKSIFFVKR
Wa    973 :P------------------------------------------------------------ALLEILATLWENVEQAYIEHLKEIFFLKR
Tt2B 1092 :GGDFFDR---------------------------------------------------LKMDNLKTVMKMWNEIQTNYFKEIKQSLHKID

                 1450      1460      1470      1480      1490      1500      1510      1520      1530
Tt2A 1150 :EQKQSILLRFHDIQKRFLELIQRPDQKFMLMRQFQQEYNKFVELNPDMCEEDQTKEELHQRVEDLHDQLYDIIEAKRDEASEERNQIIKS
Im   1029 :EQKELIIVRFNDIQKKFLKLIQRQDNKFEYVRQYQEQYNDFVQNYPDMCEEQQTKEELHQRVEDLHNLLYDIIENKREEATEERNQIIKS
Pt    952 :EQREFLTNYLMDIQRKFIDLINTPDEKFYHVRNFQEQYNQFVQENPDLCEEDVCKEELHQRVDDLYDQLIDIIDQKRDDLLVEKQQIQNS
Sl   1157 :DQRERLTENFSTIQNQFLKFLHRPDQKQEKLDQFIKEFNEFSDQYPDLREDEQTKDELHQRVDILSDELWEIIEERKEQHIEERKKIMES
Ot   1165 :GQRERLIDHFSELQNAFLKFLHRPDQKQEKLEQFIRDFNEFSDLYPDLREDEQTKDELHQRVDILSDELWEIIEERKEQHVEERKKIMES
CPC1 1188 :EQHAEAQRHFAGLRGRFQDLLQRPDEKPALVARFLTTFN---AVEADLRGNKEARGELLLRAEELRDALWALCDKKMEEAEAQRAAVASD
Vc   1222 :EQHAAAQRHFDALRQNFRDLLQRPDEKPALVARFLTAFN---AVEADLRGTRETRGELMLRCEELRDQLWALCDRKMEEAEAQRAAVAAD
Gp   1187 :EQHAAAQRHFETLRERFRELLQRPDEKPALVAKFLTAFN---AVEPDLRGSKEARGELLLRCEELRDALWALCDRKMEEAEAQRSAVAAD
Hs   1046 :EDQHTVLAYLYEIRTSFQEFLKRPDHKQDFVAQWQADFN---SLPDDLWDDEETKAELHQRVNDLRDRLWDICDARKEEAEQERLDIINE
Xl   1048 :QEHLTVIHYLYDIRINLKDHLKRPDHKQEFVSQWQSDFN---STTEDLWEDEETRAELHQRVDDLRDRLWDICDNRKEEAEQERTDIMND
Dr    963 :GERELIIHHLYNIRENFRQYLQKPDLKQEFVSSWQRDYN---SVPDNIREDEETKGELHQRLDDLRERLWDISDKRKDEAEQEKAGVIHD
Ci    968 :MERESIIRYLYEIRQDFQLHLRRPDHKQTFVTAWHRDYN---SVAEDLRKDEETQAELHQRLDDLREKLWDICDTRKEEVEVERSNLISE
Cc    948 :IHYAAIAPYQSEVTKHMTDYIKRPDNKQNILHEFHKAYN---AVEMDVRDDPDVMGELHCRVIDCQSDLWQICDDRRNEAENERKKLITD
Oa    934 :INHSTVVPYKDFVKKHMKEFVERPDNKQDLLHEFHVRFN---AFQEDVRQDEEVKCELHCRVGEFQDILWEVCDRRRKEAEEERRRIVLE
Wa   1003 :MHISAIMPYKNLVFRNLMKFVDRPDKRQILLQDFHRAFN---EIDKDLREDLDMKCELHCRVDDFRTELWELCDARRYEAEEERRRTLCN
Tt2B 1131 :NHREVISNRLADVQFRFLSVIQRPDDKQHKINTFKDNLNKFFDDNPQMLRFQNTKDELNQHLETLYENLWDSVEQKKAESIQERKTIMAS

                 1540      1550      1560      1570      1580      1590      1600      1610      1620
Tt2A 1240 :GWIEKEIEKFMLLIQQLLQAELSRSFSCQQIINDYYVLIQG--------------GELQEIPE-----------------YFKNDIVQNQ
Im   1119 :GWIQKEMEKFTLLMQRLIQAELSRSFSCQQLIADYYTLVLG--------------EELQEIPE-----------------YYKNDIISNQ
Pt   1042 :QFIENEIDLFLLQVRQLLAIELDRAQNSIQLLNDYYGTLEA--------------IELQDIQP-----------------SVG-------
Sl   1247 :GWVEYELTFAVSSAQLLMQSEVDKFKASVQLLHDYYHAFED--------------KLIPEAPQ-----------------FFTQDLVADG
Ot   1255 :GWIEYEQGFIVNSAQLLMQAEVDKFKSSVQIIHDYYHAFED--------------KLIPEAPP-----------------QISTEIVADG
CPC1 1275 :TFVPDHCATLAQQFVALAQVEGDRFAAALNFLRSHAHSKWLPLFPPGREPPSLALPDVAAPDL-----------------LSGAVPPELK
Vc   1309 :SFVADHCSLLAQQFVALAQVEADRFAASLNFLRSYANAKWAPLFAP-REPP--MLADVSAPDL-----------------LSGGVPLELK
Gp   1274 :SFVADHSGTLSQQFVALAQVEGDRFAASLNFLHSTVQARWAPLLGAREPLP---LADVAAPDL-----------------LSGTVPHELK
Hs   1133 :SWLQDTLGMTMNHFFSLMQAELNRFQDTKRLLQDYYWGMESKIPVEDNKR--FTRIPLVQLD---------------SKDNSESQLRIPL
Xl   1135 :GWLQDHTGILINHFFSLMQAEADRFQDTMRLLSDYYHGMEGQVPSEAGHN--FARIPLLDIAN------------TSTQGDSENRKRIPL
Dr   1050 :GWLEDHTAVLINNYSALMQIEVGRFQDCVCLLKDYYSTMG-KTAFESKCD--FARVPLLDITA------------DQHVDESKISESAPS
Ci   1055 :GWLQDHLGILTNLYLTLMQGEIDRFEDTARVVKDYYSGMRKSIPGENVLPGMLPRLPLVEHDVGVHGVPTVQSTLTVGSENPSISPQSSQ
Cc   1035 :NWTASEIVILSNVYISIIQAEINRFLDTMELLHDYYMSMLQRPINEYRLP--KVVLHRYTSTS----------------EMDSGKKKESK
Oa   1021 :NWTLQEAIVLGNLFISMVQAELDRHVDTMQLVQDYYSSSMDRPLSQNRFK--KILLPRIDLQEK--------DPEERSLPASPAKSGKGK
Wa   1090 :QWILMKALVLVNVYIGILQTEIDRFVDTMQLLQDYYTSMLQKPLQESPFS--KIVLDTFELED---------------VLQETSLEDKER
Tt2B 1221 :GWFDSEMAALFSSYQRFFQLELNKLEEIIELILYIQDVVEG----------------------------------------KPINEVQEH

                 1630      1640      1650      1660      1670      1680      1690      1700      1710
Tt2A 1299 :ESLPPIE-----------ENNNFPRLEKLVDLTLKLHQGEE-----------------------------EVEEKPGAKKGAAKAPPKKD
Im   1178 :DSLPPVE-----------DAQSFPRIEKIIDSALKLHNGEE-----------------------------EQEEKGANKKGGKVA--KKE
Pt   1094 :-ELQLQD-----------GDDPNVRLDKLIADSLRIFAGEE-----------------------------EIIEDKTKKGGKQPA--KKD
Sl   1306 :EELPPVESLPEGADSSNIDQYQYPRLDKLFEKALKTQVVQD-----------------------------VTQLSSQNQDKKGAPPKGKD
Ot   1314 :EELPPVESLAEGSDIANAELYQYPRLDKLFERALKCQFVPD-----------------------------VTQIQAQTLDKKGAPVKGKD
CPC1 1348 :DKIDPKAKTSVPMPAWVASLEQRAAPLALATKLLLALAKTT-----------------------------ATSWEPGAEDPKKAKKDAAA
Vc   1379 :DKIDPKSKTAVAMPPWIAQLEQRAPPLALACKLIIALAKTT-----------------------------YASWEPAPDDPKKAKRDAGA
Gp   1344 :DRMDPKSKLAVPMPVWIPGVEARAPSLALAIKMLVALGRST-----------------------------RAAWEAPGDDPKKARREATT
Hs   1206 :VPRISISLETVT-----PKPKTKSVLKGKMDNSLENVESNF-----------------------------EADEKLVMDTWQQASLAVSH
Xl   1211 :VPRRAQSPEQNT-----GKQKGKSQSKAKEEPPMDSFGQLS-----------------------------EADEKLITDTCQTAVSAIAN
Dr   1125 :EKSVKNAEEKDSEIVDRMKTKVFPLVSCRSPTSEILKQPLH-----------------------------QADENLLQEFFQAAIAIFRS
Ci   1145 :SDTKKLSTKQSEDVTNENEATKLAQAGSRTKIPLIQRGVAFSNQLTEDGGKGKKGKPPAAVGTDSPVQDDKLEEKTVEEAFTFAANAIAH
Cc   1107 :ETRETKTKTSIGKDSAPSAIS-VNQVKNEIANILIDCEKNN----------------------------LDLDRVSCYEIIRANFRYVQR
Oa   1101 :ESRGTKKGSSGKKDKKSSAPAGLENVKDEVLDLLMSTDREE----------------------------EGHDQNGLYRAVHSCVQKARH
Wa   1163 :MVSETKTESS---AEASALVVDKSQLKTELETLLIDVSKS-----------------------------FDPDQSTVYIIIKDNVRQVRD
Tt2B 1271 :IILDPFPNGQPPPIYDIENPNHFPRVEKMFDHFMHSVDNIS-----------------------------ELIGDIRAKFFKKRTAGLSF

                 1720      1730      1740      1750      1760      1770      1780      1790      1800
Tt2A 1349 :DKKPPVKKGGKNAVEEVEE----------------------------------------------------------------KKELTPI
Im   1226 :DKKAPKKTG-KNAQEEVEE----------------------------------------------------------------KKELNPL
Pt   1141 :EKKG------KKGKEEEIV----------------------------------------------------------------KKELQ--
Sl   1367 :PKKP----------AEEEK----------------------------------------------------------------PVEDSQY
Ot   1375 :PKKP----------AEEEK----------------------------------------------------------------AVEESQY
CPC1 1409 :KAGAGAKGKGKGGEEEAPEA--------------------------------------------------------------DPRVVEGL
Vc   1440 :KVAS--KGKGKGADEDDAAT--------------------------------------------------------------DPRVVEAM
Gp   1405 :VVKGARIRGRNGGEDEPEGP--------------------------------------------------------------DPRVVEAM
Hs   1262 :MVAAEIHQRLMEEEKENQPADPKEK--------SPQMGANKKVKKEPPKKKQEDKKPKGKSPPMAEATPVIVTTEEIAEIKRKNELRVKI
Xl   1267 :MVSSEKQSKESEEEKERQMMEMKEKERVKVS--QAASRASGKGGKKKPPPKSPNRKKEAKSPGPADPTPSLQSLEDSTELQKKQELKMRL
Dr   1186 :MVSAETRQWDEEEVDEDQ----------------KQMEAQKAQTLTSATDVRKAGKQKG-APPPPPPEPSPQPAARNPEELRKRAEKKRF
Ci   1235 :VVQVCINAKEAEEEEERLQKEEAEKAKAALIPVDKKGGGGKKGGGKSAKGKKGGKGGKSKSPTPTPPTPVE---EESEEEKKKRETAARL
Cc   1168 :TVESVSSAMLDSLKKEEM-----------------AANALSATTTRRDSRSRGHSKRTSTSLAHSDP-----------IMVNVTKRSPDL
Oa   1163 :LVDSIAETLKSQLKKEEA-----------------AAGQDSAFKGKVASKG---SGRDPNPGREDD--------------LRTLKRN--V
Wa   1221 :LVDSVSSVMLEMLKKEEK-----------------AVIAKIEIKGK--------GNASFDTTRPAG-------------------RNRDL
Tt2B 1332 :QTIN-------------------------------------------------------------------------------EKSYPGL
```

```
                 1810      1820      1830      1840      1850      1860      1870      1880      1890
Tt2A 1375 :EKEQKEAINMEKAIFRYRVQVIKDFALSKLKLMKAQSQLLYGKLQDWIDYTFKVENDAVNQMDAIFREYIEEEKKIQKEIQLNFVDIVIS
Im   1251 :EKNMKEALHIEKSIFRYRIQVLKDFSISRLKEMKALSSILYGKLEDWIEYTFKIENDAVNEMDYIFRQYIEEEKKIQKEIQLNYVDVIIS
Pt   1159 :NPEAISAITLEKQIFKDRVDAIKRYASQQVRTYRQACDQLYLKIDEWIYYTHQTEIKALDAISGLFRGYIERVERIQKELQLQFVDVIVS
Sl   1383 :VRDMRAAIKVEKSILRYRLTQIRNWTLKRLREMRQKAIKVYLKLEDWIQVSNKTENDAIDEMSIVIKRAIEDEKKIQDELRVKFMDFCVD
Ot   1391 :VKDMKSSIKIEKQILRFRLTQIRNWTLKKLKEMRSKAIKVYTKFEDWILVSNKTENDAVNEMCIVVKTAIEEEKKIQDELRLKFMDFCVD
CPC1 1437 :NGELLDGCGRELAILEARLQLLAERCLGQMDEMAALASGTVSRLGEWVRERYRSECAAVAALDKVCKAAAAAGQPLPHDLRLQDEFLLLD
Vc   1466 :TQELLTGCGREVAILEARLGLMAERCMGQMDEIYNLATGTAVKLGEWIRARYRAECAAVAALEKVSKAAAAGGEQLQHDLRLQDEYLLLD
Gp   1433 :NKELLEGCDRELAILEARLCLLADRCLGHMEEIASLATGTTAKLGEWIRARYRAECAAVAALDKFAKAAAAAGQPLPHDLKLQDEFLLVD
Hs   1344 :KEEHLAALQFEEIATQFRLELIKTKALALLEDLVTKVVDVYKLMEKWLGERYLNEMASTEKLTDVARYHIETSTKIQNELYLSQEDFFIN
Xl   1355 :KQEHLAALEYEEMAANSRLELIKTKALEVCQDIASRAAGAYKDMEMWIGERFLAEMSSVEKLIHIARHHIETGTKIKYELVLENTDFYIN
Dr   1259 :KQELSAALKPEENAVKQRLELVKAHGLRTVISLQHKAEQVYRNMEEWMKARYLAEMKSIDQLTDLVRQHIETGVQIRHELVLQSTDFCVD
Ci   1322 :KEEYFAVLQQEEAAAKDRLKLIKTRSIHTLSDLYGKSEMTFTDMNDWIGSRYLREMAAIDNLSSVVRQCIEEGVQLEYELLLEQDEFIIN
Cc   1230 :FKEWRSAMMYEVNRVKTKLKILEMVSRDDIGNLLKTMQSVFGDIYKMLVDRYKNEVQSVDQMATVFCYAIEQSIPLQNEMLLDGDKFIVR
Oa   1217 :ILEWRTAANFETNRVRLRLKILDLASRSDLNLLLNTMREMFREIHEEITSRYHREMKNVNELTSVFCFAIEEARPIQEEMLLDGDRFIVR
Wa   1267 :VEEWRYAVLYEIGRIRQRLDVLNAAARADVTFLLDTMRQTFHRVYDRIVERYKREIESINEMANVFCVAIEEKRPIQQELVLDDDQFVVR
Tt2B 1343 :NDLYNQVLETEKGICKYRGYILKFTLISRIKELKMIEVALYSKLDDWIIFGLKHENEVIYDAVRTLRQAIQSQNPVDTLVVSVKNISDTY

                 1900      1910      1920      1930      1940      1950      1960      1970      1980
Tt2A 1465 :HEILNFLTPPPIILPAKEPLYENK--FSISQLYIIIEELKSITNEN----NQIESRALLQLLTRKTLNQISKWD---LPEFWRNNTIKTY
Im   1341 :HQILNFLTPPPIILPAKETIFQHK--FSISQLYVIIEEIRSITNER----NLVESRELVQFLFRKTINKITSDV---LPSIWRKNTIYDY
Pt   1249 :HEVLNFLTPIPPPLTAREPLSRER--YSISQLYYLIEDLRAITSTH----LLIDIKQLALLLARSSYGVL-------------KYSPSQW
Sl   1473 :EKIMNYIEPPPEKLPALEEVRHDR--FSISQLSTLVNELEHLAKQHGNGSNNVPNKVIVDLFVRKLENSKNLGDEGSLPVEWTNYTEYDI
Ot   1481 :QKIMNFIEPPPDKLPALEEIRPER--FNISQLKTLVTELEQLAQQFGNGTIFIPNKVIVDLFVRKVENSKNIGDEGSLPEEWRNYTEYDI
CPC1 1527 :EGSLLVQEDAALPRPYPREGVPAGGLLSTKQLGALTAAFLSAAPSG-----YMRLQEAADMLCRLAAEGA-------LPEPWRGTSVASM
Vc   1556 :EGGLLVDAPPPLPRPEPREAQPPGGLLSTKQLGAVTAAFLSAAPSG-----FMRLQDAAEMLCRLAAEGA-------LPEPWRSASVSSM
Gp   1523 :EGGLLVEAAPKLPRPQPREAQPPGGLLSTKQLGALTAAFLAAAPSG-----FMRLQEAADMLCRLAAEGS-------LPEPWRAASVGAM
Hs   1434 :GNIKVFPDPPPSIRPPPVEKEEDG-TLTIEQLDSLRDQFLDMAPKG-----IIGNKAFTDILIDLVTLNLGTN---NFPSNWMHLTQPEL
Xl   1445 :SDIQVVPDPPPPSRPLAVEISANA-TLTISQLSRLQQQFAQIAPEG-----LISNKTFIDILLDLTSMSLGND---ALPDLWMQLTLPEI
Dr   1349 :GDTRVLLSPELTPRRSLLEQPKNS-TLTVQQLHVLCTQLRKIAPKG-----LMCRSELTEALHELTSAHMGTD---VLPEAWMHITPSQV
Ci   1412 :HDAKVIRTPSPPPRPEPIETSQPD-VFTIVQLGKLYSHFKGVAPNG-----MILNKAFIEVLADTAALDAGAD---ALPDMWINISYNQL
Cc   1320 :LNVLLFPDKEELSAMTTPERISRL-QFGIIQLGRLLCILRYTAPNG-----SLMERHFVYLIQDMIAC-QEDCTSSPLPLSWFEMEPNDV
Oa   1307 :SNVLMFPDKPRPPAEPVLEENHVF-RFRMAQLGRLMDVFKNVAPGG-----KMSERAFSYILMDLVARGLEEGEVVPLPGSWYQLDPEDV
Wa   1357 :PNILMFPEAHEL-AVPIKEALSPL-RFRIVQLGRLTDIFQRIAPRG-----IVSERVLIYVLQDLVACGEEDCYPPFVPCAWRQLRPPDI
Tt2B 1433 :KILNCFNQIPAYIQPLELVESVDDFRFTIFGIKLLYEELHTLCDKR----DIINLKIFENVFLRRKKHSN------HLPKFIKQLPDKCL

                 1990      2000      2010      2020      2030      2040      2050      2060      2070
Tt2A 1546 :QKLIQNLDPNI-TGYINSKDICTYICLSGSHIPTQTEEEGYFEKLKLN----SSGDDMIEKAQFINTESWFDEIESCKLEI-DTYLFDRP
Im   1422 :QRLVLNLDPQK-TGYVNSSHLCVYLCLLGATLPSNQELQIYAQNLVFA----ANGEHQIEKNPFMFTEAWFDNQESCEIEQ-YSKQFDRP
Pt   1320 :MQALQILDQN---GYVNVRQICTYLILLSSPTPKEDELQQYFSKL---------GQPLVNKDTFVNTAAWFDEYERIPEEE-NTNYFDRV
Sl   1561 :QNMARNLDKQC-TGSIDWRQIATYIILLKSSIPTDKHLETYRAEFK-------GKDDLHNKQNFLQAQAWFDETEYSQDRD-YSIPFPRV
Ot   1569 :QQMTRNLDKYH-QGHIDWKQLATFIILLRSSIPTDTQLNEFKTALK-------TKEDSISLDLLQQTPCWFDEVEYSRDRD-YSIPFPRV
CPC1 1605 :LGALRLYDPLH-STYLDWREFVAHLVVASFPLIARADCAEMADQIDICAEVDKDHDGLLSQEEWSTAELWFQYRAAQPGALQAEVPEASG
Vc   1634 :LGALRLYDPLY-STYLDWREVVAHLVVAAFPLIARADCAEIADQVDICAEVDKDKDGLLSQEEWSAAELWFQYRAAQPGAAPSSAAPASA
Gp   1601 :LGALRLYDPLF-SSYLDWREFVAHLIVAAFPLIAKADCAEMADQIEICSEVDKDKDGLLTQEEWTAAEMWFQYRAAQPEEDAPAVGPSEP
Hs   1515 :QELTSLLTVN--SEFVDWRKFLLVTSMPWPIPLEEELLETLQKFKAVD----KEQLGTITFEQYMQAGLWFTGDEDIKIPENPLEPLPFN
Xl   1526 :KEITSELSQS--SDVVNWRLFLLSASLPWPYPSVTQLLNALLSFKALD----LNGCGTVTQEGYSQVELWFHGNVEEPDPENPTEPFPFN
Dr   1430 :EELVCILAQD--SEMLDWRQFLLSAALPWPFPTQTQLIKTLQRYRDID----TAETGLITQEQYEQVELWFPSEQDPSVPDDPTEPVPYD
Ci   1493 :VELADLLSGE--SEYIDWRKFLLLAAFPWQQPTTEELLLARSAFQQHD----VCGNGWISKDQFLNVKLWLDDSESSKDPD------VYD
Cc   1403 :ENLIFEIFGN--TEYIDWREFIIYA-MDIPIPTHEEILYARDSFRKMD----SELREVVTRDQFYSVPLWFLESTNIPRDSRFLLHDEME
Oa   1391 :EVLVLRLFGD--ETRLDWRDFVIYA-MELPMPSANQILDARDAFRVAD----PEEKELVSREQFLSTPLWFYTCSDAAEVSRCILHGEFQ
Wa   1440 :EILIERLFGA--AEYIEWREFILYA-MDLPVPSHEDILKARAAFRMHD----PESREVVTCEQFHSTALWFLEISTLYKS----LTDEKL
Tt2B 1513 :ESIYANFESQIHDDKIDFKDLMICFILFSSPVPTEDLIEEYKDQLLTY-----SNSGYLETHNFLRIEAWFDEYERDRQLYG--------

                 2080      2090      2100      2110      2120      2130      2140      2150      2160
Tt2A 1630 :RY----------------------------------------------------------------------------IKELLFYIHKDL
Im   1506 :KC----------------------------------------------------------------------------LKELMFYVYKDF
Pt   1397 :IY----------------------------------------------------------------------------IKELLFFVHKD-
Sl   1642 :HL----------------------------------------------------------------------------IKDLLFRVNKTV
Ot   1650 :KL----------------------------------------------------------------------------IKELLFKVHKKQ
CPC1 1694 :EAG-------------------AEAGTGSGSESD---AGPRSTLLADGEKWPEA---DTVSRAGTPPPDDEQYDSPAALKQVLWALFSAP
Vc   1723 :AGEPASESHRERSELAEAQQLSSSGGKGTGASGN---GGSGGHTRGDGESWPDP---SAIPPPSTPPVDDGQYDSPGALKQVLWALFSSP
Gp   1690 :SEQ------------------APEGRKQEGNEGEPATASSSSHSRKDGEVWVDPNLALVPAQASTPPIDDGKYDSPAALKQVLWALFSVP
Hs   1599 :RQE---------------------------------------------------------------------------HLIEFFFRLFAD
Xl   1610 :RPG---------------------------------------------------------------------------HLIKHFFDMFAD
Dr   1514 :RLA---------------------------------------------------------------------------NLKKFFFSLFAS
Ci   1571 :RNK---------------------------------------------------------------------------HFSEFCFTLFSD
Cc   1486 :QDEEEMYEEELFFNTSSAMST----------FNKQETARKSVIDWSTRSKGHLTVKDRLESNRLFCDDPDDPKPETINRYILAKKLLCQM
Oa   1474 :RDSQDLYPEEVGPEEGEGLS--------------------RVVGLDS------EVLQRLAR-RLRCD--EDAALREVLRLLLAKEVLCDM
Wa   1519 :DHSNSDTIKNIMLRQEAQLG-------------------ERVSSLKSDFIER-----------------SEDESSTILRLMLAKELLCRM
Tt2B 1590 :-------------------------------------------------------------------------------QNLFNRVFFVK

                 2170      2180      2190      2200      2210      2220      2230      2240      2250
Tt2A 1644 :---ETDFIDIDS---FIQTISASHYIQLRENEQITTYTDLLLS-----------------------------------------------
Im   1520 :---ETDLVDINQ---YIQALSALNYIHGHLNENIKTYNDVLLS-----------------------------------------------
Pt   1410 :---ENDMLATKQ---YIDILNV--EGDRFEDYLLQTLQHQ--------------------------------------------------
Sl   1656 :KESGEECISLKK---YFKILKSKELTMFKRN--IKIYSDILITQLSDQ------------------------------------------
Ot   1664 :LDSGEEVVSLKK---YLKVLKSKEITMVKRN--IKVYSDILITPPAAEQ-----------------------------------------
CPC1 1759 :AADGTPRLDYRACLLYLCADRDAFAGIKKAFSVATANISNNARASADQVFRIAYPLGAEPGQELHRSPFSKVDVAAVVKA----------
Vc   1807 :SADGTPRLDYRACLLYLCADRDAFAGIKKAFSVVTANISSNARANAEQVLRIAYPLGAEAGIELHRSPFSHEEVAAVVRA----------
Gp   1762 :GPDGTPRLDFRACLLYLCADRDAFAGIKKAFSVMAANISPSAKANADQVFRIAYPLGAEAAEELHRSPFSPDEVASVVRA----------
Hs   1614 :YEKDPPQLDYTQMLLYFACHPDTVEGVYRALSVAVGTHVFQQVKASIPSAEKTSSTDAGPAEEFPEPEENAAREERKLKD----------
Xl   1625 :HRRNPAQLDYTEMLLYFASHSDTMEGFYRALSVTAGKPILR------------TSGNHVLLESLPAIDAMQQQMKEPINN----------
Dr   1529 :THSSPLLLDYLMMLLHFCCHPEAAQGFTRALGLVTGHTLHY-------------KHTTPLTQSVPCLEGAGVVDDAGADN----------
Ci   1586 :WEQTPPRLDYVNMLMYFAMDKNTVTGFLRALSVASGKAMPS--------------YTKKLIEPTEDLNKNSVPGTERLLE----------
Cc   1566 :YLTNAYTTNYTAFLLAFCKSDDPRDGFGKALTLAMGGRVCIDEVEGESYVSHLLEQRRLQALEDARRNRLREEAVKVTED-----IVMAT
Oa   1535 :YSVDGNFVNYTEVLLAFCKDDDPKRGLGKAFSLALGNPLCTDDEEGERYVERLLLEKRLLAEVEACRNRLHEEALEVSLKSVGGAIVEQI
Wa   1573 :YLVNPDSVHYTAMLLAFCKDENPSEGLGKAFTLAMGAKVCTDVVKGERYVEQLVKQKRCAKELKLTRNYLRKEANEVVRE-----IVDYI
Tt2B 1601 :QLLFDLYCENEPYILSINQYIESLKRLSSKGKEYFWQILFE-------------------------------------------------
```

```
                 2260      2270      2280      2290      2300      2310      2320      2330      2340
Tt2A    - :------------------------------------------------------------------------------------------
Im      - :------------------------------------------------------------------------------------------
Pt      - :------------------------------------------------------------------------------------------
Sl      - :------------------------------------------------------------------------------------------
Ot      - :------------------------------------------------------------------------------------------
CPC1 1839 :---------------------------------------------------------------------------------------VWE
Vc   1887 :---------------------------------------------------------------------------------------VWE
Gp   1842 :---------------------------------------------------------------------------------------VWE
Hs   1694 :-------------------------------------------------------------------------------------DTEKR
Xl   1693 :-------------------------------------------------------------------------------------NDKVW
Dr   1596 :-------------------------------------------------------------------------------------EEEQH
Ci   1652 :-------------------------------------------------------------------------------------LSPSK
Cc   1651 :LDEVVRFCEMKGLKDQIQEDEYLEF-FRGPLIIESLDEPGTTDTSPAVDILKQLELSSDNSSTEKDIDSFRMTDSLLFTSSTLTANLIST
Oa   1625 :VEAVVQEVEGLLLKELDKLRLEPDFGYKRPILIQSLDEPRVVDP---AELLADQDVGIPEEVSGGDLEESSLSVEPESTSSMSSTEIEAS
Wa   1658 :VNKATEIVAVFERSRNDYLN------RKRRIMIQQLNGSGEIDP---AELLPPEEIAEHLVVD-----NPSDSDEHEHKQSLVLIENETL
Tt2B    - :------------------------------------------------------------------------------------------

                 2350      2360      2370      2380      2390      2400      2410      2420      2430
Tt2A    - :------------------------------------------------------------------------------------------
Im      - :------------------------------------------------------------------------------------------
Pt      - :------------------------------------------------------------------------------------------
Sl      - :------------------------------------------------------------------------------------------
Ot      - :------------------------------------------------------------------------------------------
CPC1 1842 :SRAGQPAVAAGAAGTGPAGARGATPPQAATAAARGASAKNTAGAAAPAASGEASVTAEQLMYSAGGERFVKRMLERYAWKDAFVATRL--
Vc   1890 :SRNPPKPPAP------SAGSRGATPPSGVGRAG--------GPTAAAATAGEPTVTAEQLMYSAGGERFVKRMLERYAWKDSFVATRL--
Gp   1845 :SRNAPHTPDSAP----GTSAKGTAAQRAAAAAAAAAS-----SAATAASAADATVTAEQLMYSAAGERFVKRMLERYAWKDAFVATRL--
Hs   1699 :EQKDEEIPENANNEKMSMETLLKVFK-GGSEAQDSNRFASHLKIENIYAEGFIK-TFQDLGAKNLEPIEVAVLLKHPFIQDLISNYSDYK
Xl   1698 :AQ-------DSGKASVTLDEVVRVFKHAASKDEDNHRFCPEENDRNNMYKSISH-IFAELAAEKQQCVAVATLMSHPAFQELLGNWRIYK
Dr   1601 :RG------VESEEVGVSVDDVLRVLSHGEDLVLHLNRFQLVHRSRDELREELLK-VFKDLGFSGEEKIPFSTLSQHPFLQDLIEGSAYYL
Ci   1657 :TRGIKLELDTNPSPEISAQDWWSVLHHGSSRLGDSHRFAEFDDPKSQFSMPQLQSLYEELESDESSTLPMQTLILHPVLQDAILGCKKYQ
Cc   1740 :DKLDG----RHVLHWVPRETCQAVLLASLPWHARQPNLYEVTGSLQESLKQVYLNLYDKELNEEKDVALAHRLVNHPFIAKMLSSTSKFT
Oa   1712 :RSLQHVETQRDSVFWIHRKICIAVLATLLPHLAVQ-DYFQSSRTLRGQIDLVYQDLKDEELNDDEDLVLSHRLVNHSFIKQLLANTGKFG
Wa   1734 :KDHKRVQQDERVIFWLPRNVCLTVLSTCLPWLASQENLFETTLSLHEGIARVYDELRDEELNEDKDLALAHRLVSHSFICELLRASSKFT
Tt2B    - :------------------------------------------------------------------------------------------

                 2440      2450      2460
Tt2A    - :---------------------------------
Im      - :---------------------------------
Pt      - :---------------------------------
Sl      - :---------------------------------
Ot      - :---------------------------------
CPC1    - :---------------------------------
Vc      - :---------------------------------
Gp      - :---------------------------------
Hs   1787 :FP-VHIIYVVEMIVCAKGWAPLPSGKQCARWRN
Xl   1780 :FPDIHRILNKQKAPQASDGENLPASSR------
Dr   1684 :LHDIHKIFQTKQTEDSRSFST------------
Ci   1747 :FLDIRSVFHGENSAYDADTTQFI----------
Cc   1826 :TKNMARVLDKLLQERDERKNASKITLTR-----
Oa   1801 :NKNIGGILREVLQQKERTKRTLET---------
Wa   1824 :SKNMAKLLRSILKDEDGAERRDR----------
Tt2B    - :---------------------------------
```

(b) Cfap69

```
                     10         20         30         40         50         60         70         80         90
Hs     1 : MWTEEAGATAEAQESGIRNKSSSSSQIPVVGVVTEDDEAQ---------DVFKPMDLNRVIKLLEETDKDGIEEKQLKFVKKLVQCYQN-
Xt     1 : ---MELGTDSLAG--HLNRDSRQRAALRKSEMSSDKPDSA---------LTFKSMDLNRVIKLFEDPFSREITERQVRVLRKVAKHYCN-
Bf     1 : --MAAATATQGLHRTTARMPKTP---IPIVNHIISDEAVG-----IQK-VVPRSSDLARVVKLLTDPYSENLIERHLHALTKLAAKYQR-
Ci     1 : -MAAVSSMRRQTPNRHTEMQNTEDKQIPVVAYIITDDEAG-----LTQGLKLRPVELTRVVRLLSDPHSSQLFERHVHALNRITLKYEY-
Sp     1 : ----------MTTKMATQLQPKP--PVPVVGHVITDEEMG-----LKKGIKIQQVNFVKVIKLLTDPHSVALYERHNHALQRLIRHYSA-
Spi    1 : ------------------MAAATARQIPVVNNVITDEDLNNFRTEGKQSGRLVPVSVVKTHKLLTDKHSREIVDRHVRAIHRLTKYYCH-
Tt     1 : --------------------MSTLG-NTQGNAVVD----------------FEAANKVLQMFESEYTEQLGDRKASSIIRFCKERSD-
Pp     1 : --------------------MTYNNQNTKSNLQKNQ---------------PLTAQSVLQMFESQYTEQLNDRKANAIVRFCRERRD-
Sl     1 : ---------------------------------------------------MDLSKVIHLFESKHTANLSDRHAAGIIQLCKEMVNE
Ot     1 : ---------------------------------------------------MDLSKVIHLFESKHTANLSDRHAAGIIQLCKEMVND
Ai     1 : ------MATVRNDTTGNALVMKAKPPAPTDGPVVEDTG--------------SAKDFQKILDTFQDKQTTELYSRQVALLQVLLQKFPI-
Cr     1 : ------------------------MDTATMTQSMGGAAAG------------ATVELEKLIALFTGPNSADIYDRHVAAMQRLCRNNAA-

                     100        110        120        130        140        150        160        170        180
Hs    81 : ----GLPLRDLAQIFKILNLCSGKIKN-QPRFIESAYDIIKLCGLPFLKKKVSDEITYAEDTANSIALLGDLMKIPS------------
Xt    76 : ----GIPLKDLSQIIQILNLCAERIED-HKDFVDPMCEIVKLCGLPFLKEKTSDELNYAQVVAESISQLGCMMRVPS------------
Bf    79 : ----GFLMRDLVLVFKVLNVCADRIPI-DPSYVEPMCQLLQICSYPFLKEKTSDETAYAQIVVESISQMGYLMRVPN------------
Ci    84 : ----GFLIRDLVHVFKILNICADRVAQ-HTCYVQPMCKLLHICGLSYLTEKMSDESAYSQIVIESVSQMGYLMRVPN------------
Sp    73 : ----GFLLKDLVQVFKILNVCADRSHE-QPMYIKPMIEMLKLCSLPFLREKSSDETTYSQIVIESFSQLGYLMRVPS------------
Spi   72 : ----GFLMKDVDLVCDMITVCRERLET-NLQYEKPLCDLIKVCGMPFLKERSSDDSLHAGPIINLLTELGHTMKINN------------
Tt    51 : ----GFYYQEIEALCKMVEYAIADLKKGVVEMTAAIQKLCETSSNPFKKTKASDELKYVPILP------QPLLYNTH--AEEKIILNEEV
Pp    53 : ----GFYYEELESLCKMLKYAINDLQQGIPEMAQSISKICEVASLPFKKTKASDELKYVPIIPEFLNCFKPLLYNTY--NETNEIYNQEI
Sl    37 : DQRKGFFYKDLPQVARVIELSFQGIQNGRFELVPAILQLLEVLKVPFEKEKASDENKQVPNLPILFNSLCPLLRYELPRDGEADIKLVQQ
Ot    37 : DQRRGFCYRDLPQVGRVIELSFTGIQNGKYELIQAILSILEVLKVPFEKEKASDENKQVPNMPILFNALCPILRYEIPRDEDADISIIQQ
Ai    70 : ----GFKLQNLPDVEAIIRIAYEKIAAGVDVLIEPVCSLVVHCSKPYLRIKSNEEFTSPQLLHNFVCLLGEFLVSFD------------
Cr    54 : ----GFAIRDLPKVQQVLELSVALLKRGSTGFVEPLCELVGTFSKPFIRRTATDEFKMLNHITSLLMTVGDIFRSPG------------M

                     190        200        210        220        230        240        250        260        270
Hs   153 : -SELRIQIC------------------------------------YQQASSSYKIQMAEVGGLAKTMVQSMTLLENQLVEK------
Xt   148 : -SQVRIQLCRALINFYNPDPV--------------------IQQVEGFQPTAVNYKVQMAEAGGLAETLILSMTLVENQLREK------
Bf   151 : -NNIRVQLCQSLVSFYCDADKYA------------------KSPLEGLKPSTKEYNIKVVEKSDVAETLVKSLALLESDLEVK------
Ci   156 : -SSVRSALADALISLYCNPPT-S------------------QDVYQDLKPASHEYNIRMVESSDVAETLVKSLSLFHNNLEIQ------
Sp   145 : -REVRIQLANTLEAIYTNNTK--------------------GLLVQDHQPTTRAYNAAMVERSDIAETLVKSLTLLENDLEVK------
Spi  144 : -VTAQLQLGESLLRFYTDQSFE-------------------NQSFEGYQASSLSHNITLVERSGVARALVESLEQVE-DLIVR------
Tt   129 : TESLRYSAIIFLRNFSDEGIDTILQAE----KNFEKAKMQGYEPSPLQKLLNEGSKNLRAINKSDVIEDIIFTMQYYQKNYEYL------
Pp   137 : TQTLRYYAINFLKSFSCENIDEIMQKE----KNFENKKMQGYEKSPLTQLMNDGTKNLRALLESEIVEDIIFTMQYYQKNYEYL------
Sl   127 : LDNICCEIANTVSAIASYGIQELKDQETNFQSSLEDKMIYGSETSPLQRLYQKGNRNLKGLSQSEMPETLVNILAENMKSKDTI------
Ot   127 : LDNVCCEIANTVSAIASYGIQELKDQE----ASLEDKMIYGNETSPMQRLYQNGNRNLKGLSLSEMPETLVNVLAENMKSKDTI------
Ai   143 : -PQIQVAAADTLRTFATGACLGHLPRQ-----------SANADEHSDDQRPLPRDYSQQLIERCGVAEAAAAALQRLLDDQQANDDHNRM
Cr   128 : PPQLVAVAAQTISTFANAYGNRPNALD------------LASQHVEAEGPQRQYLTNQGLLGKSGVVADVIVALARSALADREPPTSPSA

                     280        290        300        310        320        330        340        350        360
Hs   197 : --------------LWVLKVLQHLST-SEVNCTIMMKAQAASGICTHLNDPDPSGQLLFRSSEILWNLLEKSSKEEVIQQ----------
Xt   210 : --------------LSLLKVLQLLSS-SPENCKLMAKAQGANSICYRLSDPDPSGQLLFRSSEILWNLLENGPKEEIINQ----------
Bf   215 : --------------LNVLKVLQCFSVNSDSNCDQMLSAEAAYRICSRLNDPDPSGQLMFRCTECLWNLVERGDTQAVKRQ----------
Ci   219 : --------------LKILRVLQKLSCSSAVNCDKMLQSEAAYRICCEMNSPDPSGYLLFISVEIIWNLLELGGD-EVTKQ----------
Sp   207 : --------------LRLMIILQHFSSFSPINCNSMLSADAASRICSRMNDPDPSGKLLFHSIEIIWNLMENGDREEMARQ----------
Spi  206 : --------------LEIMKVLQCYST-SALNCDDMLSVGAAGLICSGINIPDPSDGVVFRTVEILWNLLEFGSKQEVVRQ----------
Tt   209 : --------------LPLLDLISNCIMFKDLAQKFCNFGILKDIIYVIFDCDDFRSYIVKSCFEIIWNAIEAVGISSIQLF----------
Pp   217 : --------------LPLLDLISSCILFRDLAQKFSNFGILKDIIYVIFDCEDFRSYIVKICFEIIWNAIEAVGISCIKLF----------
Sl   211 : --------------VAVIESIAHSALYVPNAQKFSQMGVLKDLVRIISEAQDFRSYIVHISIEAIWNLIEVDGQKTIESM----------
Ot   207 : --------------VAVIEAIAHSSLYVPNAQKFSQMGVLKDLIRIISEASDFRSYIVHISIEAIWNLIEVDGQKTIESM----------
Ai   221 : D----------LLLFPIVDLVQEISSWGPNAQILTAHGSHHHILDILDQIDDLRDVFLPLCLDILWNVLEISAATTATIQSCSSRAVMLD
Cr   206 : RGGAPDPAEQARALAAPLLTALLAVSYNAESCTAMVEAGVLQCIAALLG-RGPHADTSGATVELLWNLLEQAPALARAALSRGSVYEAAV

                     370        380        390        400        410        420        430        440        450
Hs   262 : -----------------------------LSNLECLLALKEVFKNLFMRGFSHYDRQLRNDILVITTIIAQN-PEAPMIECG--------
Xt   275 : -----------------------------LSNTECIYALKDSFVRLLTNGYKHYDRQLRNDLLVIATLIAEN-SVPPLIESG--------
Bf   281 : -----------------------------LNNLTCTSSLRDAFCQQLTKGHSHYDRQLRNDLLVLATLVAQS-KDTHFIETG--------
Ci   284 : -----------------------------LANLQCVNALKEAFRELMISGCSNYEKQLRNDLLVITTLLCQN-PSAPIVESG--------
Sp   273 : -----------------------------LNSFICVEALKDAFTQQMTNGYSHYDRQLRNDLLVLTTLAVANNPEAPFIETG--------
Spi  271 : -----------------------------LNCKECVSALRDAFVRQIKEGYSLADKQLRNDLLVMASLVLSLCPTSPFMETG--------
Tt   275 : -----------------------------AAED-IITQLKRVFEDLMRNGYKLEDKCLRNELLILINYLLSDEKALQYFDERSTMPNVSS
Pp   283 : -----------------------------AAED-IINSLKKIFENIMKHGYKLEDKQLRNEILILINYLMSDEKALYYFNSKTELPNQNE
Sl   277 : -----------------------------AGEQEVVLSLRRPFERVLKEGYKLDDRCLRNEICILINYVVTSMASHKFFLERETPND---
Ot   273 : -----------------------------AGEQEVVLSLRKPFERVLKEGYKLDDRCLRNEICILINYVVT-------------------
Ai   301 : ALRTKNAI------------------YAMGNLRSFQVLHKLLQRLLMNGYRKQDKELRNTTLMILDILASKSRNLPLFVESG-------
Cr   295 : SLRASSPGGASALDEFGEEPDIRPLRGGGGLAMALADALGGLLRELLLNGFSKADKELRNDCMLVMGLLLEDAAFAAAASYSG-------

                     460        470        480        490        500        510        520        530        540
Hs   314 : ----FTKDLILFATFNEVKSQN---------LLVKGLKLSNSYEDFELKKLLFNVIVILCKD---LPTVQLLIDGKVILALFTYVKKP-E
Xt   327 : ----FAKQLILFATFSEVKSHS---------PLVKSLKLLHNHEDFEMKKLLFNMIVVLSRD---ISAVQLFSDGKVMLALFNYVKAN-E
Bf   333 : ----YAKELVLFTTFSEVKSHN---------PLVRNLKLETNHEDFELKKLLMNIMVLLSRD---SAFIPIMSEGRLLLALFHYIKGN-E
Ci   336 : ----FAKQIVLFATFSEVKSYN---------PLLRNLKLTQSHEDFELKKMLINLLEILSKD---HAALQILSDGKVLLSLFHYVKSD-D
Sp   326 : ----YAKVLILFATFQEVRSHN---------ALVKYLKLGTSPEDFELKKMLINVLVVLSKE---PAVIPLLSEGHVLLALLSFIKNN-E
Spi  324 : ----FTKLIVLLATFTEVKSHH---------DLVRNLKLYQSHEDFEFKKLLMSTLVLLSAD---SGSSQIFCEGRVILSLFSFVRAN-D
Tt   335 : P-TTFIDTLLVYATVDEIEFYN-----DKILTNNHRAFYSTTSEDLEFKKLIWSGLLTAIQS-GNQSILETIKDSAFIYSLLLYIDPL-N
Pp   343 : TNPTFIDILLVYATVDEITFYN-----KLTINNNMRAFYGTTTEDLEFKKLIWSGLLTAIQS-GNSQIIETIQDSAFILSLLLYIDPL-A
Sl   335 : --STFLEAILFYSTHDELNSKVNFIGDGSSMSKTEKPLFTTRDEDVEFKKLLWTCVLYIVRDPDNHEAHQCLIERNFMQALLMYVDPN-N
Ot   315 : ------KAILHYSTHDELNSKVNFIGDGSSMSKTEKPLFTTRDEDVEFKKLLWTCVLYIVRDPENQEAHQSLIEKNFIQALLMYIDPN-N
Ai   365 : ----LLATLLKYAEAADLAPPP------STSVAKASNFASSCDEDFEFKHMLWIVLADVAKG--DLDAVRLISDSSFLHVLLLYVSPDQE
Cr   378 : ----VFEALIAAGTCPELGSAP---------ALVANYALSTDVLLHELRLLVWAALVQGCLR---PEVLEQAVASGLVRVLLLYVSPG-E
```

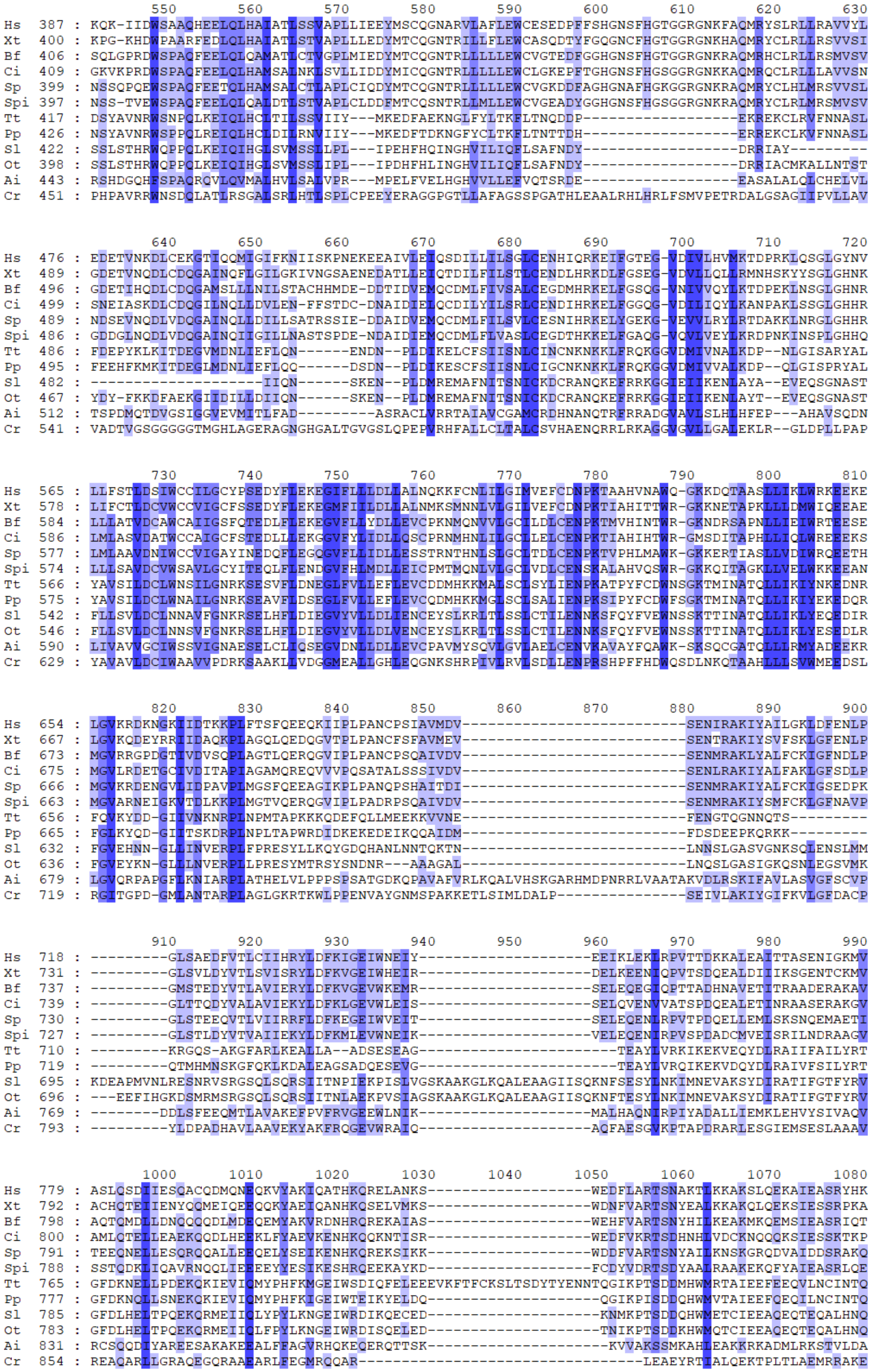

```
             1090       1100       1110       1120       1130       1140       1150       1160
Hs   850 : RPQNAI------FHQTHIKGLNTTVPSGGVVTVESTPARLVGGPLVDTDIALKKLPIRGGALQRVKAVKIVDAPKKSIPT
Xt   863 : KLNNSS------FHSTQISNLNTTVPCGQLVAVESTPSQLTGGPLADTAFALKRVPIRGGALQKVKSTKTLD--------
Bf   869 : RHQKEEK-----FHHTEVQNLNTTTFCGRSVKVNSTPVELTGARLKPYNIQRDMQ----KAVTLSDSATIIS--------
Ci   871 : RQGNTTKKLSTTFHDTTSKNLNTTTFQGRHVTVESTPAELTGERAQLLG-------------------------------
Sp   862 : IDNEHS------FHDIDLPSLQTTTFQGRTIMVDSTPRDILDG-STLITSHTDAE----NNLTLSKHASIAVS-------
Spi  859 : RVYPGEIS-----HDTLQEDLNTTTFCGRRVEIESVPLEITRTITTTTTADEEKK----TPLVA----------------
Tt   855 : S-----------LISREHKKSQEEELNKFYDIIRSNKVHK----------------------------------------
Pp   847 : S-----------LIAREHRKQQDDQLNKFYETIRNNKVTK----------------------------------------
Sl   855 : S-----------LYAQDIKMKEQDELENYFAAIRLKNQINAKGSTQMKK-------------------------------
Ot   853 : S-----------LYAQDIKMKEQDELENYFAAIRLKNQINAKGATQTKK-------------------------------
Ai   900 : S-----------------SGDDRTDTSTTFVFTDPPPVFHDL--------------------------------------
Cr   911 : Q--------------------KSSMLKNSLQSFQFQHGEDD---------------------------------------
```

(c) Cfap246

```
                   10        20        30        40        50        60        70        80        90
Tt     1 :------------------------------------------------------------------MSEIAKDSFYKERYYQLSLTSWGEDE
Im     1 :------------------------------------------------------------------MTEIIKESFYQDKLYQLSVTSWGEEE
Sl     - :------------------------------------------------------------------------------------------
Ot     - :------------------------------------------------------------------------------------------
Hs     1 :MATSERALLRTRAASLLRGLGRSRTGARSLQFRAEKERQPCWSFPMGQKTKGSSNIASSYLLQQLMHRYQELDSDGDEDQGEGEAGSEES
Xl     1 :-------------------------------------MALVVLSPGFREEFSPGSPRELEHDEETSERWKASSPASSTDSGDSISDSEEE
Dr     - :------------------------------------------------------------------------------------------
Sp     1 :--------------------------------MNFCSLETANMASDATESRPQTVSFSDLTERTPENLEAELVPEPPVQINTNSNDIQID
Cr     1 :------------------------------------------------------------MPSDGASVAGSSRSSMSRASRASKRGAKKS
Gp     1 :---------------------------------------------MFRTPTMEVRLRSHSVTSESDRSSVSGRSTTSRASKSSRRGPKKP

                  100       110       120       130       140       150       160       170       180
Tt    27 :KKFMENRKLKELSRNVMTEEIIKSSLGTLGKTAQGSHYAFLSFNASNKDLYSIGGIEDFKHLQYIDVSFNNLITLKPLSQLKYITYLNIS
Im    27 :QKQRLNRRLNELSKNNMTQETIKNSLKNLGKTSQGSSYAFLSFIAIQKDLYSIIGIENYKHLQYIDVSHNNLITLKPLNSLKYITHLNVS
Sl     1 :------MMSKEP----ISNDIFKAGLSCLGKTFNNARHAYLTLNIQNNNLVSIVGIERYKYLQNVDVSNNKLIHLKDLSHLKHLIKLKAS
Ot     1 :------MMSKEP----IDQEIFKQGLSCLGKTFNNSRHAYLTLNIQNNNLVSIVGIDRYKYLQNIDVSNNKLIHLKDLSHLKHIIKLNAS
Hs    91 :SESEMLNLEEEF-DGVLREEAVAKALHHLGRSGSGTEQVYLNLTLSGCNLIDVSILCGYVHLQKLDLSANKIEDLSCVSCMPYLLELNAS
Xl    54 :SDKDMSVMEAEF-DGVLREETVSEGLSMLGRSATGSEHVYLNLTLVNRSLRDIQVLCGYSHLQKLDISNNEISDLSCVSFMPYLTELNAS
Dr     1 :MIYLFKRLKTEI-AGELTEDEVFKCLSGLGQSATGLQHTYLCLSAPGRDLKNVSILCNYIYLQKLELPYNKIKDLSCVSHMPYLITLDAS
Sp    59 :SDGDEAEEQEPCPDGILNEQAIEAGLSNLGRSADGMQLTFLNLTLPGYNLQGINILENYVHLQKVELPYNRITDITVLGCMPYLVELDVS
Cr    31 :LPPPPPPEPVEEPKDAFQPSMLSAGLSQLGRTADGLKPAYLTLSLTGTDLANADVLGAFPHLQTLVLRDNRLVELRGLAALRHLTAVDVS
Gp    46 :VATPPPSEPTEDPKTPFSPNMLAGGLSQLGRTADGRAAAYLTLTLTGTDLGNAEALAAFPHLQAVRLPDNRLTDVRGLGVLRNLTAVDVS

                  190       200       210       220       230       240       250       260       270
Tt   117 :HNNLTKLLDFKEI------PYNLEEVISSHNQINEIKDLSEHKFLKKLDLSNNQIEKIEG-LSSNKDLQILKLAYNRINIIENLDHLN-I
Im   117 :HNNLNKLLDFKEI------PYNLEEVISSHNQIQVIPDLSAHKYLKKLDLSHNKITQIQG-LSKNDNLSVLKLAFNNIETIENLDDLN-I
Sl    81 :WNQLRKMFDFDP-------PANLEWVDYSGNAINKIENCDQNIYLKYLNLDSNNIQQIEG-LNHNKCLRTLSLNGNSIDTIENLDGLF-I
Ot    81 :WNQLRRMFDFEP-------PANLEWVDYSGNAINRIENCDKNVFLKYLNLDSNNISTIEG-LQMNKCLRTLSLNGNSIDTIESLDGLY-I
Hs   180 :QNNLTTFFNFKP-------PKNLKKADFSHNQISEICDLSAYHALTKLILDGNEIEEISG-LEMCNNLIHLSLANNKITTINGLNKLP-I
Xl   143 :NNRLSTFFDFAP-------PKNLRVVDLSFNQITHMTDLSAHKALTRLILNNNNIEEIIG-LDKCCSLTHLNLAHNRIHNISVFGKLP-L
Dr    90 :HNQLTDFFGFQP-------PKNLKEVNFSHNQMTAMKDLSAYSSLTKLILDHNSFSVIRG-LEKCKRLSHLSLAHNNISRIRGLDHLP-L
Sp   149 :HNEITNLLDFKP-------PFNLQEVDVSFNKITEMGDLSAHHALTKLVLDNNQLSTITG-IENCRCLHHLGLAHNNISVIEKLDHLP-L
Cr   121 :GNKLTQVLDLRLPADGASGPTNLRSADFSRNALDMLRDLSPFSRLTSLSAAHNRLERVGEGLTSLTLLKVLDLSHNRLVSVRGLERCANL
Gp   136 :GNKLTQVLDLRLGADGS-GPTNLRSADFSRNSIDLLRDLRPFSRLTSLALAHNRIERVGAGLRPLTLLKVLDLSHNRLVSCRGLEELASL

                  280       290       300       310       320       330       340       350       360
Tt   199 :VELDLMGNQIVHLTGLNQLVKLRKLNLSSNKISKLKGLVDLVQLRELRLSDNLIHRVRELYYLQNLTFLSDLDLCFNRIQNKRFYRYQVL
Im   199 :LELDLMGNQIQLIQGLKQLIYLRKLNLSCNKIVSLKGLIYLIQLRELKLSDNQIYRIKELHNLQNLVFLTDLDLCFNLIQNKRFYRYQVL
Sl   162 :EELFLQSNRIRKISGIENLTVLKTLDLSKNQISRMKGIQNTESLRFLYLSLNLIGKIGQLVYVENLPLLTELDLCFNPIQNRKYYRFQVL
Ot   162 :EDLFLQSNRIKKITGVENLPVLKTLDLSKNKITKLRGLQNTESLRFLYLSSNEIGKIGQLVFIENLPLLTELDLCFNPVQNRKYYRFQVL
Hs   261 :KILCLSNNQIEMITGLEDLKALQNLDLSHNQISSLQGLENHDLLEVINLEDNKIAELREIEYIKNLPILRVLNLLENPIQEKSEYWFFVI
Xl   224 :KELYLNSNYIKNISGLENLKSLQTLNLSCNQISSLEGLEGLNYLLCLNLEDNEICQISEITFIEELPLLHVLNLLKNPVQEQPEYWLSVL
Dr   171 :RELCLAGNMINKIENLQTLHNLQVLDLSCNRIQSLTGLQNLRFLGTVNLESNLITEIKEAAHLHDLILLREINLLKNPVQDHDDYRIAVI
Sp   230 :RFINLRCNQISVIENLDTLTRLQYLDLSGNEINSLEGLQKCALLETLDLENNQVADITDLQYIEGLKLLRHLTLLRNPIQDIEDYRLSLL
Cr   211 :RELRLGHNALQSLEPLAGLSQLQVLDVSHNRLAQLSGAAGLSSLRTLDVSCNRLGRLEELAVVRGASLLGTLDVRGNPLDKAMCLRLHVV
Gp   225 :RELRLSHNCLASLEPLAGLSQLQVLTADHNWLTHLSGLASLAALRTLDVSYNRLGRLEELAAVRKASLLGGLDVRGNPLDKALSLRLHVV

                  370       380       390       400       410       420       430       440       450
Tt   289 :YRLPGLRVLDGVNTTSEEFVKAENLYGMDLE----------------DRKRIFYEILPEEEFIDRRINIADLIEPETESDNEDGMQFVDQ
Im   289 :QRLPGLRVLDGVICSCEEFVKAENLYGNDLE----------------DRKRIFCEILPEEEFIDRRVNICELIEPETDSDNDLQIQIVDQ
Sl   252 :FHIPQLRQLDGVEALSEEKIKAENLHGLDLN----------------DRELIFQSLLPEEKFVDRRIHVLQDIPMESESEPE-NIDFIEQ
Ot   252 :FHIPQLRQLDGVEALSEEKIKAENLHGLDLN----------------DRELIFQSLLQEEKFVDRRIHILHDIPLESESEPE-NIDFIEQ
Hs   351 :FMLLRLTELDQKKIKVEEKVSAVNKYDPPPEVVAAQDHLTHVVNSVMQPQRIFDSTLPSLDAPYPMLILAGPEACGKRELAHRLCRQFST
Xl   314 :FMLQKLTVLDLKKIQIEEKVAAVNKYDPPPEVIATRDHMANVMYSMIQPQKVFDSTLPNVDTPYPMLVLTGPQSCGKRELAHRMCREFKE
Dr   261 :FLLQHLILLDKQTVTAEEKVAAVNKYDPPLEVVAARDHMTHLVYQLMQPQVIFDSTLPSLDAPYPMLVLTGPQACGKRELAHKLCQEFSD
Sp   320 :FRIPQMVELDRHRVEVEEKIAAVNLFEPPMEVIAAQDHRRALIYSSLKQSKVLMSTLPSIETPYPVLVLVGPRGSGKQALAQRLVKEFTE
Cr   301 :HLLPQVVMLDGVAVESKEKVLAANMHGADADSLRLIRRKYFPNGELDDGGGAIPPLAAGLVASAAEEEAAADGPGPGDAYVRLDAWAAS
Gp   315 :HLLPQVVLLDGVPVDSKEKVLAANMHAADAEGLRLIRRKYFPNGELDDGGGAIPPLAAGLVASAAEEEQAMDGSDS--SALLRIDAWAAS
```

```
                 460       470       480       490       500       510       520       530       540
Tt   363 :YDKEGNLIKAADSGTSKSLRNS-QVSIKSQHQKTQQSIPNFQESQSRTNNFNTNQSIYDQKSRTLQEKYDSLRNNSRSSNSINQVNQNLN
Im   363 :YNKEGDIIHQATAKTLSSMQRTGEVAIENDTKRQSQ----NFEQ---TNNFNNNQTIYERKSKSVFEQYENLKINSRSG-SLTKNQMSLK
Sl   325 :RIDPKV---GRSREFSSIGSQGRLKGMKNTNSAHSMG---SRMSSARNK-GQTPRSSVGQADKYVGEILHRVNFEDG--KSVNYYLQY--
Ot   325 :RIDPKIGMIGGSREFSTVGSQGRMKNLKGTSSAMSVG---SRVSSTKNRNAQTPR-SVGQENKHVGDMYQRVDFENGRGKGVDYYVEY--
Hs   441 :YFRYGACHTTRPPYFGEGDRVDYHFISQDVFDEMVNMGKFILTFSYGNHKYGLNRDTVEGIARDGLASCIHMEIEGVRSLKYSYFEPRYI
Xl   404 :FFRYGPCHTTRNPYFGEEKRFDYHFVTPEAFEEMTCSGQFLLTMKYSGHYYGLSRESVESVAREGLACCTHMEIEGVRSLKNCYYEPRYI
Dr   351 :YFAYGACHTTRGPYFGEEDGLDYHFVTEEEFHNMIQMGQFIQTMQYGGHWYGLSRESIERVAREGLACCVHMELEGVFSLKNSYFEPRYV
Sp   410 :YFGLGLLHTTRGQPTGGEK--DYHYVSSEQFEELLQEGEFLLTYQIMEHRYGLSLQAIESVAENGLACVITMELEGVLTMKLTHFQPRYV
Cr   391 :IKPEWVLGGGGGNVVSLADQLASVCAPPRP-TTTAAAATANGSTHNSTSG-----AAPGAGAANGHHGVRGAGVGAAPSGAAAG---GKP
Gp   403 :IKPESLLAAG--ALSSGARSVGSLGGTPGAGTGTGAALVALADQLASVCVPVGGSTGPAAVAAAAATPAAPAASGPHITGVGSGSMKAAP

                 550       560       570       580       590       600       610       620       630
Tt   452 :LKSN--------------------------------------------------------------------------------------
Im   445 :QSN---------------------------------------------------------------------------------------
Sl     - :------------------------------------------------------------------------------------------
Ot     - :------------------------------------------------------------------------------------------
Hs   531 :LVVPMNKEKYEGYLRRKGLFSRAEIEFAVSRVDLYIKINQNFPGYFDEVINADDLDVAYQKLSQLIREYLGLTEELAKSLATTADVKTS-
Xl   494 :LLVPMNKEKYDGWLRRKGLFTRPEIDVAVSRVDMYLKINQESPGFFDAVINTDDLDEAYSSLRLLVKEYLGLTDPVQSSQANAGTKSGSD
Dr   441 :LLIPSVVDNYVLFLKARGFYSNAQMETAVSRIDLYARINRERPGFFDSIIPCDDRAEAYRSLKQLVKEYLGLEEHSGGGDSSSITPDNT-
Sp   498 :LLLPLDQMKHEQRLQAVGNLSNTQVQFAVDRTNLYQHMHQNRPGFFDRSIDTTDPEMAFKLVRNLVLSYLGLEVPDSPEPSSQEQDSSS-
Cr   472 :SAANEAAWRRAQLERARVCWRWVVTHVAATAKLETTWDVAAPLFGHTAQAAAIERVLLGNGEPAPPAAPPPPPQLLG----------APL
Gp   491 :PPPPPGPNPMAQLQRARVCWRWVVTHVAPGASLDRTWDVDAPLFGPGPAAAAVERLLLGGGPPPPPRSPSTPSSMPSSAVVGSGGGASAV

                 640       650       660       670       680       690       700       710       720
Tt     - :------------------------------------------------------------------------------------------
Im     - :------------------------------------------------------------------------------------------
Sl     - :------------------------------------------------------------------------------------------
Ot     - :------------------------------------------------------------------------------------------
Hs   620 :HLKPEAHPTKYISSNMG--DFLHSTDRNYLIKFWAKLSAKKTPAERDSIHRQHEAARQALMGRIRPDHTLLFQRGPVPAPLTSGLHYYTT
Xl   584 :SVKSSIGSMQVKPSQLGTSNDLDSATRNYSSRASAKLPLQKTPVEEASVQRRQQVARQALAGKTPHTYTQLFQRGPVTAPATLGTQRHLL
Dr   530 :--STNTTGELPAASATESTDLMDSYSRNYQKKIQANMTPQRT--EMASNHRRLQQIREALSGKSPGAYAELFQRSVPAAPSSLASQSHPP
Sp   587 :IYSPSQTSSAMRSETHDSPSFSQSTGRQQGPPGIVENPPSPT-----NMRTWMKPVVDPPPRPLSQNSKKRQEASMSAIEKISYERRKSA
Cr   552 :APPAAAP---LPGGGTWSERVARLFTLLCRACELEAVPIPGYWKHAALPPGSRVHAHNHCWAGVKVNGRWRLVDPTAAALAGGHFPFFVP
Gp   581 :RPPATAPPPAPPGAGTWAERVARLFTLLCRACELDAVPIPGYWKHPRLPPGERVHSHNHCWAGVKVNGRWRLVDPAAAALDGGHFPFFVP

                 730       740       750       760       770       780       790       800       810
Tt     - :------------------------------------------------------------------------------------------
Im     - :------------------------------------------------------------------------------------------
Sl     - :------------------------------------------------------------------------------------------
Ot     - :------------------------------------------------------------------------------------------
Hs   708 :LEELWKSFDLCEDYFKPP--FGPYP-EKSGKDSLVSMKCSLFR----FCP-WSKELP------FQPPEGSIS----SHLGSG----ASDS
Xl   674 :DPASCSSMLPSGSNVTAERSFPPASTDTSSRDSRPSSGLSLLSSAGAFSARRSSSQPTPVITIHSPPEDTIEPLDLSGIKQGSDSISDHH
Dr   616 :AGPSSSPVRPAG--LSAP----NLSEDSSSEESRASSGLSMRSSAGVFSAESPARGS------DAGSGPNIEPLDISMLGQD-----RDS
Sp   672 :AKEAVSGIVPRPLDQLIN---EPPSTAPGKQFAGLGDDPTRPKTVPNSAVRYLAGSP------DSSVATSRSDSRMSGLSDAR----GMS
Cr   639 :PDAFIYSYWPLEAAWQLLQEPLSQEVWWQLPYASVAFFAEGCRLGDASLAAVNTLLPIRQGGVLPAFALAVAAPRRRGCHLAQRLYDR-A
Gp   671 :PDAWIYSYWPLEAAWQLLQDPLGHEVWWALPYASVAFFAGGCCLGNPDLAAVNELPAVREGGVLPAFQFNLAAPRTPGCHLAQRLYRVGS

                 820       830       840       850       860       870       880       890       900
Tt     - :------------------------------------------------------------------------------------------
Im     - :------------------------------------------------------------------------------------------
Sl     - :------------------------------------------------------------------------------------------
Ot     - :------------------------------------------------------------------------------------------
Hs   776 :ETEETRKALPIQSFSH-EKESHQHRQHSVPVISRPGSNVKPTLPPIPQGRR---------------------------------------
Xl   764 :ENGKTPDTPRLPSSAHSDTQSATSSTRPSAHTSRPGSNTKPILPPIPSGRRKSSTST---------------------------------
Dr   689 :LRGLTPDAP--------RPASDRVSPTHPSSPGRPGANVKPVLPPIPSGRKTNDSGSKQ-------------------------------
Sp   749 :ASPSGSQTS-------FQMVGGSDGHASRPGSVGSLPEDRPVLPPILTGQN---------------------------------------
Cr   728 :RRVVAEWPPREPGAPAYAFQQVVAGPHGQVEVSFGADSHAAQLHQMYFSLPAPGEYEMEVSHVRELPGGGLTLAVEGLPAPGLALRVVEE
Gp   761 :RRLVAEWPPKEPGVPAYAFQQDVDG-----------------------------------------------------------------

                 910       920       930       940       950       960       970       980       990
Tt     - :------------------------------------------------------------------------------------------
Im     - :------------------------------------------------------------------------------------------
Sl     - :------------------------------------------------------------------------------------------
Ot     - :------------------------------------------------------------------------------------------
Hs     - :------------------------------------------------------------------------------------------
Xl     - :------------------------------------------------------------------------------------------
Dr     - :------------------------------------------------------------------------------------------
Sp     - :------------------------------------------------------------------------------------------
Cr   818 :QQLLRVKVTLPGIQPHDEAEDGILHSPAVLRTPLPFGSPHWYEAGCQLISPPPHRPLEADRSEPFKLVVPGASRVGLMAEGLQQPIELAA
Gp     - :------------------------------------------------------------------------------------------

                1000      1010      1020      1030      1040      1050      1060      1070      1080
Tt     - :------------------------------------------------------------------------------------------
Im     - :------------------------------------------------------------------------------------------
Sl     - :------------------------------------------------------------------------------------------
Ot     - :------------------------------------------------------------------------------------------
Hs     - :------------------------------------------------------------------------------------------
Xl     - :------------------------------------------------------------------------------------------
Dr     - :------------------------------------------------------------------------------------------
Sp     - :------------------------------------------------------------------------------------------
Cr   908 :ADDDSFAFSTSLQVPRCRAATLMAYMHNDHLSSCAWVPLVQLAVLPQSQHMVYTPIAVEVPEVGDDDPAAHFAREIFKAMDKNLDGGVCR
Gp   786 :--------------------------------------------------------------------------------------LVTR
```

```
             1090      1100      1110      1120      1130      1140      1150      1160      1170
Tt      - :------------------------------------------------------------------------------------------
Im      - :------------------------------------------------------------------------------------------
Sl      - :------------------------------------------------------------------------------------------
Ot      - :------------------------------------------------------------------------------------------
Hs      - :------------------------------------------------------------------------------------------
Xl      - :------------------------------------------------------------------------------------------
Dr      - :------------------------------------------------------------------------------------------
Sp      - :------------------------------------------------------------------------------------------
Cr    998 :REILAAFRRNRQHADILKCPARIRQDDGTFERFVDVFMQIDTSKLGTFTFNELAFYMGVLPTGYYQYSDDEDEDEDFDSGDEQDLDPEER
Gp    790 :REILAAFRRNRQHADVLKCPARIRQDDGTFDKFVEVFMQIDSSKLGTFSFNELASYMGVLPSGMYESSEDEDGEYGSSDEGGEEDGDEGE

             1180      1190      1200      1210      1220
Tt      - :---------------------------------------------------
Im      - :---------------------------------------------------
Sl      - :---------------------------------------------------
Ot      - :---------------------------------------------------
Hs      - :---------------------------------------------------
Xl      - :---------------------------------------------------
Dr      - :---------------------------------------------------
Sp      - :---------------------------------------------------
Cr   1088 :AGRQAELDAELDAPDDEGEGGGGGPGGGEASGYTGDSAGGGGGEGEGPSEA
Gp    880 :EEGEGGGEE------------------------------------------
```

(d) Cfap174

```
                  10         20         30         40         50         60         70         80         90        100
Tt    1 : MSFQ-TTDSKKEEFRKYLEKAGVVDQLTRVLVGLYEEPNKPNNAVDYIKKYLG--SPTDIDVERLQVEYEKLKDENLRLKKTVEELKKE---LEQLKPEDN--------
Pt    1 : MSFQ-TTDSKKEEFRKYLEKAGVIDQLTRVLVGLYEEPEKPNNAIDYVKKYLG--SPVDIDVDKLKLEYEKLKDENIRLKREVAELKKE---LQAAQQEQN--------
Sl    1 : MSVQ-TTEQKKEEFRKYLEKAGVVDQLTKVLVGLYEEPEKPSNAIEFIKKCLG--APSDTDVEQLKADNEELRRQQHEMEKKIEALQRE---LEHERAKQ---------
Sp    1 : MTTYRPGDSKREEFRKYLEKAGVLDALTKILVALYEEPEKPNDALDFLKAHMGQGGPDTADVESLRLQVSELMQKVERLQDENRELKAK---VQMENPEVEPNPEN---
Ci    1 : MTSYRAADSKREEFRKYLEKAGVIDALTKVLVNLYEEPEKPNNALEFLNHHLSGDGPETPDVEALKVDVIELKAKVSQLTDENIELKTR---LEKYEPSVAQDDTAE--
Hs    1 : MAHYKAADSKREQFRRYLEKSGVLDTLTKVLVALYEEPEKPNSALDFLKHHLGAATPENPEIELLRLELAEMKEKYEAIVEENKKLKAK---LAQYEPPQEEKRAE---
Xt    1 : MANYKAADSKREQFRRYLEKAGVLDTLTKVLVALYEEPEKPNNALDFLKQHMGAAGPETADVEALRLEVAELKQKYEAVLEENKELKAK---LAQHDPAQHLDEKRSET
Dr    1 : MAHYRASESKREQFRRYLEKAGVLDSLTNVLVALYEETEKPNNALDFIKHHLGVSGVEAEDAESLREELNTLQQKHQQLMEENQELKSR---LLQYEPPQ--DEAAAVE
Cr    1 : -----MSESQKETFRKYLEQAGAIDVLVKVLVQLYEEPSKPKTALDYIKQCLG--SPTPAEYEAVVAERDGLQKQLEEANQLIAELQSR---VQSLEAAAETA------
Gp    1 : -----MSESQKETFRKYLEQAGAIDVLVKVLVQLYEEPSKPKQALDYIKQCLG--SPTPAEFEAVLAERDGLQKQLEEANQQIAELQARNVDVASREPTAKFH------
Vc    1 : -----MSESQKETFRKYLEQAGAIDVLVKVLVQLYEEPSKPKQALDYIKQCLG--SPTPAEYEALIAERDGLKSQLEEAHQQIAELQAR---VQQLEASAEAS------
```

(e) Adgb

```
               10        20        30        40        50        60        70        80        90
Xl      - :------------------------------------------------------------------------------------------
Hs      - :------------------------------------------------------------------------------------------
Dr      - :------------------------------------------------------------------------------------------
Ci      - :------------------------------------------------------------------------------------------
Tt      1 :MPPKQQKQKAPVGYKAAALAKDILPTSVKNPPREGKSVKIPDAVAIQQNQAIANQLNAPLFEEYPKIEEWPGDDAAKNFDFNIEGTTNFV
Pt      - :------------------------------------------------------------------------------------------
Sl      1 :--MPPKGKDPKKGAPVGNYKAGKLMKDILPPNTKPPREGGQIKCEGDPDVLRTYAYEPYQNFPEWVSNEDAKNHDFTAGFQKHDDGSYQK

              100       110       120       130       140       150       160       170       180
Xl      1 :MSSKLSKKRESGHRLSSALG-QTPRDIASLFASSLTSSIEVKKGKVTIWPEWNDSDIN--------SEKWDSGKGTKEKEKTGKIPALHF
Hs      1 :MASKQTKKKE-VHRINSAHGSDKSKDFYPFGSNVQSGSTEQKKGKFPLWPEWSEADIN--------SEKWDAGKGAKEKDKTGKSPVFHF
Dr      1 :-MSKTPKKKASASSLVASSPGHSPKKEASSLVGASESPVESKKSLISLWPEWNDTDVN--------AEKWDAPKTPKDSK---------L
Ci      1 :-----------------MESQARAKRAASLVGSVASVDSKGKPRNFPIWPEFLESDIN--------AEKWEGVTKGKEKGKS---PSLQF
Tt     91 :DQNFSTNKLTPSFVNDNSNLIFWRRPTEFIQIEQEELQTIQNKYNIKRRTSYYQTVVS-------EEHSFKKKEEFQHSPLKG-------
Pt      1 :------------------MAIVNKYISDVRESEHEPPYMDMRENRYNMNDDYYS-------------TRLETVEKIHY------------
Sl     89 :FEDHTPIYLPPSYKEYEKGEIMWLRPEEYLREIAYDSEMAKRRQEKKQQIKRKKTIRKQSILSIGNVNNLEENLNQLNSKLGGDDKKEGI

              190       200       210       220       230       240       250       260       270
Xl     82 :FDDPEGKIELPANLKIHSWKRPHEFIASMPPVVVKDETSFDLFSANKHLTG----------SELMRWIISEVNAVWKIYNTNLINN--KA
Hs     82 :FEDPEGKIELPPSLKIYSWKRPQDILFSQTPVVVKNEITFDLFSANEHLLC----------SELMRWIISEIYAVWKIFNGGILSNYFKG
Dr     73 :FDDPEGKVELPSSLRIHTWKRPSEFILKKIPVVVENADTFDLTSANEHLFS----------SELMRWIISECHILWKVCN----TREDKV
Ci     63 :FEDPEGRLDLPTSLAVENWKRPQDFMLGKIPVVVENESEFNLLQYNEHICH----------SQLMRSIITQVTALWEMNNNNVPDSHHSS
Tt    167 :LHDEPTKLQVKVFEYYEREETQQEFEQRKKEQMEKEEKEKK-TKKPAKKVEQVED----KPGLVKDIKLNSIDLSYQYPSDSKWIASQLQ
Pt     48 :-MEKEVEHHLTICESFERMETPEEVEMRRKEFLEKQAAQQKNKKGNKKQAEEVFDE---NPKMTSDVRLSDLICSDELPPNSRWIASQLQ
Sl    179 :VNRDELQFDMICITYDQRLETEEEVKKRKDEAEKLAAADKNAKKKPPAKGAPAQNDPLDEPQMIKVPVENTMDMGFLMPIYSKWVTSQLQ

              280       290       300       310       320       330       340       350       360
Xl    160 :STQDPTSLVWKPWEHIYALCKAAKGHMPLYNSYGKYIVKLFWMGSWRKIVVDDTFPFSEDNKLLLPATTCETELWPLLLSKAIIKLASIN
Hs    162 :TSGEPPLLPWKPWEHIYSLCKAVKGHMPLFNSYGKYVVKLYWMGCWRKITIDDFLPFDEDNNLLLPATTYEFELWPMLLSKAIIKLANID
Dr    149 :VSTETVPTLWKPWEHIYSLCKAGKDHMPLYNTYGKYVIKLYWLGSWRKITVDDMLPFDKDNNLLLPATTNQSELWPMLLAKGILKVASTD
Ci    143 :VDGDP---GWSPWEHIYSLCKATKGHIPLYNQYGKYVVRLFWMGHWRKIVVDDQMPFDANDQLLLPITTSNHELWPLLLTKALVKIAAID
Tt    252 :LIKDRNIRDCYTNKPLHSKIYPQQDNIPVYNKTGRYWVKLYYLGKERKIEIDDKMPVNFKGQCKFPRSVKKEEIWTCIITKAIFKLMQIS
Pt    134 :QIKDRDIKDCFTQKSLSSKIYPQKDGAPIYNPNGKYIVKLYFMGKERKIQVSDHMPTTYDGKALLPQSVDKNQLWPMIISKAVLQLWDYQ
Sl    269 :FIKDRSIRDCESREPIWQRIYPQENGIPVISPTGKYQIKIRFMGKERLVEIDDRMPCDSKKKLMFPRSINNFEIWPQLLLKALLKVYSYK
```

```
                370       380       390       400       410       420       430       440       450
Xl  250 :TNG--FAK-RELGEFTVLHSLTGWLLEVIPLENNYLNEVWDLLKGIVPEYTLPDEENPDAKSPTTDDKSKE----------IKP-PEVKI
Hs  252 :IHV--ADR-RELGEFTVIHALTGWLPEVISLHPGYMDKVWELLKEILPEFKLSDEASSESKIAVLDSKLKEPGKEGKEGKEIKDGKEVKD
Dr  239 :TPV--SGSGREFGEFTIIHCLTGWIPEMIPLKSWYTGKIWDFLRDNVAQFQLE-EESSEEKITITDSNVNN-------------------
Ci  230 :YVC--NNDASEIGDFYVIHALTGWIPEVIPIKQSPSENIWEFLKQSLPQWTLPETNPAEDENKTSSASSKR-------------------
Tt  342 :QDI--TCINNLCGSGFVMYTLTGMLSQSIPMQINSKSTSEWQNKLNFIREMLNDKHYLDQDILVTCYSEPG-------------------
Pt  224 :SK------GSLVGDGFVMYSLMGLLTETID--------LKTINSWGIIDNMMNNNHYIQKDVFVSTYSYKN-------------------
Sl  359 :WFTPQAYFDQEVGDGSIVYSLTGLIPEPVAIK------DFEKEGLELFRKVLSDDHYFNKKTYVTCYCDNE-------------------

                460       470       480       490       500       510       520       530       540
Xl  326 :DGPVIP----VIIKPPEKSNKE------QRDAGKKK---GEKEKIRSASHSARPASELSNPLLQSTQDCSMVQMTPQMVVYASYSPLQLS
Hs  339 :VKEFKPESSLTTLKAPEKSDKVPKEKADARDIGKKRSKDGEKEKFKFSLHGSRPSSEVQYS-VQSLSDCSSAIQTSHMVVYATFTPLYLF
Dr  307 :------------------------------------------------VQSKSPKSRATGS-------------VPQIVVCASYQPLHLM
Ci  299 :----------ENVPAPTNSASHKEKGSGKDSKSREGKKRDDKEKSKSGSHSGRPIS-ANSAAKSAMEDDPSVAKTPEMVVFASFNSSCDS
Tt  411 :---------------------------------------------------FQPKIEQGQN--------------NSRLSVVQQKKSKDS
Pt  281 :---------------------------------------------------QSIQNTNN-----------------------RSKKEYQL
Sl  424 :---------------------------------------------------FRPKLPSQTT---------------TLKKIDFGPNQPAH

                550       560       570       580       590       600       610       620       630
Xl  403 :EHKTSVLGQMADSSEKLRQYGLSHIYNHPVLVTRTRSCPLVGSPKPPPVPRWKLIRPKKEITVTDEAKEPVEKQP--EQFVEISTPFLNF
Hs  428 :ENKIFSLEKMADSAEKLREYGLSHICSHPVLVTRSRSCPLVAPPKPPPLPPWKLIRQKKETVITDEAQELIVKKP--ERFLEISSPFLNY
Dr  336 :EKRTSMLTRMADSSESLRQYGLSQLFSHPVLLTCTRDCPLVAPPKTPPLPQWKLIRPRKQTNITDEPKEPPVQKP--EQFIEVSSPFINF
Ci  378 :PTSLTELRQMADISQKLRKVGLSYLHSHSVYVKQSRSCPLVATPPEVPIPKWKLIRPKKTFVTPHDQPKPKEKAPDPEMWLNISTALLGH
Tt  436 :SELAEQIKEESPIDRRKSQDEEQGHQKHTKMFIEPIKIPQRGRESADSSNDDTHQRSPYKRKQQRNLTTGGIVRYSTQVFDQMHKAMHDP
Pt  297 :ELLRNPQKEFDNYEVARNKNGSP--------------------SQHQQMQQEEQDEQQEEMQQEENEQK-------VQRFRKTQRGFS--
Sl  448 :SNSLADKGSSIEVESEPSSGVSVTSSGRMLNRLRDAATMAISVTTGRKLNITNKSSTSSNVITGFGYALMDIFENQFVDMDSIVKKNDEI

                640       650       660       670       680       690       700       710       720
Xl  491 :RLNSINVPTEVSKVQTSPSRSSLAAFSLASLNETEENVESNNIIKSLQLCAGDETEASSIIKDATGLNKGTDG----VTENTPLDMCTSM
Hs  516 :RMTPFTIPTEMHFVRSLIKKGIPPGSDLPSVSETDETATHSQTDLSQITKATSQGNTASQVILGKGTDEQTDFGLGDAHQSDGLNLEREI
Dr  424 :KLMTMSAHDMEHGGLKKRMYNSNLTSFSEMEDTEDEHIQNDNIQNSTNTLQTNENTQVPAEDKKKDNSIANDTEGVESTAAPDKETDRKD
Ci  468 :TIRPLPVDTTTSRPHSR-----------VSSPLTEINERGKNIRFSQNSMHNGESESASPREDGTEN---------KSNVNSPENVEGNA
Tt  526 :HISGIEADEEEVANNSMSIQNQSMSPTKRQNPMLQEIKKASSIIQQTQKNIPTNVISGFAYPLIECFYNDT-FNMVYVQKRTDKEIKLRQ
Pt  358 :---------------------------------FESLSRSYSTPKPPKVPGDTNMNTCFSYSIVEQFQNENGFNMVYAQKRSDRELRLRQ
Sl  538 :EEVKSPFISPAKKRREKGLSKEEWKKKRREEKKREQEEKERREREPPKQFSLVKIKTSVGHYPVMNYMSTFTNDEIHEGKKCLLNRMRRP

                730       740       750       760       770       780       790       800       810
Xl  577 :SNQPTHGIEKNP-----ENKVMEKKLPPTQTWMDFSDFCKCFQTLYVFHKPNTYPYTCRKSDFK--------------------------
Hs  606 :VSQTTATQEKSQ-----EELPTTNNSVSKEIWLDFEDFCVCFQNIYIFHKPSSYCLNFQKSEFK--------------------------
Dr  514 :KEKVQTGKESSAGLLQSQDALASNKPLLLKTWVDLHNFTKCFQTLLIFHKPNMYPHQFKTSHFTNSIS------------SRLSAAALSY
Ci  538 :EDQMSEGNESTD--------QPAKPVAEQSTWIHFYDFCQCFKDLTIFHKTSTYTYNHTVTELKPAALNAGAGANTGGSSSKKGSAQLTQ
Tt  615 :EYVELSNQSINKMS----KEEKIEHRKKKKDIRDKIGDEEKKRIELITRPPIQYKYFRLKSGAG--------------------------
Pt  415 :EYFDLCKTPMNKMT----KEEKLDLRRRKKEIKEKLQDDDKKRISLISQQPVKHRYLRLKSDVS--------------------------
Sl  628 :PEIERADSPITAKT-QHQHKHHGGFQLNVQALDQNANANETKQGDDHRQEEEKRQMVEPKTRAAG-------------------------

                820       830       840       850       860       870       880       890       900
Xl  636 :------------------------TTDERGFYYLHIDNLKPTEVLVSFSALAHWG-----------------DS-PFTEE-PGLQKSLLT
Hs  665 :------------------------FSEERVSYYLFVDSLKPIELLVCFSALVRWG-----------------EYGALTKDSPPIEPGLLT
Dr  592 :TVASTHSVAALPKQSDLTGNIHTQSADGKGTYFLFADNLLSTEIVIGFSALVHWG-----------------ESVEERKE-SSTRPGSLT
Ci  620 :ASSNTNVLKSGLPSAITPKSYEGYPIEDRVPSFLFVDSLKQIQLIINYSVLPRWAGLEEKRMSDRESIADASDAPSVKQDNQSCNSGLLV
Tt  675 :----------------------------KVPQISILTPFSNDEIQIAKTCIVNKLN--------------KPPNYQDGAEIRDDGKSIVS
Pt  475 :----------------------------GKDPVIVFSPLAADEIYIAKKCIANKLN--------------KPPNYDI-PEIKGDDKSVVS
Sl  692 :-----------------------------GVWLLASDFPFSFQHMIVYHNINKYSN----------------------IQTFSDKWQDPT

                910       920       930       940       950       960       970       980       990
Xl  683 :AEQFSWKNVCTGPVVLKIHTYVTKSTMISLPPGRHVLRFTASSPFGHHIHLCSTEPFVFGDEETVMPYLEKESCRFMEQVKHILKAVWKV
Hs  714 :AETFSWKSLKPGSLVLKIHTYATKATVVRLPVGRHMLLFNAYSPVGHSIHICSMVSFVIGDEHVVLPNFEPESCRFTEQSLLIMKAIGNV
Dr  664 :AKPFSWKSIISKLPVVQIQTTACKAVLLSLPPGRHVLQVHTNASLAVHLHLCSKFPFVFGDEETVMPYLDKESLRFCEQAVTIVRALGCV
Ci  710 :AEPYSWKTLIKGQPVIRIRTTGTKAACVSLPPGRHVLQLTFNSPLGFHLTVCSNTKFTYGDEETVMVPLQNESCRFVAVADNIMTALGKC
Tt  723 :SVMKNQEETLQMKPLDDFSSVNNFVEPLNKGQGGVWILDNDLIGCFQNFQIFYNPAKFPHHKTINIAQAVDSEITTGEDNEVLLVERN--
Pt  522 :ANMKNTDDSFTVKPLEDFSSVSNAQEPFNRGEGGFWVHEKEFLSLFDYIQIAYDPKRYN-MQVLQIQSNPDNDIAGYDNIEMVIVQRD--
Sl  731 :QPYISNEKDIVIKLELDEEALKSVHQHQSQAQSLDISKQSHNNSAFIEPTIGGEESRIDGLSKLEYQLLPGEIIPQPQNIDQILLAFAP-

               1000      1010      1020      1030      1040      1050      1060      1070      1080
Xl  773 :FNTFSNENELSQALKELELAVYPQKRGSLQLAKEHFQIFKTALWQVFVEAMGSKVTQDLIFAFRALTLDFDLV--------LTKNTDTKY
Hs  804 :IANFKDKGKLSAALKDLQTAHYPVPFHDKELTAQHFRVFHLSLWRLMKKVQITKPPPNFKFAFRAMVLDLELLNSSLEEVSLVEWLDVKY
Dr  754 :ISCFSDPEELPLTTKELEKAHG--------MGIKQQRVFSEAVYHMFFSALGRKLTSEELFAVQTLTGDPSPHGSN----EKLLEARSAD
Ci  800 :IESFGNTENWLKARQELHNAHMPYTAKNTKQLKEHYKAWKSALYQTFSSVSENKIP-DLDFVFRCFTLDCEFDS-------LQGDAKASP
Tt  811 :--------DNKENPD----------------------EDVQMLFGFTAKESKTA--KIIPEPYCILQK---------------FDFGSFS
Pt  609 :-------PDSDDNAQ------------------------VAFLVGFQPKYSQNKDFNEQIRPYSMLQR---------------FDLETYE
Sl  820 :-------HPSKEPTQVLP----------------------RYLMRLTQLDAGGSAGQQSLASARGKQNQE-----------LAQEDKPID

               1090      1100      1110      1120      1130      1140      1150      1160      1170
Xl  855 :EIPPSWQKRDATSQEEAAAMKLQACWRGIYIRKFGQSRKAGT-KEKSCVQETLEKVRNALEPNAEQHGISLLRYMFKHSSSSCE-YSCSE
Hs  894 :CMPTSD--KEYSAEEVAAAIKIQAMWRGTYVRLLMKARIPDT-KENISVADTLQKVWAVLEMNLEQYAVSLLRLMFKSKCKSLESYPCYQ
Dr  832 :ETPEGWTGRQATDKEMQAAIVLQAGWKGYLVREILTAARPGT-KENVIVAKTLQEMWASVESDVEKHAVSLLRHMVTNGEGIAELYPCRE
Ci  882 :EVPEVWKSKSASPLEEDAVTTLQKHWKSLYVRRIRRGHKDDTSEEHTSVKEGLTIIWTGMLADIQAQGLSLLRSMMDENSEIMKNYSFND
Tt  854 :AIHEFQPLKGNQSSQMITLDNSNQVFKILVYTPVSFSLNISC-HNSFRTMSVLNYLTEYEGYSVKNFNIEYLAMEEKKYHVFFRFRIPQQ
Pt  653 :SILDYKLLNQCVSSQMLLLDNKNHIFKMNIYSPLNFTMWLTS-NNKISTQSVFDYLIDYENYQKQSFQIDYPACEANKYYVYFRFKVFLN
Sl  870 :MIFKSYLEGKLLTVNGLNQESNCIWLKPNIIAPQGFTIWAAGLVRKISILPKSDYLIHQKQFFLKSYQSEFLPIEKGKYQLFGKFDFNVN
```

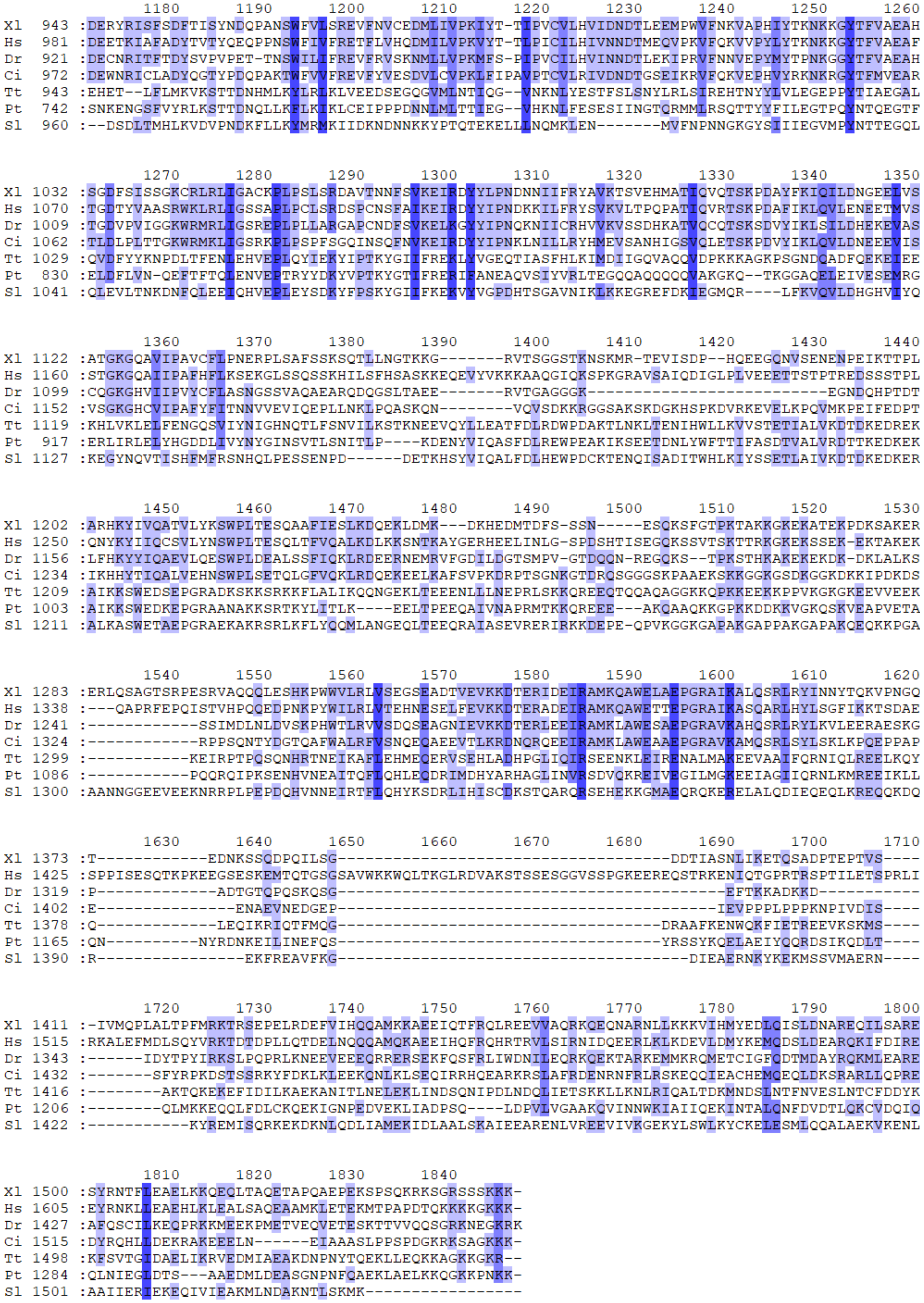

Supplementary Figure S2. Multiple alignments of C1b/C1f-related proteins. The amino acid sequences of the analyzed proteins were obtained from the NCBI protein database using pBlast search and *Tetrahymena* protein as a bait and next aligned using ClustalX2 program [67]. If necessary, alignment was edited using SeaView program [68]. The identical and similar amino acid residues were marked using GeneDoc program [69].

(a) Spef2 homologs' sequences: *Cephus cinctus* (Cc, XP_015596915.1), *Chlamydomonas reinhardtii* (CPC1, XP_001702926.1), *Ciona intestinalis* (Ci, XP_002120578.1), *Danio rerio* (Dr, XP_009299457.1), *Gonium pectoral* (Gp, KXZ56759.1), *Homo sapiens* (Hs, XP_005248434.1), *Ichthyophthirius multifiliis* (Im, XP_004034655.1), *Orussus abietinus* (Oa, XP_023288297.1), *Oxytricha trifallax* (Ot, EJY88251.1), *Paramecium tetraurelia* (Pt, XP_001425353.1), *Stylonychia lemnae* (Sl, CDW76374.1), *Tetrahymena thermophila* (Tt2A, XP_001030106.2; TTHERM_01142770), *Tetrahymena thermophila* (Tt2B, TTHERM_00633390), *Volvox carteri f. nagariensis* (Vc, XP_002946910.1), *Wasmannia auropunctata* (Wa, XP_011695725.1), *Xenopus laevis* (Xl, OCU02606.1).

(b) Cfap69 homologs' sequences: *Aphanomyces invadans* (Ai, XP_008863697.1), *Branchiostoma floridae* **(**Bf, XP_002609706.1), *Chlamydomonas reinhardtii* (Cr, XP_001703508.1), *Ciona intestinalis* (Ci, XP_002126046.2), *Homo sapiens* (Hs, NP_001153610.1), Oxytricha trifallax (Ot, EJY73745.1), *Pseudocohnilembus persalinus* (Pp, KRX04785.1), *Stylonychia lemnae* (Sl, CDW80109.1), *Stylophora pistillata* (Spi, XP_022782599.1), *Strongylocentrotus purpuratus* (Sp, XP_011682215.1), *Tetrahymena thermophila* (Tt, XP_001032130.2; TTHERM_00691650), *Xenopus tropicalis* (Xt, XP_002939054.2).

(c) Cfap246 homologs' sequences: *Chlamydomonas reinhardtii* (Cr, PNW73098.1 14g618750v5), *Danio rerio* (Dr, XP_021330922.1), *Gonium pectorale* (Gp, KXZ48378.1), *Homo sapiens* (Hs, AAI04898.1), *Ichthyophthirius multifiliis* (Im, XP_004039524.1), *Oxytricha trifallax* (Ot, EJY76869.1), *Strongylocentrotus purpuratus* (Sp, XP_786893.2), *Stylonychia lemnae* (Sl, CDW89216.1),
Tetrahymena thermophila (Tt, XP_001016516.2, TTHERM_00188400), *Xenopus laevis* (Xl, XP_018108269.1).

(d) Cfap174 homologs' sequences: *Chlamydomonas reinhardtii* (Cr, ACR55627.1), *Ciona intestinalis* (Ci, XP_002127905.1), *Danio rerio* (Dr, NP_001035140.1), *Gonium pectorale* (Gp, KXZ44550.1), *Homo sapiens* (Hs, EAX07292.1), *Paramecium tetraurelia* (Pt, XP_001440760.1), *Strongylocentrotus purpuratus* (Sp, XP_030851030.1), *Stylonychia lemnae* (Sl, CDW75994.1), *Tetrahymena thermophila* (Tt, TTHERM_00077420), *Volvox carteri f. nagariensis* (Vc, XP_002950671.1 VOLCADRAFT_85404) *Xenopus tropicalis* (Xt, NP_001017035.1).

(e) Adgb homologs' sequences: *Ciona intestinalis* (Ci, XP_002124575.1), *Danio rerio* (Dr, XP_009293055.1), *Homo sapiens* (Hs, XP_016866804.1), *Paramecium tetraurelia* (Pt, XP_001446952.1), *Stylonychia lemnae* (Sl, CDW78458.1), *Tetrahymena thermophila* (Tt, TTHERM_00290850), *Xenopus laevis* (Xl, XP_018118544.1).

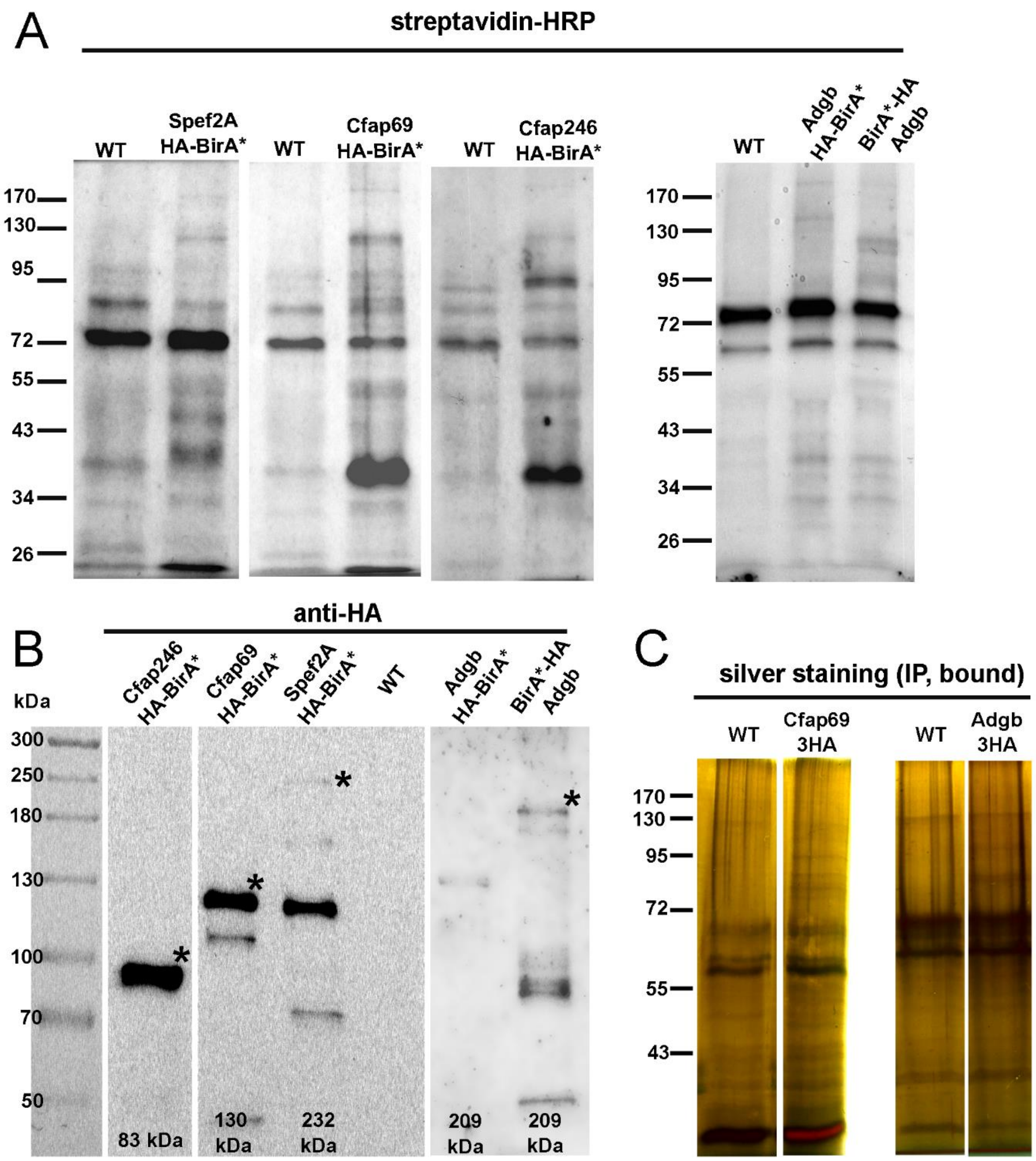

Supplementary Figure S3. Identification of the ciliary proteins positioned in close proximity of *Tetrahymena* Spef2A and potential interactors of Spef2A. **a** Western blot-based detection of the biotinylated proteins in cilia isolated from either wild-type cells (WT) or cells expressing analyzed proteins fused with HA-BirA*: Spef2A, Cfap69, Cfap246, and Adgb. **b** Western blot analyses of the ciliary proteins using anti-HA antibodies confirming a presence of HA-BirA*-tagged proteins expressed under the control of the respective native promoters. Bands corresponding to the full-length proteins (based on the predicted molecular mass) are marked

by an asterisk. **c** Silver-stained gel showing proteins immunoprecipitated from a ciliary fraction of cells expressing either Cfap69-3HA or Adgb-3HA at a native level using beads coated with anti-HA antibodies. ciliary proteins purified from wild-type cells (WT) were used as a control.

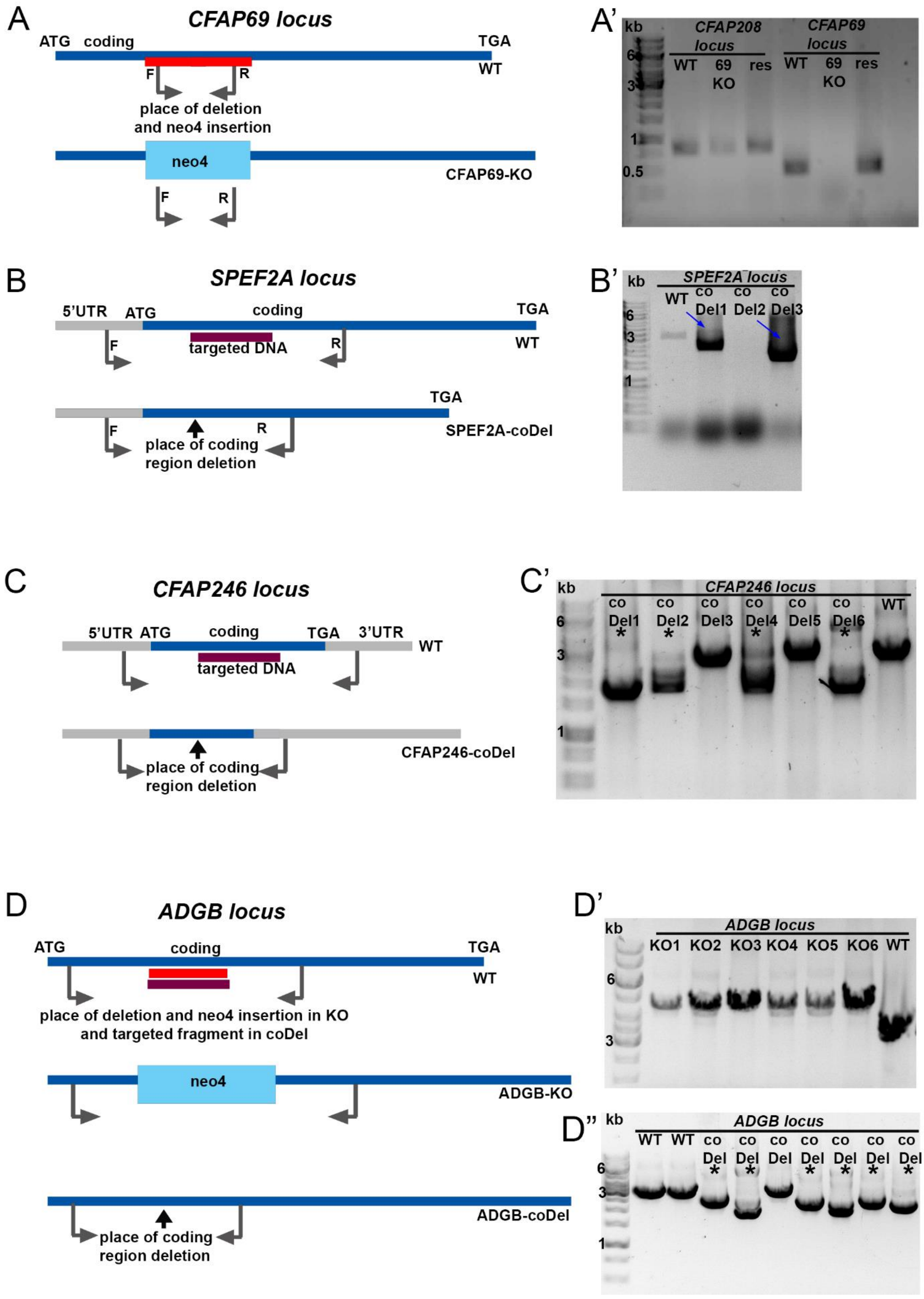

Supplementary Figure S4. PCR-based analysis of the targeted loci in engineered *Tetrahymena* deletion mutants. **a, b, c** and **d** Schematic representations of the targeted loci in wild-type cells (WT) and after transgene incorporation: *CFAP69* (**a**), *SPEF2A* (**b**), *CFAP246* (**c**), *ADGB* (**d**).

Arrows indicate the position of the nucleotide sequence recognized by primers used in PCR. Red (**a**, **d** - germ-line knockout approach) or brown (**b**, **c, d** - co-Deletion approach) rectangles illustrate the size of the targeted fragment of the gene and corresponding position in the locus of wild-type cells. After homologous recombination, a targeted fragment was replaced by neo4 resistance cassette (**a**, **d** blue rectangles) or deleted (**b**, **c, d** pointed by an arrow). **a'**, **b'**, **c'** and **d'** PCR analyses of the targeted loci using primers indicated on corresponding schemes showing deletion of the fragment of the targeted genes. Please note that: **a'** both primers recognized a nucleotide sequence removed from the *CFAP69* locus in engineered mutants and thus a fragment of the DNA is not amplified if genomic DNA purified from *CFAP69-KO* cells is used as a template, **b', c', d"** fragments amplified by PCR differ in their size (smaller fragments are obtained when the genomic DNA from mutant cells was used as a template; note also that co-Deletion approach may results in some differences in the size of the fragment deleted from the targeted locus. **d'** the amplified DNA fragment is larger in obtained mutant cells due to replacement of the approximately 0.75 kb fragment of the open reading frame by 2 kb neo4 resistance cassette. Arrows (**b'**) or asterisks (**c'**, **d"**) indicate DNA fragments amplified by PCR using genomic DNA purified from co-Del cells. Note that these fragments are smaller than one amplified using genomic DNA from the wild-type cells. The extent of the deletion of the targeted gene in coDel mutants was analyzed by DNA sequencing.

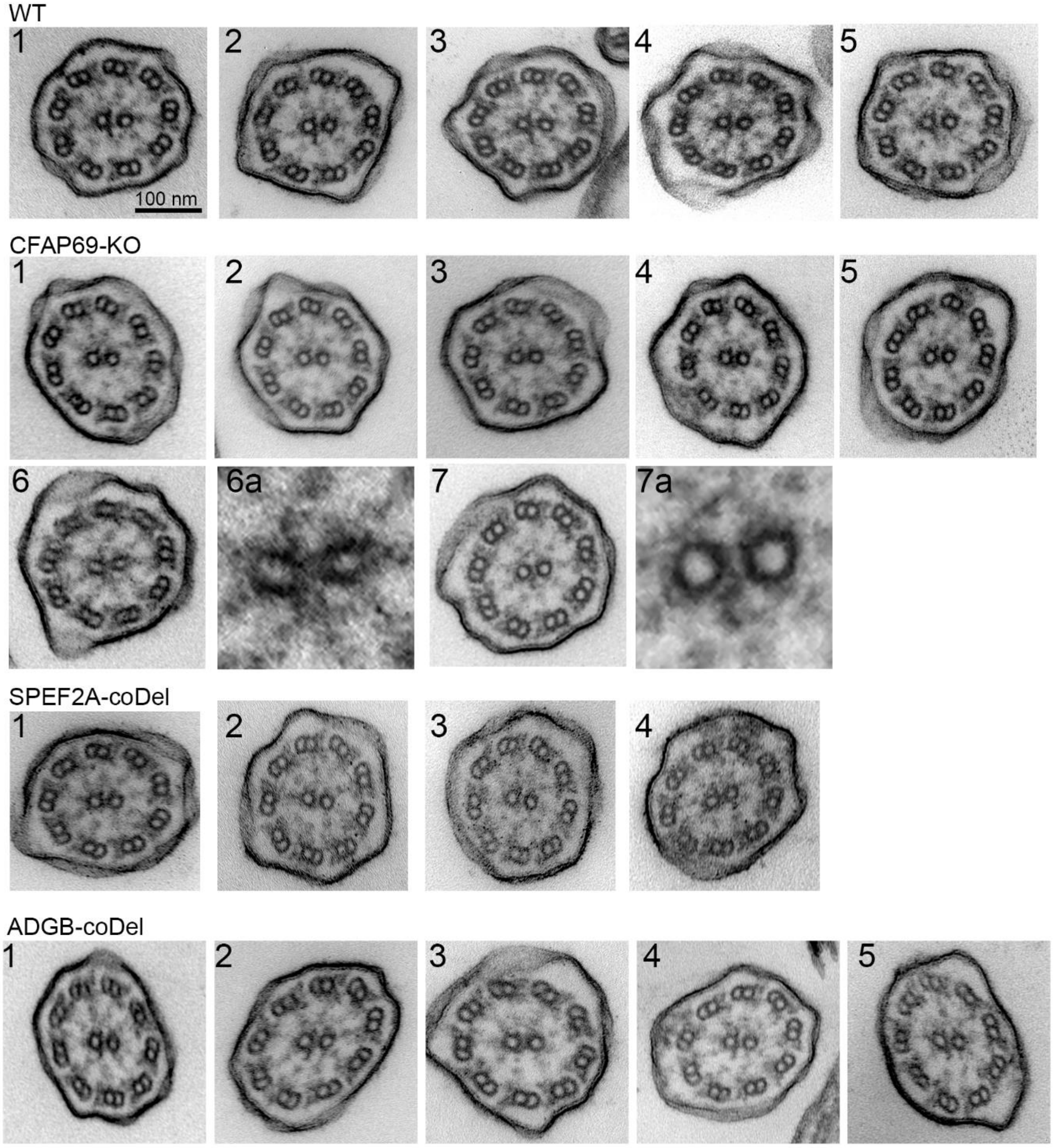

Supplementary Figure S5. Deletion of *SPEF2A* or *CFAP69* causes defects in C1b central apparatus projection. TEM analyses of wild-type and mutant cilia ultrastructure. First image in each row is a copy of an image shown in Fig. 2.

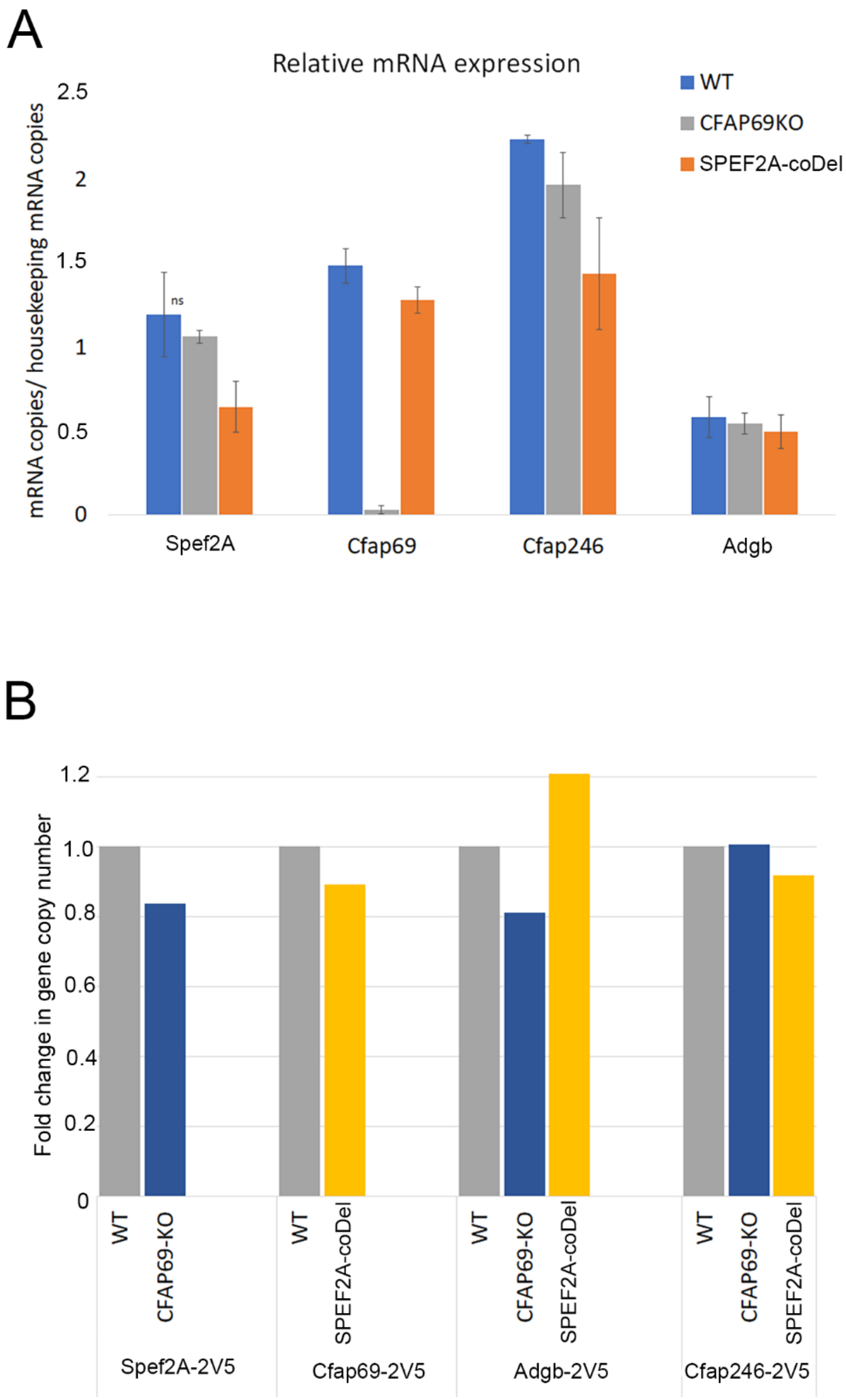

Supplementary Figure S6. Real-time PCR analyses of the levels of expression C1b/C1f proteins. Note a similar level of (**a**) transcripts of C1b components in wild-type and mutant cells (**b**) assortment of the transgenes enabling expression of the V5-tagged proteins, introduced to the macronuclear genome of either wild-type or mutant cells. *PF6* (encodes subunit of the C1a projection) and *CFAP251* (encodes radial spoke protein) were used as housekeeping genes.

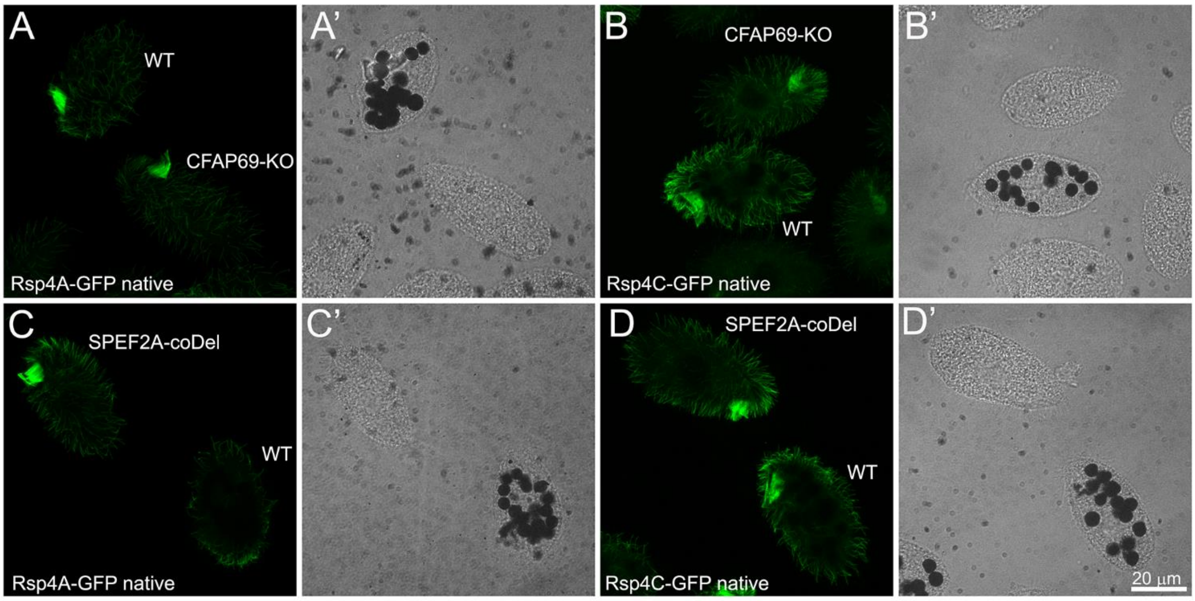

Supplementary Figure S7. Expression of the GFP-tagged radial spoke head proteins under the control of the respective native promoters, does not affect cells' motility. (**a-d'**) Confocal (**a**, **b**, **c**, **d**) and corresponding phase-contrast (**a'**, **b'**, **c'**, **d'**) images of wild-type (with dark, filled with India ink food vacuoles) and mutant cells, either *CFAP69-KO* (**a-b'**) or *SPEF2A-coDel* (**c-d'**) expressing either Rsp4/6A-GFP (**a**, **a'**, **c**, **c'**) or Rsp4/6C-GFP (**b**, **b'**, **d**, **d'**) under the control of the respective native promoter in native locus.

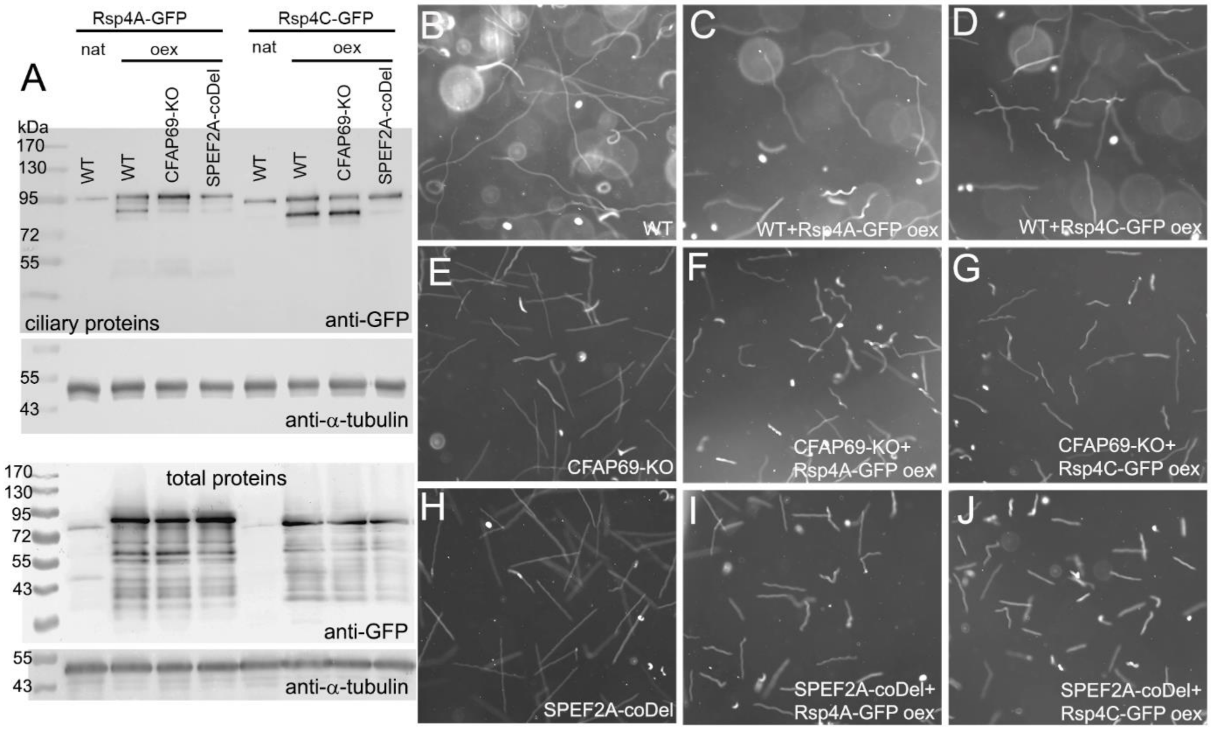

Supplementary Figure S8. Overexpression of GFP-tagged radial spoke head proteins slows down cells' motility. (**a**) A western blot analysis of the level of either Rsp4/6A-GFP or Rsp4/6C-GFP in cilia of wild-type and mutant cells. nat – an expression of the Rsp4/6A-GFP or Rsp4/6C-GFP under the control of the respective native promoter, oex – an overexpression of either Rsp4/6A-GFP or Rsp4/6C-GFP under the control of cadmium inducible *MTT1* promoter. Note that in some samples Rsp4/6 protein is partly degraded (two bands visible). Tubulin was used as a loading control. To detect GFP tagged protein 30 μm of ciliary proteins were loaded, to detect α-tubulin, 2 μg of ciliary proteins were loaded. (**b**-**j**) Swimming paths of either wild type (**b-d**) or *CFAP69-KO* mutant cells (**e-g**) or *SPEF2A-coDel* (**h-j**) overexpressing additionally Rsp4/6A-GFP (**c**, **f**, **i**) or Rsp4/6C-GFP (**d**, **g**, **j**). All swimming paths were recorded for 3.2 s at RT using the video camera. Bar = 400 μm.

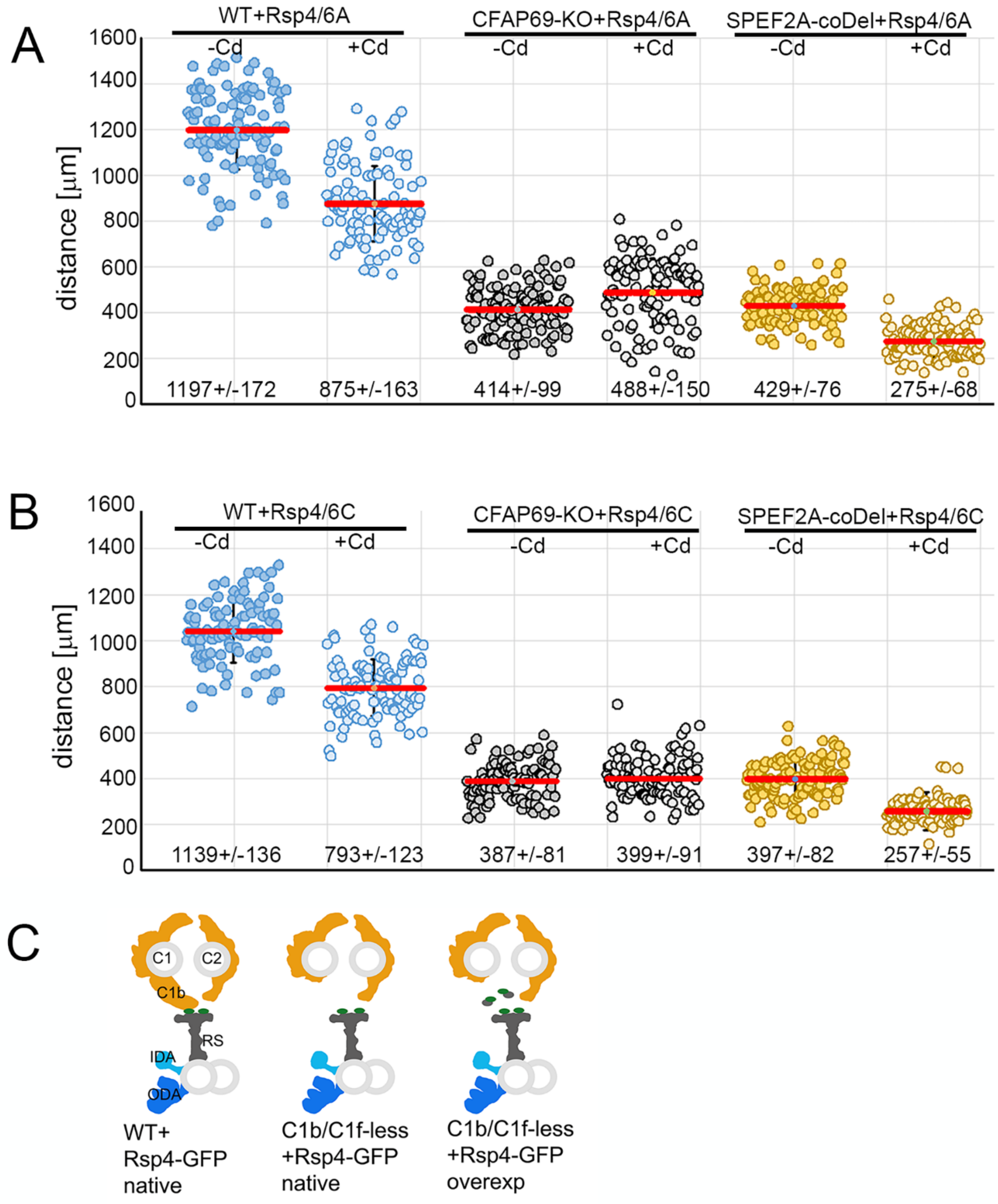

Supplementary Figure S9. Overexpression of GFP-tagged radial spoke head proteins does not restore mutant cells motility. (**a, b**) Graphs showing the comparison of the distances swum during 3.2 s by wild-type (WT) and analyzed mutants either non-induced (-Cd) or induced to overexpress (+Cd) radial spoke head protein, Rsp4/6A-GFP (**a**) or Rsp4/6C-GFP (**b**). Error bars represent standard deviation. Numbers below the graph indicate the distance swum by

the analyzed cells. Number of measurements: WT (n=96), WT+Rsp4A (n=103), CFAP69-KO+Rsp4A (n=105), others (n=100). Except for FAP69-KO+Rsp4/6C -/+Cd, the observed differences are statistically significant (p<0.01, t-test) (**c**) A schematic representation of the ultrastructural alterations in cilia in cells expressing or overexpressing GFP-tagged proteins (C1, C2 – central microtubules, C1b – projection; RS - radial spoke, IDA – inner dynein arm, ODA – outer dynein arm, green ovals - probable localization of GFP tag, grey ovals – overexpressed GFP-tagged Rsp4/6).

Supplementary Tables

Supplementary Table S1. List of primers used in this study.

Supplementary Table S2. Mass-spectrometry analysis of proteins biotinylated in cells expressing Spef2A-HA-BirA*.

Supplementary Table S3. Mass-spectrometry analysis of proteins biotinylated in cells expressing Cfap69-HA-BirA*.

Supplementary Table S4. Mass-spectrometry analysis of proteins biotinylated in cells expressing Cfap246 -HA-BirA*.

Supplementary Table S5. Mass-spectrometry analysis of the ciliary proteins that co-immunoprecipitated with Cfap69-3HA or Adgb-3HA, both expressed at native levels.

Supplementary Table S6. Mass-spectrometry analysis of the selected proteins in cilia assembled by wild-type, *CFAP69-KO* and *SPEF2A-coDel* mutants.

Supplementary Movies:

Supplementary Movie S1. High-speed video recording of beating cilia in wild-type cells

Supplementary Movie S2. High-speed video recording of beating cilia in *CFAP69-KO* cells

Supplementary Movie S3. High-speed video recording of beating cilia in *SPEF2A-coDel* cells

Supplementary Movie S4. High-speed video recording of beating cilia in *CFAP246-coDel* cells

Supplementary Movie S5. High-speed video recording of beating cilia in *ADGB-coDel* cells
