# Supplementary material for: Composition and function of the C1b/C1f region in the ciliary central apparatus
Source: Sci Rep. 2021 Jun 3;11:11760. doi: 10.1038/s41598-021-90996-9 (PMC8175508; doi:10.1038/s41598-021-90996-9)
Supplement: Supplementary file 2 — Supplementary Information 2. [file 41598_2021_90996_MOESM2_ESM.docx]

**Supplementary Table 1**

List of primers used in this study. The nucleotide sequences recognized by the restriction endonucleases are in bold (restriction enzyme name is a part of the primer name), the restriction sites introduced to screen for positive clones are in small letters, start and stop codon are underlined.

| **Primer name** | **Nucleotide Sequence** | **PCR product and Transgene** |
| --- | --- | --- |
| **C-terminal -3HA, -2V5 and -HA-BirA* native locus expression** | | |
| Spef2A-ORF-F-MluI | AATT **ACGCGT** TAATGAAAGCTCAATCATAGCTCC | 1.1 kb of a C-terminal fragment of the ORF without a stop codon  0.56 kb of a 3’UTR  transgene enabling expression of Spef2A with a C-terminal tag in a native locus under a native promoter |
| Spef2A-ORF-R-BamHI | AATT **GGATCC** AGAAAGAAGTAAAT  CTGTATATGTAGTTATTTG |  |
| Spef2A-3UTR-F-PstI | AATT CTGCAG  GCATTTAATAGCTGGGCTTGTA |  |
| Spef2A-3UTR-R-XhoI | AATT **CTCGAG**  GTCCACTCATATTTCCCTAAAACC |  |
| Spef2B-ORF-F-MluI | AAAT **ACGCGT** GCTTATATTCAACCACTCGAACTAG | 0.8 kb of a C-terminal fragment of the ORF without a stop codon  1.1 kb of a 3’UTR  transgene enabling expression of Spef2B with C-terminal tag in a native locus under a native promoter |
| Spef2B-ORF-R-BamHI | AATT **GGATCC** CTCGAAGAGAATTT  GCCAGAAATATTCTTG |  |
| Spef2B-3UTR-F-PstI | AAAT **CTGCAG** TGAAATTTTAATT  GAAAGATTTCATAAAATAAAGTC |  |
| Spef2B-3UTR-R-XhoI | AATT **CTCGAG** AGATCTTTAGGTATAGGTAGCTTAGG |  |
| Cfap69-ORF-F-MluI | AATT **ACGCGT**  GTTGTTTGCAGCTGAGG | 2.07 kb of a C-terminal fragment of the ORF without a stop codon  0.9 kb of a 3’UTR  transgene enabling expression of Cfap69 with C-terminal tag in a native locus under a native promoter |
| Cfap69-ORF-R-BamHI | AATT **GGATCC** TTTGTGTACTTTA  TTACTCCTTATTATATCG |  |
| Cfap69-3UTR-F-PstI | AATT **CTGCAG**  CTGCTAATGCTAATGTATATTTTGAA |  |
| Cfap69-3UTR-R-XhoI | AATT **CTCGAG**  TGATGATTTGGATTTATCTTCATAA |  |
| Cfap246-ORF-F-MluI | AATT **ACGCGT**  GTAGAACTTGACTTAATGGGAAAT | 1.06 kb of a C-terminal fragment of the ORF without a stop codon  0.94 kb of a 3’UTR  transgene enabling expression of Cfap246 with C-terminal tag in a native locus under a native promoter |
| Cfap246-ORF-R-BamHI | AATT **GGATCC** ATTGCTCTTTAAA  TTTAGATTTTGATTAAC |  |
| Cfap246-3UTR-F-PstI | AATT **CTGCAG** TAATTGTGGAGTA  TGTATTTATTTATGTATA |  |
| Cfap246-3UTR-R-XhoI | AATT **CTCGAG** GATATCCGATAAG GTTATCAAACACTTTAG |  |
| Adgb-ORF-F-MluI | AAAT **ACGCGT** ATCATAGGACTAA TGAAATTAAAGCT TTC | 0.82 kb of a C-terminal fragment of the ORF without a stop codon  0.9 kb of a 3’UTR  transgene enabling expression of Adgb with C-terminal tag in a native locus under a native promoter |
| Adgb-ORF-R-BamHI | AATT **GGATCC**  TCTTTTACCCTTTTTACCAGCCTT |  |
| Adgb-3UTR-F-PstI | AAAT **CTGCAG** CTATTTACTTTCA  ACTAAACAAATTGACATATT |  |
| Adgb-3UTR-R-XhoI | AATT **CTCGAG** AAGCTTATGATA  CTCATTCAAAGTAACTTC |  |
| **N-terminal -3HA, -and -BirA*-HA native locus expression** | | |
| Adgb-5UTR-F-SacII | TTAA **CCGCGG** CATTTTATGTAT  CATTTTGTTTTTGATATCAC | 0.66 kb of a 5’UTR  0.9 kb of a N-terminal fragment of the ORF  transgene enabling expression of Adgb with N-terminal tag in a native locus under MTT1 promoter |
| Adgb-5UTR-R-PstI | AATT **CTGCAG** GCGAAATATATCC  TTATAAATAATAACAAGC |  |
| Adgb-ORF-F-MluI | AATT **ACGCGT**  T ATG CCTCCAAAGCAATAAAAATAG |  |
| Adgb-ORF-R-BamHI | AATT **GGATCC** TTAATAAGTTA  CAATTATGAGGCAATCC |  |
| **C-terminal -GFP native locus expression** | | |
| Rsp4/6A-ORF-F-MluI | AAAT **ACGCGT** CGTACTTTAATTGACCCTGATGAGA | 0.8 kb of a C-terminal fragment of the ORF without a stop codon  0.7 kb of a 3’UTR |
| Rsp4/6A-ORF-R-BamHI | AATT **GGATCC** ATTCTCTTCCTCTT  CTTCTTATTATTGCTATTGG |  |
| Rsp4/6A-3UTR-F-PstI | AAAT **CTGCAG** CTATCTATCAA  TCGATATATGTATGTATGGTTTC |  |
| Rsp4/6A-3UTR-R-XhoI | AATT **CTCGAG** AATATAAAAGTG CAAACGAAATCATTAATTTTG |  |
| Rsp4/6C-ORF-F-MluI | ATTT **ACGCGT** GTATTAAGTTTGGA TGACCCTG | 0.74 kb of a C-terminal fragment of the ORF without a stop codon  0.8 kb of a 3’UTR |
| Rsp4/6C-ORF-R-BamHI | AATT **GGATCC** TTCTTATTCTTCTTA AGCATTCTCTTCATTTTCTTC |  |
| Rsp4/6C-3UTR-F-PstI | AAAT **CTGCAG** GCTTTAAATACATA  TTAAATACATGTAATTATAAC |  |
| Rsp4/6C-3UTR-R-XhoI | AATT **CTCGAG** ATATCACTGTAAA CCTTGTAATATTACTAG |  |
| **C-terminal GFP overexpression in *BTU1* locus** | | |
| Rsp4/6A -F-MluI | AATT **ACGCGT** T ATG AGTTCTTAATTAAAGGCTTAATTAGAT | entire ORF (2.46 kb) without a stop codon |
| Rsp4/6A-ORF-R-BamHI | AATT **GGATCC** ATTCTCTTCCTCTT  CTTCTTATTATTGCTATTGG |  |
| Rsp4/6C -F-MluI | AATT **ACGCGT** T ATGTAATAAGA TTCTTAAAAGAAGCTCATTAAT | entire ORF (2.46 kb) without a stop codon |
| Rsp4/6C-ORF-R-BamHI | AATT **GGATCC** TTCTTATTCTTCTTA AGCATTCTCTTCATTTTCTTC |  |
| **Gene deletion** | | |
| Cfap69-KO-5-F-ApaI | AATT **GGGCCC**  ACTCAAGGAAATGCAGTCG | 1.2 kb fragment of the open reading frame, upstream to the deleted gene fragment (0.78 kb)  1.5 kb fragment encompassing 1.3 kb of the open reading frame and 0.2 kb of 3’UTR downstream to the deleted gene fragment  Transgene to knockout *CFAP69* gene |
| Cfap69-KO-5-R-SmaI | AATT **CCCGGG**  CCTCAGCTGCAAACAACTA |  |
| Cfap69-KO-3-F-PstI | AATT **CTGCAG**  GCTTAAGAGTATTCAACAATGCTT |  |
| Cfap69-KO-3-R-SacII | AATT **CCGCGG**  TCATTTGTGTACTTTATTACTCCTT |  |
| Spef2A-coDel-F | CAGTTC TCATCA AGTTGT AATGCT AAAATG CGGCCG CGATAC AAGAAA AGCTAA GATGAC TAACTT | 0.8 kb of the open reading frame  used to prepare a transgene enabling gene deletion using coDel approach |
| Spef2A-coDel-R | GGACTC TTTATT GTTATC ATCTTA TGACCG CGGCCG CCTCGA GCATCT TCATCT TCTTCA AGCAAG AG |  |
| Cfap246-coDel-F | CAGTTC TCATCA AGTTGT AATGCT AAAATG CGGCCG CCTGTC TTCCAA CAAAAT ATCTAA GTAAG | 0.68 kb of the open reading frame  used to prepare a transgene enabling gene deletion using coDel approach |
| Cfap246-coDel-R | GGACTC TTTATT GTTATC ATCTTA TGACCG CGGCCG CCTCGA GCATTG TCACTT TCAGTT TCAGGT |  |
| Adgb-coDel-F | CAGTTC TCATCA AGTTGT AATGCT AAAATG CGGCCG CGATGA TAAGAT GCCAGT TAACTT TAAAG | 0.76 kb of the open reading frame  used to prepare a transgene enabling gene deletion using coDel approach |
| Adgb-coDel-R | GGACTC TTTATT GTTATC ATCTTA TGACCG CGGCCG CCTCGA GCAACT TCCTCT TCATCA GCTTC |  |
| Adgb-KO-5-F-ApaI | AATT **GGGCCC** TACTCCTTCCTTTGTTAATGACAATT | 0.94 kb fragment of the open reading frame, upstream to the deleted gene fragment (0.75 kb)  1.42 kb fragment of the open reading frame downstream to the deleted gene fragment  Transgene to knockout *ADGB* gene |
| Adgb-KO-5-R-SmaI | AATT **CCCGGG**AAATTTGCATTAAC  CTTTAAAGTTAACT |  |
| Adgb-KO-3-F-PstI | AATT **CTGCAG** CATGTCAATTCAAAACCAATC |  |
| Adgb-KO-3-R-SacII | AATT **CCGCGG** TTCATTGTCTTCTCCTGTTGTTATTTC |  |
| **Primers used to verify deletion in the targeted locus** | | |
| Spef2A-check-F | CAACACAATATCCAATTTTATTAACAAAG | Primers used to verify deletion of the fragment of *SPEF2A* gene |
| Spef2A-check-R | ATAACCTAGAATTGCAAGTTTTAAAG |  |
| Spef2A-check-ORF-F | ATACATATTTATTCGATCGCCCTAG |  |
| Cfap246-check-F | GAGGATAACAAATTAATCAATTGGA | Primers used to verify deletion of the fragment of *CFAP246* gene |
| Cfap246-check-R | AAA TCA ATG ATT CTG TAA ACT CTG |  |
| Adgb-check-F | AGATATGAAGATTGCCAATCAACT | Primers used to verify deletion of the fragment of *ADGB* gene |
| Adgb-check-R | TTGATAAGCATTATTTATTCGGAATG |  |
| **Real time PCR** | | |
| Tt-Spef2-EX 12-13-FW | CCATAAAGACCTAGAAACAGATTTTT | amplified fragment of 125 bp |
| Tt-Sepf2-EX13-RV | CAAGAAAGAAGTAAATCTGTATATGTAGT |  |
| Tt-CFAP69-EX4-FW | TGGTCGAATCCTTAGCTAAAAGAA | amplified fragment of 143 bp |
| Tt-CFAP69-EX5-RV | TCAGGATCATCTTGATTTGTTAAGA |  |
| Tt-ADGL-EX13-FW | ACA CTT AGC TGA TCA CCC AG | amplified fragment of 141 bp |
| Tt-ADGL-EX14-RV | TGC TTT AAC TCT TCA CGA AGC |  |
| Tt-CFAP246-EX4 FW | ACT TGA CAT TCCTTTCTGATTTAGA | amplified fragment of 132 bp |
| Tt-CFAP246-EX5RV | ATT CTT CAG AAG TGG TAT TGA CAC |  |
| **Housekeeping genes** | | |
| Tt-PF16-EX2-FW | CGA TGA ATC ACT TGC CAT GA | amplified fragment of 140 bp |
| Tt-PF16-EX3-RV | TGT GGC CAC CAA TTT ATC CA |  |
| Tt-CFAP251-EX14-FW | TTA ACC ACT GAA GGA GAG CG | amplified fragment of 155 bp |
| Tt-CFAP251-EX16-RV | TCA CCT TCT AAA TCT TCA AAA CCT |  |
| **Primers used to check level of transgenes assortment in CFAP69 and SPEF2A knockouts** | | |
| BTU1coding-F | CTT TCT TAC ATT GGT ACA CTG GTG | amplified fragment of 274 bp |
| BTU1-3UTR-RV | GTGAATGAAGTTAATTGGGTATTTCT |  |
| F246coding-FW | CCAACTTTTAAGAGTCACAATCAAG | amplified fragment of ~290 bp |
| ANDcoding-FW | AAATTCAGTGTAACAGGTATTGATG | amplified fragment of ~260 bp |
| F69coding-FW | TGTATAAATACACAGAGCTTGGTAA | amplified fragment of ~280 bp |
| SPEF2Acoding-FW | ATA CAT ATT TAT TCG ATCGCCCTAG | amplified fragment of 365 bp |
| **Primers used to engineer Cfap69 and Spef2A truncations** | | |
| Tt-CFAP69-MluI-Fw | AATT **ACGCGT** C ATG AGT ACT CTA GGA AAT ACT CAA GGA A | Fragment of the coding region |
| Tt-CFAP69-BamHI-R248Ter_RV | ATA T **GG ATC C**TC AAG AAC GGA AAT CAT CAC AGT CAA AA |  |
| Tt-Spef2-MluI-Fw | AATT ACGCGT C ATG AAT GAA ATA AAT AAT AAA ATA CAA GCA A | Fragment of the coding region |
| Tt-Spef2-M303Ter-BamHI-Rv | ATA T **GG ATC C** TC ATT TTT TAA GAG TTT CTT CAA ACA TGT C |  |
